# Digital Psychotherapies for Adults Experiencing Depressive Symptoms: Systematic Review and Meta-Analysis

**DOI:** 10.2196/55500

**Published:** 2024-09-30

**Authors:** Joanna Omylinska-Thurston, Supritha Aithal, Shaun Liverpool, Rebecca Clark, Zoe Moula, January Wood, Laura Viliardos, Edgar Rodríguez-Dorans, Fleur Farish-Edwards, Ailsa Parsons, Mia Eisenstadt, Marcus Bull, Linda Dubrow-Marshall, Scott Thurston, Vicky Karkou

**Affiliations:** 1 School of Health and Society University of Salford Manchester United Kingdom; 2 Faculty of Health, Social Care and Medicine Edge Hill University Ormskirk United Kingdom; 3 Faculty of Nursing, Midwifery and Palliative Care King's College London London United Kingdom; 4 School of Health and Social Science University of Edinburgh Edinburgh United Kingdom; 5 Evidence Based Practice Unit University College London London United Kingdom; 6 Faculty of Life Sciences and Education University of South Wales Newport United Kingdom

**Keywords:** digital psychotherapies, depression, adults, systematic review, meta-analysis, mobile phone

## Abstract

**Background:**

Depression affects 5% of adults and it is a major cause of disability worldwide. Digital psychotherapies offer an accessible solution addressing this issue. This systematic review examines a spectrum of digital psychotherapies for depression, considering both their effectiveness and user perspectives.

**Objective:**

This review focuses on identifying (1) the most common types of digital psychotherapies, (2) clients’ and practitioners’ perspectives on helpful and unhelpful aspects, and (3) the effectiveness of digital psychotherapies for adults with depression.

**Methods:**

A mixed methods protocol was developed using PRISMA (Preferred Reporting Items for Systematic Reviews and Meta-Analyses) guidelines. The search strategy used the Population, Intervention, Comparison, Outcomes, and Study Design (PICOS) framework covering 2010 to 2024 and 7 databases were searched. Overall, 13 authors extracted data, and all aspects of the review were checked by >1 reviewer to minimize biases. Quality appraisal was conducted for all studies. The clients’ and therapists’ perceptions on helpful and unhelpful factors were identified using qualitative narrative synthesis. Meta-analyses of depression outcomes were conducted using the standardized mean difference (calculated as Hedges *g*) of the postintervention change between digital psychotherapy and control groups.

**Results:**

Of 3303 initial records, 186 records (5.63%; 160 studies) were included in the review. Quantitative studies (131/160, 81.8%) with a randomized controlled trial design (88/160, 55%) were most common. The overall sample size included 70,720 participants (female: n=51,677, 73.07%; male: n=16,779, 23.73%). Digital interventions included “stand-alone” or non–human contact interventions (58/160, 36.2%), “human contact” interventions (11/160, 6.8%), and “blended” including stand-alone and human contact interventions (91/160, 56.8%). What clients and practitioners perceived as helpful in digital interventions included support with motivation and accessibility, explanation of task reminders, resources, and learning skills to manage symptoms. What was perceived as unhelpful included problems with usability and a lack of direction or explanation. A total of 80 studies with 16,072 participants were included in the meta-analysis, revealing a moderate to large effect in favor of digital psychotherapies for depression (Hedges *g*=–0.61, 95% CI –0.75 to –0.47; *Z*=–8.58; *P*<.001). Subgroup analyses of the studies with different intervention delivery formats and session frequency did not have a statistically significant effect on the results (*P*=.48 and *P*=.97, respectively). However, blended approaches revealed a large effect size (Hedges *g*=–0.793), while interventions involving human contact (Hedges *g*=–0.42) or no human contact (Hedges *g*=–0.40) had slightly smaller effect sizes.

**Conclusions:**

Digital interventions for depression were found to be effective regardless of format and frequency. Blended interventions have larger effect size than those involving human contact or no human contact. Digital interventions were helpful especially for diverse ethnic groups and young women. Future research should focus on understanding the sources of heterogeneity based on intervention and population characteristics.

**Trial Registration:**

PROSPERO CRD42021238462; https://www.crd.york.ac.uk/prospero/display_record.php?RecordID=238462

## Introduction

### Background

Globally, depression affects >280 million people including 5% adults [1]. It is a leading cause of disability worldwide and a major contributor to the overall global burden of disease [2]. In the United Kingdom, for example, depression affects 3% to 6% of people [3] from diverse socioeconomic, educational, and cultural backgrounds.

In view of the prevalence of depression across different groups, the 2022 National Institute for Health and Care Excellence (NICE) guidelines for depression [3] have been revised to include a range of interventions as a first line of treatment, thus meeting diverse needs while widening clients’ choices. The interventions include behavioral activation, exercise, mindfulness, cognitive behavioral therapy (CBT), counseling for depression, psychodynamic psychotherapy, and couples therapy. In addition, the NICE guidelines [3] recommended antidepressant medication but for severe depression only, placing an emphasis on clients’ preferences and the role of psychological and physical interventions over drug treatment.

In the past, interventions for depression were provided predominantly face to face, and digital psychotherapies were available only to a limited degree. However, the outbreak of COVID-19 and consequent lockdowns, social distancing, and isolation rules have led to many people struggling with anxiety and depression [4]. The National Health Service had to adapt to provide therapy in a more flexible way, including digital delivery [5], which was in line with the policies outlined in the UK Government’s Five Year Forward View for Mental Health [6] and the National Health Service Long Term Plan [7]. This has led to a need for more research in relation to the provision of digital psychotherapies.

The need for guidance in digital psychotherapies has given rise to recent systematic reviews on depression. However, the reviews are limited to formal diagnoses of unipolar depression [8] or relate to chronic health conditions [9-11]. The available reviews also focus on specific modes of delivery (eg, smartphone apps [12]) or specific client groups (eg, perinatal clients [13] or children and young people [14-17]). Another limitation of most of the published systematic reviews is a focus on CBT only [11,18-20] ignoring the fact that a range of interventions are being offered for depression [3]. A broader review of evidence in relation to a range of digital approaches and psychotherapeutic theories is needed, and this systematic review aims to address this gap.

Most recent meta-analyses highlighted that there is no difference between technology-based and in-person treatments for depression [21], and there are some indications [22] that this is reflected in clients’ preferences for treatment: 55.5% of adults choose digital psychotherapy for depression. However, practitioners draw attention to the therapeutic relationship in digital psychotherapies, which is important to consider [21]. There is some evidence that working digitally does not reduce the quality of the therapeutic relationship [23], but therapists are often concerned that they do not have the same access to the clients’ experience as in face-to-face interactions. Researchers argue that most therapeutic activity is grounded in the body involving body-to-body communication, attunement, and coregulation of feelings in the shared physical space, which cannot be replaced by a web-based treatment [24]. A preliminary literature search revealed no systematic reviews highlighting service users’ perspectives on digital psychotherapies for depression. This would seem essential when discussing individual preferences and ethical considerations of web-based treatments especially in terms of safety and privacy [25]. This current systematic review aims to address this gap and present service users’ perspectives within the existing research on digital psychotherapies for depression, highlighting their needs and preferences.

An additional problem in existing literature is a lack of clarity in the terminology used for the digital modes of delivery, leading to confusion in relation to what is effective and what is not [26]. Delivery can be, for example, asynchronous, synchronous, self-guided, with a therapist, or blended. Types of media can include telephone, videoconferencing, emails, websites, or apps. In the literature, terms are often used inconsistently. Therefore, the current systematic review will review the terminology and summarize current evidence using consistently defined terms.

Moreover, it is important to understand the specific factors impacting the effectiveness of digital interventions for depression. There are arguments that effectiveness depends on the duration of the intervention, baseline severity, adherence, and the level of human guidance [19]. This current systematic review will focus on these and other important factors within the data set, aiming to provide more specific guidance in relation to the digital psychotherapies for depression.

### Research Questions

Taking the above issues into account, this systematic review will aim to answer the following 3 research questions that have not yet been addressed in other systematic reviews. The research questions will include:

1. What are the most common types of digital psychotherapeutic interventions for adults with depression?
2. What are the clients’ and practitioners’ perspectives on helpful and unhelpful factors in digital psychotherapeutic interventions for adults with depression?
3. What is the effectiveness of digital psychotherapeutic interventions for adults with depression?

## Methods

### Overview

A mixed methods systematic review protocol was developed using PRISMA (Preferred Reporting Items for Systematic Reviews and Meta-Analyses) guidelines [27] and registered with PROSPERO (2021; CRD42021238462). Unlike traditional systematic reviews, this review combined both quantitative and qualitative studies. The intention was to maximize the findings not only by examining the effectiveness of digital psychotherapies but also by mapping the utility, impact, and the ability of those findings to inform policy and practice.

### Search Strategy

The search strategy was developed using 3 key concepts: population, intervention, and context (population: people with depression, intervention: digital interventions, and context: psychotherapy) using Boolean operators and truncation marks (Textbox 1). The following data bases were searched up to February 12, 2024: CINAHL, PsycArticles, PsycINFO, PubMed, BASE, Academic Search Premier, and ProQuest Health Research Premium Collection.

**Search strings.**

Step 1: “depress*” OR “dysthymi*” OR “adjustment disorder*” OR “mood disorder*” OR “affective disorder*” OR “affective symptom*”

AND

Step 2: “online” OR “remote” OR “tele-therap*” OR “digital” OR “e-mental health” OR “e-therap*” OR “mobile*” OR “internet-administered” OR “web-based” OR “app*” OR “digital*” OR “technolog*” OR “computer*” OR “tablet*” OR “m-health*” OR “mobile health” OR “e-health” OR “electronic health”

AND

Step 3: “Psychotherap*” OR “psychologic*” OR “therap*” OR “counselling” OR “counseling”

The search was limited to the 2010-to-2024 time frame in order to focus on the latest advances in digital psychotherapies.

### Screening

Search results were independently screened by 4 reviewers (SA, SL, JO-T, and RC) at the title and abstract level. The full texts were then assessed for eligibility based on the predetermined criteria that were set using a combination of the Population, Intervention, Comparison, Outcomes and Study Design (PICOS) framework [28] (Table 1) and other factors such as context, time period, and the type of publication. Where data needed for eligibility assessment were missing, the authors were contacted to provide the information. Unclear or unresolved cases were discussed and moderated during the weekly team meetings.

**Table 1:** Eligibility criteria.

Criteria	Inclusion	Exclusion
Population	Participants with a mean age of ≥18 years, any gender, ethnicity, country, and with any severity and chronicity of depression. ≥75% of participants in the study should have a diagnosis of depression or self-report depression or low mood as a primary reason for being involved in the study. The diagnosis could involve MDD^a^, dysthymic disorder, peripartum depression (previously postpartum depression), seasonal affective disorder, or premenstrual dysphoric disorder.	Studies where depression is not a primary outcome (comorbid with psychotic or other medical and mental health conditions) and acute phase of depression.
Intervention	Studies with digital psychotherapeutic intervention as the main intervention, including all forms of verbal psychotherapies and counseling (eg, humanistic, psychoanalytic or psychodynamic, cognitive or behavioral, and integrative); creative or arts psychotherapies (eg, dance, drama, art, music, and poetry); and any combination of the above delivered in any digital format (eg, websites, apps, telephone, videoconferencing, emails, etc)	Studies where ≥50% sessions are delivered nondigitally (eg, face-to-face consultation in the therapy room). S studies with advice, guidance, signposting, coaching, psychoeducation, and peer support. Studies focused on only screening, assessment, prevention, and follow- up.
Comparators	All types of comparators such as waiting list, treatment as usual, face-to-face psychological therapies, pharmacological interventions, physical interventions, or studies with no comparators	None
Outcomes	Depressive symptoms measured using any validated instruments (self-rated or observational tools) is considered as the primary outcome. In addition, views or perspectives of clients and practitioners on the processes and helpful and unhelpful factors or aspects of digital psychotherapies for adults with depression	None
Study design	Any type of empirical research with quantitative, qualitative, mixed, or arts-based approaches using surveys, pilot studies, intervention protocols, and quasi-experimental studies, RCTs^b^, interviews, and other methods with people experiencing depression is considered.	Systematic reviews, secondary sources, opinion-based articles, editorials, policy reviews and statements, and commentaries. Unpublished masters or doctoral level dissertations, unpublished conference presentations, conference proceedings where full-length articles are not available, clinical case examples without explicit research methodology, and narrative articles.
Context	Psychotherapeutic interventions delivered “digitally” by qualified and registered therapists or web-based interventions or apps informed by psychotherapeutic approaches.	Nontherapeutic studies, educational videoconferences, workshops, and self-help programs that involve exclusively chats or support groups.
Time period	2010-2024	Before 2010 and unpublished ongoing studies
Publication type	Peer-reviewed	Editorials, conference presentations, and opinion-based articles

^a^MDD: major depressive disorder.

^b^RCT: randomized controlled trial.

### Extraction

Studies involving both therapeutic processes and outcomes of digital psychotherapies were included to aid further understanding of the digital contribution to psychotherapies for depression. An extraction form based on a Microsoft Excel spreadsheet was developed to gather information for each research question. The review authors piloted 8 initial studies to refine the extraction form. A total of 10 authors independently extracted data from approximately 10 to 30 studies each, and 4 authors (SA, SL, JO-T, and RC) cross-checked and verified all the extracted data. Disagreements were resolved by discussion in weekly meetings, and when issues remained unclear, the members of the review team arbitrated. In the first instance, the team extracted demographic data related to the studies and population characteristics. To answer the first research question in relation to the most common types of digital interventions for depression, we used the Template for Intervention Description and Replication (TIDieR), which is a commonly used intervention description and replication checklist [29]. Furthermore, clients’ and practitioners’ perceptions on helpful and unhelpful factors of digital psychotherapies were gathered to answer the second research question. Finally, numeric data related to the primary outcomes of depression were documented for the third research question.

### Quality Assessment

Randomized controlled trials (RCTs) were evaluated using the Cochrane risk-of-bias assessment tool [30] to identify risks such as selection bias (random sequence generation and allocation concealment), performance bias (blinding of participants and personnel), detection bias (blinding of outcome assessment), attrition bias (incomplete outcome data), and reporting bias (selective reporting). Studies with non-RCT designs (eg, controlled trials, pretest-posttest design, mixed methods, and qualitative studies) were evaluated using the Mixed Methods Appraisal Tool (MMAT; version 2018) [31]. The review authors working in pairs answered the questions that covered various aspects of the quality of execution and reporting of the studies.

### Data Synthesis and Analysis

All the studies that met the inclusion criteria were considered for qualitative narrative synthesis [32] to present the characteristics of digital psychotherapies. The synthesis began with the “mapping” of the available relevant evidence against the specific research questions. Intervention-specific and person-specific factors influencing digital psychotherapy were explored and thematically analyzed using a modified behavior change model [33], which provides a useful classification of the barriers and facilitators in digital interventions [34]. Moreover, the quantitative data from studies with RCT components were analyzed to evaluate the pooled robustness of the digital psychotherapy outcomes.

Meta-analyses of depression outcomes were conducted using the standardized mean difference (SMD, calculated as Hedges *g*) of the postintervention change between digital psychotherapy and control groups to accommodate for expected methodological and intervention design variations [35]. Analyses were conducted for all depression outcomes combined, and the precision of the SMD was calculated for each trial by the 95% CI. A negative SMD implied better therapeutic effects over time in the digital psychotherapy group compared to the control group. All the analyses were performed using Comprehensive Meta-Analysis software. The pooled effect sizes were interpreted using the same rule for describing Cohen *d* effect sizes as applied to Hedges *g*. SMDs of ≤0.30, 0.30 to 0.60, and >0.60 were considered as small, moderate, and large effect sizes, respectively.

We intended to include crossover trials but only the first active treatment period. For studies with multiple arms, only those with the digital intervention and the control arms were included in the analysis [36]. If there were 2 digital intervention arms with a single control group, then the sample size of the control group was halved before the meta-analysis to avoid counting the same participants twice. When studies presented data from >1 depression measurement tool, we prioritized data in the following order: Beck Depression Inventory, Patient Health Questionnaire-9, and Hamilton Depression Rating Scale based on the most frequently used tools identified in this review.

### Dealing With Missing Data

For meta-analysis where mean, SD, and sample size were missing from the end of intervention scores, we looked for alternate formats available on Comprehensive Meta-Analysis to compute the missing data. If we lacked sufficient information to extrapolate missing information, we contacted the study authors to obtain the missing data and the reason for the missing data. Where this was not possible, we excluded the study from the meta-analysis but used the descriptive information to answer other research questions and qualitative synthesis.

### Assessment of Heterogeneity

We initially explored heterogeneity across studies using a visual inspection of forest plots (potential heterogeneity was considered where CIs were not overlapping). Furthermore, to assess the presence and extent of between-study variation *I*^2^ statistic with 95% CIs (uncertainty), *Q* statistics were used. The *Q* test was performed to check if there was any variance in the true effect size across studies with an α criterion set at a low statistical power of .100 for a better possibility to reject the null hypothesis and identify if the effect sizes varied across studies. The *I*^2^ test was performed to identify what proportion of the variance in observed effects shows variance in true effects rather than a sampling error.

### Assessment of Reporting Bias

For evaluating the risk of publication bias, funnel plots for overall depression outcomes were visually inspected for asymmetry (ie, SMDs charted against their SE). As ≥10 studies were pooled, we formally tested funnel plot asymmetry using Egger test of the intercepts [37]. A positive intercept indicates that studies with smaller sample sizes tend to report more positive results than large-sample studies. When the test found notable asymmetry (*P*=.10), we reported primary outcomes based on a fixed effects model along with a random effects model. This strategy gave more weight to larger trials and helped to counterbalance a possible inflation of the therapeutic effect by discussing a more conservative effect estimate [38].

### Subgroup Analysis

Subgroup meta-analyses were conducted to investigate between-study variability, explore the reasons for heterogeneity, and recognize intervention design components that may moderate observed efficacy. Subgroup analyses were based on a mixed effects model, which used a random effects model to generate within-subgroup variance and a fixed effects model to compare effects between subgroups [39]. Between-subgroup heterogeneity was tested using Cochrane *Q* statistic and was considered significant at the *P*=.05 level. The following moderating factors related to the intervention were included in our analysis plan: delivery format of the digital intervention (contact with human, no contact with human, and blended) and session frequency (once per week and more than once per week).

## Results

### Overview

**Figure 1:**
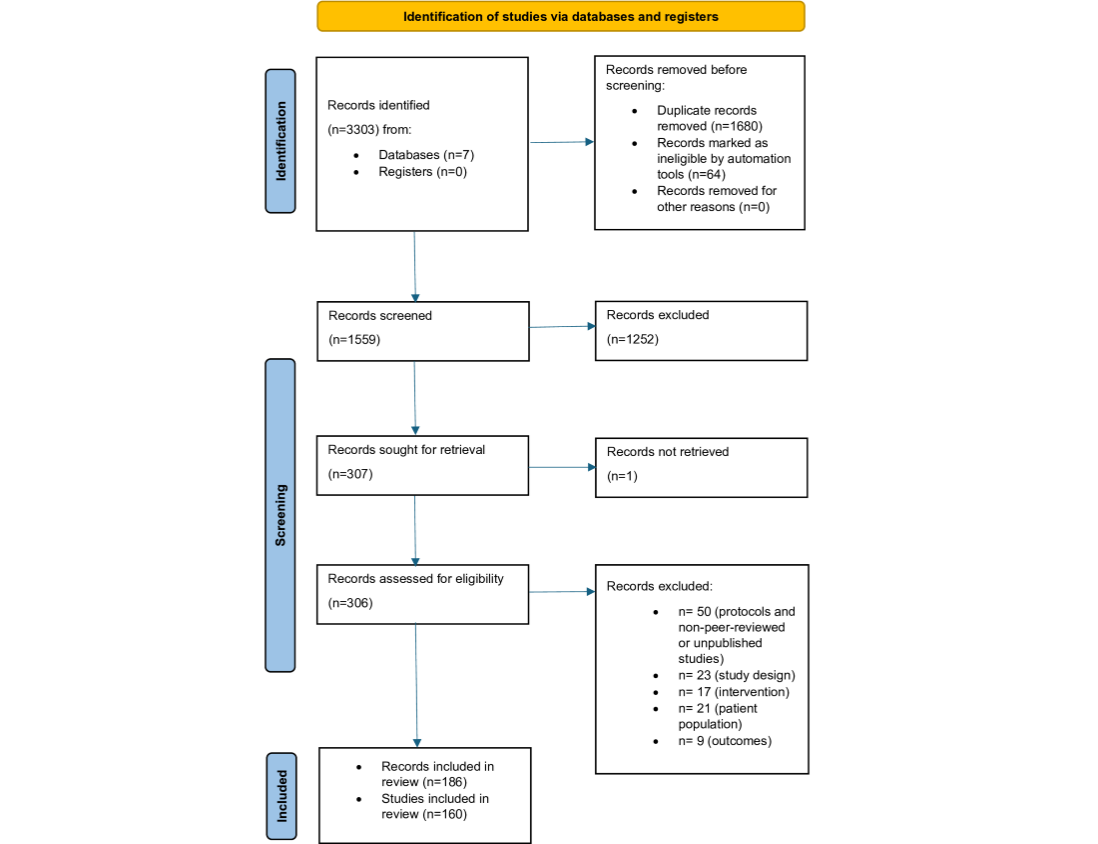
PRISMA (Preferred Reporting Items for Systematic Reviews and Meta-Analyses) 2020 flow diagram for new systematic reviews that included searches of databases and registers only.

As shown in the PRISMA flow diagram (Figure 1), the comprehensive search of 7 academic databases resulted in 3303 records. After duplicates were removed, the remaining 1559 (47.2%) records were screened at the title and abstract level, excluding 1252 (80.31%) records. Out of the remaining 307 articles, 306 (99.7%) were read in their entirety, while 1 (0.3%) study was not retrieved. A total of 23 (7.5%) records were excluded based on the study design, 21 (6.9%) in relation to population, 17 (5.5%) due to intervention, 9 (2.9%) based on outcomes, and 50 (16.3%) due to the type of publication. The remaining 186 (60.8%) records (Multimedia Appendix 1 [40-224]), corresponding to 160 studies, were included for data extraction. Of these, 25 (13.4%) records constituted sibling studies that formed 18 groups of studies using the same samples. To avoid double counting of data, only the main publication was considered for demographic data extraction and research questions. Data from sibling studies were mainly extracted in relation to clients’ and practitioners’ perspectives on helpful and unhelpful aspects of digital psychotherapies. In terms of meta-analysis of the overall efficacy of the digital psychotherapy for adults with depression, data from 80 RCTs were considered.

### General Characteristics of Included Studies

#### Study Characteristics

#### Year of Publication

This systematic review covers the period between 2010 and February 2024. Most papers were published in 2018 (21/160, 13.1%) and 2020 (18/160, 11.2%). In the years 2023, 2021, 2013, and 2014, a total of 13 (8.1%) or 14 (8.8%) papers were published each year. The remaining years saw 3 to 10 papers yearly.

#### Country

A total of 31 countries were covered in the studies (n=160) including the United States (n=49, 30.6%), the United Kingdom (n=18, 11.2%), Australia (n=15, 9.4%), Germany (n=13, 8.1%), the Netherlands (n=12, 7.5%), Canada (n=9, 5.6%), and others (n=40, 25%). Most of the studies (n=116, 72.5%) were conducted in countries from the global north (eg, the United States, the United Kingdom, the Netherlands, Australia, Germany, and Canada). Furthermore, 2 (1.25%) studies included 2 sites, Australia and the United States, and 3 (1.8%) studies were multinational.

#### Methodological Approach

The studies (n=160) were conducted using a range of approaches but predominantly adopted quantitative approaches (n=131, 81%), out of which more than half of the studies (n=88, 55% were RCTs. A total of 15 (9.4%) studies were conducted using mixed methods designs where 3 (20%) studies had an RCT component. Only 13 (8.1%) of the studies provided solely a qualitative view of the digital therapeutic process.

#### Place of Recruitment

A total of 61 (38.1%) studies recruited participants from health care settings, 44 (27.5%) used online methods, 15 (9.4%) were from community settings, 12 (7.5%) from educational settings, 14 (8.8%) from other settings, and 14 (8.8%) from a combination of the above.

The “other” category included a telephone service, postal invitations, emailed letters, and places of employment. The most common combination of recruitment places was “online and other” with 3 (1.9%) studies using this combination (Table 2).

**Table 2:** Study characteristics (n=160).

Characteristic				Frequency, n (%)
**Year of publication**				
	2018			21 (13.1)
	2020			18 (11.2)
	2023, 2013			14 (8.8)
	2014			13 (8.1)
	2021			12 (7.5)
	2019, 2016			10 (6.2)
	2017, 2015			9 (5.6)
	2022			8 (5)
	2012, 2010			7 (4.4)
	2011			5 (3.1)
	2024			3 (1.9)
**Country**				
	The United States			49 (30.6)
	The United Kingdom			18 (11.2)
	Australia			15 (9.4)
	Germany			13 (8.1)
	The Netherlands			12 (7.5)
	Canada			9 (5.6)
	Austria, China, Iran, Ireland, multinational, and Switzerland			3 (1.9)
	Australia and United States, Denmark, Finland, Japan, New Zealand, Norway, Japan, and Sweden			2 (1.2)
	Azerbaijan, Brazil, Egypt, India, Mexico, Nigeria, Oman, the Republic of Korea, Romania, South Africa, Spain, and Turkey			1 (0.6)
**Methodological approach**				
	**Quantitative**			
		RCT^a^	88 (55)	
		Non-RCT	18 (11.2)	
		Descriptive	25 (15.6)	
		Mixed methods	15 (9.4)	
	Qualitative			13 (8.1)
	Other			1 (0.6)
**Place of recruitment**				
	Health care settings			61 (38.1)
	Online			44 (27.5)
	Community settings			15 (9.4)
	Other and combination			14 (8.8)
	Educational settings			12 (7.5)

^a^RCT: randomized controlled trial.

#### Population

##### Sample Size

A total of 160 studies (186 articles) encompassed a sample of 70,720 participants (Table 3).

**Table 3:** Participant characteristics (n=70,720).

Characteristics			Value
Age (y), mean (SD; range)			38.52 (10.87; 16-89)
**Gender** **, n (%)**			
	Woman	51,677 (73.07)	
	Man	16,779 (23.73)	
	Not reported	2147 (3.04)	
	Other	72 (0.1)	
	Transgender	18 (0.03)	
	Nonconforming, queer	9 (0.01)	
	Variant	5 (0.007)	
	Gender Expansive	2 (0.002)	
	Gender Fluid	1 (0.001)	
	Nonbinary	1 (0.001)	
**Race and ethnicity^a^, n (%)**			
	African, First Nations or Metis	4 (0.01)	
	African American	262 (0.65)	
	African Canadian, Black and White, Indigenous, Latin American, Middle Eastern, and New Zealand Indian	1 (0.002)	
	American	2 (0.005)	
	American Indian or Alaskan Native	23 (0.06)	
	Asian	450 (1.11)	
	Asian American	3 (0.01)	
	Black	338 (0.83)	
	Black African American	136 (0.34)	
	Caucasian	1241 (3.06)	
	Chinese	90 (0.22)	
	Dutch	667 (1.65)	
	European	105 (0.26)	
	European New Zealander	41 (0.1)	
	Han	73 (0.18)	
	Hawaiian or Pacific Islander	17 (0.04)	
	Hispanic or Latino	236 (0.58)	
	Indigenous Australian	795 (1.96)	
	Iranian	95 (0.23)	
	Maori	5 (0.01)	
	Mexican	16,447 (40.59)	
	Native American	6 (0.01)	
	Non-Aboriginal and Torres Strait Islander	100 (0.25)	
	Non-Dutch	12 (0.03)	
	Non-Hispanic or Latino	239 (0.59)	
	White	5235 (12.92)	
	Minority group	1731 (4.27)	
	Missing	10,511 (25.94)	
	Mixed or >1	171 (0.42)	
	Other	1478 (3.65)	
**Clinical characteristics in 160 studies** **, n (%)**			
	Depression	87 (54.4)	
	Depression and anxiety	35 (21.9)	
	Major depressive disorder	21 (13.1)	
	Perinatal depression	11 (6.9)	
	Depression and stress	7 (4.4)	
	Depression in older adults and depression with suicidality	3 (1.9)	
	Not applicable	1 (0.6)	
**Severity, in 160 studies n (%)**			
	Moderate and above	42 (26.2)	
	Mild and above	35 (21.9)	
	Mild to moderate	31 (19.4)	
	Severe	20 (12.5)	
	Moderate	14 (8.8)	
	Not reported	9 (5.6)	
	Mild	5 (3.1)	
	Not applicable	4 (2.5)	

^a^Race and ethnicity in the included studies: not reported, n=84 (52.5%); reported, n=76 (47.5%).

##### Age

Age data were analyzed in relation to means and age ranges (where stated). Most studies (138/160, 86.2%) provided mean age data, with an average of 38.52 (SD 10.87) years. Moreover, 45.6% (73/160) of the studies provided a minimum age, and 41.2% (66/160) of the studies provided a maximum age, with an average age range of 38.52 years. In terms of specific age groupings (18-24, 25-39, 40-64, and >65 years), 15.6% (25/160) studies spanned all 4 categories, and 41.2% (66/160) studies included >1 category.

##### Gender

Of the 160 studies (participants, n=70,720) reviewed, 51,677 (73.07%) of the participants were women , 16,779 (23.73%) were men , and 2147 (3.04%) participants did not report their gender. The diversity of gender generated 7 different categories, including transgender (n=18, 0.03%), queer (n=9, 0.01%), nonconforming (n=9, 0.01%), variant (n=5, 0.007%), gender expansive (n=2, 0.002%), gender fluid (n=1, 0.001%), and nonbinary (n=1, 0.001%). A total of 72 (0.1%) participants did not specify their gender. Most of the studies (145/160, 90.1%) had a mixture of men and women in their sample. However, 14 (8.8%) studies recruited women only while, 1 (0.6%) study men only; 8 (5%) studies recruited more men than women. While 4 (2.5%) studies had an equal number of men and women participants in the sample, 2 (1.2%) studies did not report gender characteristics.

##### Race and Ethnicity

Of the 160 studies (participants, n=70,720), 84 (52.5%) did not report race and ethnic data, leaving 30,197 (42.7%) participants not accounted for. In the remaining studies, there was no information about the race and ethnicity of an additional 10,511 (14.86%) participants, which brings the overall number of participants who did not report their race and ethnicity to 40,708 (57.56%). A total of 76 (47.5%) studies considered race and ethnicity in their analysis involving 40,523 (57.3%) participants and groups such as Asian (n=713, 1.76%; Asian American, Asian, Chinese, New Zealand Indian, Iranian, Han, and Middle Eastern); Black (n=741, 1.83%; Black African American, African American, African, African Canadian, and Black); Hispanic (n=16,684, 41.17%; Mexican, Hispanic Latino, and Latin American); Indigenous (n=851, 2.1%; First Nations or Metis, American Indian or Alaska Native, Maori, Indigenous Australians, Native Americans, Hawaiian Pacific Islander, and Indigenous); White (n=7291, 17.99%; White, Caucasian, European, European New Zealander, Dutch, and American); other (n=3560, 8.79%; non-Hispanic or Latino, non-Dutch, Other, non-Aboriginal Torres Strait Islander, and minority groups); and mixed (n=172, 0.42%; Black and White, mixed or >1).

#### Clinical Characteristics and Severity

There was a spread in terms of the severity of depression among the 160 studies, with 36 (22.5%) studies focusing on mild and above, 31 (19.4%) studies focusing on mild to moderate, and 27 (16.9%) on moderate to severe depression. A smaller number of studies (n=20, 12.5%) focused on severe depression only. Some studies (n=21, 13.1%) included major depressive disorder, perinatal depression (n=12, 7.5%), and (n=3, 1.9%) depression in older adults. A group of studies (n=35, 21.9%) focused on both depression and anxiety, and 3 (1.9%) studies included multiple categories. There were also studies (n=9, 5.6%) that discussed other concerns such as suicidal ideation or alcohol misuse; diverse populations such as lesbian, gay, bisexual, and transgender communities; and different contexts such as workplace or rural communities.

### Research Question 1: Mapping the Types of Digital Interventions

MoodGYM was the most popular named digital intervention (15/160, 9.4%), which used an interactive workbook (Table 4). Beating the Blues was also common (8/160, 5%) and involved an 8-session course supported by a counselor. Other interventions included Good Days Ahead (4/160, 2.5%), a 9-lesson psychoeducational program, and Mindful Mood Balance (3/160, 1.9%), an 8-week course integrating mindfulness meditation with CBT. Furthermore, 5% (8/160) studies used a combination of digital interventions. Some interventions involved apps; for example, Project EVO (3/160, 1.9%) was a video game training app.

**Table 4:** Frequency of named interventions (n=70).

Named intervention	Frequency, n (%)
MoodGYM^a^	15 (21)
Beating the Blues^b^	8 (11)
Good Days Ahead	4 (6)
GET.ON Mood Enhancer, Health tips^c^, iPST^c^ Meru health program, Mindful Mood Balance, Project EVO^c^	3 (4)
BluePages^d^, Cognitive Bias Modification Imagery, Deprexis, Intellicare, Living to the Full, Mom Mood Booster, My Compass^e^, My Strength Inc, The Journal, This Way Up	2 (3)
Ascent, BAML, BetterHelp, Bluewatch, Colour Your Life, Cooperation After Divorce, Dario Behavioural Health, e-SMART, Empower@Home, Feel Stress Free, Feeling Better, Get Happy, Good Life Compass, Gratitude Visit^f^, Happy@Work, HappyMom, Hdep, iFightDepression, Just In Time Adaptive Intervention, LifeApp’tite, Life Flex, Making the Golden Years Golden Again, MamaKits, Man Central, MARIGOLD, Master your Mood, MasterMind, Mindbalance, Mindfulness Virtual Community, MindWise 2.0, Moodbuster, MoodHacker, Mood Manager, MoodGarden^g^, Motherly, NexJ Health Inc, OctaVis, Online Life Review, Online Writing, Op Koers Online, OPTT, Overcoming Thoughts, PaarBalance, Pacifica, Peak, PRIME-D, Signature Strength^f^, Sinasprite, Sokoon, SPARX, Talkspace, TAPI, Three Good Things, Three Funny Things^f^, Thrive, Todac, VIDA, Wellness Workshop	1 (1)

^a^Used in combination with other named interventions (n=4).

^b^Used in combination with MoodGYM (n=1).

^c^Project EVO, iPST, and Health tips used in combination (n=3).

^d^Used in combination with MoodGYM (n=2).

^e^Used in combination with MoodGYM (n=1).

^f^Gratitude visit, Three Good Things, Three Funny Things, and signature strength used in combination (n=1).

^g^Used in combination with MoodGYM (n=1).

Of the 160 studies reviewed, most interventions (n=132, 82.5%) used CBT as their framework, 6 (3.8%) referred to positive psychology interventions, and only 1 (0.6%) adopted a psychoanalytic and psychodynamic approach. In 8 (5%) studies, the interventions were based on an integrative framework such as combining CBT with positive psychology, while 13 (8.1%) studies described interventions in which the theoretical models were either not clearly defined or did not fall within the 4 main approaches to psychotherapy (ie, cognitive-behavioral, humanistic, psychoanalytic and psychodynamic, and integrative). Table 5 shows the definitions of specific types of interventions.

Out of the 160 studies reviewed, 93 (58.1%) were web based, 25 (15.6%) used mobile apps, 13 (8.1%) used computer programs, 1 (0.6%) involved virtual reality, and 1 (0.6%) was avatar based. Moreover, 26 (16.2%) studies used a combination of the above methods.

The interventions included messaging, emails, and calls (17/160, 10.6%), online peer support (11/160, 6.9%), online face-to-face contact (7/160, 4.4%), and virtual or augmented reality (1/160, 0.6%). Interventions were also delivered via participants watching videos (19/160, 11.9%) and listening to audio (11/160, 11.9%).

In terms of psychotherapeutic approaches, the digital interventions were mainly based on CBT (132/160, 82.5%). A small number of interventions used integrative approach (8/160, 5%), positive psychology (5/160, 3.1%) and psychodynamic and psychoanalytic psychotherapy (1/160, 0.6%).

**Table 5:** Definitions of the types of interventions (n=160).

Type of intervention	Definition	Studies, n (%)
No human contact and stand-alone	No human involvement in the therapeutic process, for example, an app that was fully automated and sent reminders to users via notifications or emails, but the participants did not have any contact with a human therapist	58 (36.2)
Human contact	Participants had web-based or offline sessions with the therapist. The contact could be synchronous (eg, a Zoom video call) or asynchronous (eg, an email), but the therapist was involved in the clients’ journey. For example, the contact could include live Zoom video calls only or Zoom video calls and then text follow-up with the therapist. However, the contact had to be with a therapist, not a researcher	11 (6.9)
Blended	Both “stand-alone” and “human contact” interventions were used. For example, the participant worked through modules independently on a website and then met with a therapist via a Zoom video call or in person. The degree of contact with the therapist varied	91 (56.9)

Most of the therapeutic activities (86/160, 53.8%) involved web-based psychoeducation drawn primarily from CBT, including cognitive restructuring (34/160, 21.3%), behavioral activation or activity planning (23/160, 14.8%), mood rating (14/160, 8.8%), problem-solving (16/160, 10%), goal setting (5/160, 3.1%), and graded exposure or behavioral experiments (4/160, 2.5%). Some interventions (22/160, 13.8%) were delivered using creative means such as games or quizzes, music, journal writing, comic books, stories, animations, singing, bibliotherapy, and graphics. Homework with therapeutic tasks also featured (19/160, 11.9%). Different forms of relaxation (17/160, 10.6%) including visualization, guided relaxation, body scan, yoga, self-hypnosis, deep breathing, and progressive muscular relaxation were mentioned. Mindfulness and meditation (16/160, 10%) and acceptance and commitment therapy or values–based interventions (7/160, 4.4%) were also common. Less-common approaches included working with emotions or emotion regulation strategies (5/160, 3.1%), social skills learning (5/160, 3.1%, relapse prevention (2/160, 1.2%), coping skills training (2/160, 1.2%), cognitive training for memory improvement (1/160, 0.6%), neuroplasticity principles (1/160, 0.6%), and an emotional faces memory task (1/160, 0.6%).

Where reported, the most common dosage for interventions was 6 to 12 weeks in length (73/160, 45.6%), 6 to 12 sessions (44/160, 27.5%), and 6 to 12 modules (30/160, 18.6%). A further 23 (14.4%) studies lasted for <6 weeks, while 6 (3.6%) took >12 weeks. Most commonly, the interventions included 8 (mean 9.66) sessions of 60 (mean 43.35) minutes and were spread over 8 (mean 8.29) weeks, taking place once a week. However, due to variation, the average frequency per week was 2.18 sessions. At least 17 (10.6%) studies stated access as “ad libitum” or “self-paced.”

### Research Question 2: Clients’ and Practitioners’ Perspectives on Helpful and Unhelpful Factors Identified in Qualitative and Mixed Methods Studies

#### Overview

Qualitative (13/160, 8.1%) and mixed methods (15/160, 9.4%) studies were used to identify clients’ and practitioners’ perspectives on helpful and unhelpful factors in digital psychotherapeutic interventions for depression. In line with the categorization above, these interventions were divided into 3 groups: with no human contact or stand-alone, with human contact (via digital means), and a combination of human contact with stand-alone approach (blended).

In order to identify the specific helpful and unhelpful factors within each of the above groups, 4 authors used a modified behavior change model [33] as applied by Liverpool et al [34], outlining the barriers and facilitators to engagement in digital mental health interventions including person-specific and intervention-specific influencing factors. In terms of person-specific factors, motivation, opportunity, and capability were listed as the influencing factors. In terms of intervention-specific influencing factors, suitability, usability, and acceptability were identified.

#### Interventions That Did Not Involve Human Contact or Stand-Alone Interventions

The interventions included in this category involved 11fully automated programs. Of these 11 interventions, 10 (91%) were delivered through dedicated websites [40-49] and 1 (9%) was delivered through a fully automated app [50].

A total of 9 (82%) studies discussed helpful and unhelpful factors from the clients’ perspectives. In addition, 1 (9%) study [46] was interested in consulting the public, as potential users, to better understand how interventions for depression could be improved for lesbian and gay users. Another study [50] involved collecting data from potential users and 2 groups of experts formed by researchers and health care professionals.

For the interventions delivered via websites, the most frequently reported (6/10, 60% studies) helpful factors related to “motivation.” Examples of what “motivated” people to engage included gaining improvement in mental health, learning coping skills in difficult situations (eg, when anxious), achieving behavioral change, gaining awareness and insight, learning self-reflection skills, and having a sense of achievement and self-efficacy [44,49]. According to participants in the reviewed studies, digital psychotherapies also provided an “opportunity” to engage in online approaches, which was seen as equally valid as seeing a professional [47]. Reference was also made to the “suitability” of the interventions, as it allowed clients to undertake therapy at their own pace, time, and place [41], and “acceptability,” when participants liked working in private due to discomfort related to working with personal issues [49].

Unhelpful factors related to the interventions delivered via website included issues with “acceptability,” involving comments that the exercises were overwhelming, disconnected from experiences, “typical” advice [48], and repetitive. Participants also commented that they would have preferred a more engaging interactive format [49]. Unhelpful factors also related to the limitations of “opportunity,” as the intervention was too flexible and consequently easy to avoid or too difficult to sustain as it required personal initiative. Users also reported the tasks were too demanding and felt like “work,” which decreased their inclination to engage [42].

The study that focused on a fully automated app [50] discussed “acceptability,” including an appealing visual layout and organization of content of the app, as well as the offer of a wide range of psychological strategies. End users also reported satisfaction with the increased self-awareness promoted by the app, which kept them “motivated” to continue engaging with the intervention.

Some unhelpful factors included the lack of contact with the therapist, which made the therapy “unacceptable” and ineffective [42], and a lack of guidance, making it difficult for participants to know whether they had used the content appropriately [41] or wasted their “opportunity.”

#### Interventions That Involved Human Contact

There were 4 qualitative or mixed methods studies that focused on interventions involving human contact; 2 (50%) studies used a website [40,51], 1 (25%) study used a combination of digital formats (eg, videoconferencing and self-help materials) [52], and 1 (25%) study used videoconferencing as a primary mode of delivery [53]. Helpful and unhelpful factors were reported from the clients’ perspectives.

In terms of websites, helpful factors focused mainly on “usability,” such as explanations that helped with engagement and managing expectations [51]. Improved symptoms of anxiety and social support increased the “motivation” to continue using the intervention [40]. However, intervention “usability” problems were also mentioned as an unhelpful factor. For example, some clients indicated that when there were no attempts to manage the expectations of tasks, this led to difficulties and uncertainty how to respond [51]. No significant change in depression or dysfunctional thinking led to decreased “motivation” for engagement in therapy; this was also seen as an unhelpful factor [40].

When videoconferencing was used, participants reported feeling satisfied, as they were able to engage in therapy from their own environment and adapt it to their own needs (acceptability) [53]. However, they appreciated having an in-person assessment before the digital intervention. Initially, participants were worried that they would feel separated from the therapist, but they did not experience this during the intervention. In terms of unhelpful factors, participants reported “usability” issues especially when there were no technical explanations, and their privacy was compromised. In 1 study that implemented a combination of virtual reality and email [52], the intervention seemed “acceptable,” as the self-help materials provided a calming and structured way to reflect on difficulties without a therapist’s input [52]. Unhelpful aspects related to “usability” issues and uncertainty about the function of the group (where the intervention included online peer support) [52].

In addition, 1 study [54] highlighted the need for collaborative platforms in old age as older participants (aged > 65 years) used media less often than younger participants.

#### Interventions That Combined Human Contact With Automated Interventions (Blended)

Qualitative and mixed methods studies within the “blended” category (20/28, 71%) used mainly human contact alongside noncontact automated interventions.

Human contact methods included face-to-face appointments, telephone or video calls, emails, or asynchronous messaging (eg, via a forum). The most common approach was to use a combination of ≥2 different contact methods (8/28, 29%). In terms of the automated intervention component, 39% (11/28) studies used a website.

In these blended interventions, the use of daily practices, reminders, and likable content were viewed as “helpful” since they “motivated” clients to engage with the intervention [222]. Similarly, learning skills that focused on new ways of relating to negative thoughts, emotions, and sensations [55] and using likable content and helpful activities [56] “motivated” clients to engage. Other “motivating” factors included resources and tools to help manage stress and anxiety [57]; tools for soothing and helping to improve mood [58]; insights and resources offered by a coach [57]; and interventions interrupting the downward spiral of negative thinking [59], increasing the awareness of personal warning signs of impending relapse [222]; and confrontations by therapists in relation to completing more online sessions [60].

Other examples of helpful factors included the “opportunity” to access additional sources of social support, which made some clients feel more connected and less lonely [56,58]. Examples of “suitability,” from the therapists’ perspective, included the freedom to work anywhere at any time [61]. Clients, too, favored flexible scheduling [62]. Blended approaches were also viewed as addressing the issue of inadequate access to specialist care [63].

Examples of unhelpful factors included comments that the modules were not engaging and too lengthy and that certain program features were complicated and hard to follow; these issues had an impact on “usability” [62,64,65]. Other examples of unhelpful factors included an interface that felt less “acceptable”; some clients commented that the programs were not advanced enough [66], too structured [60], and not user-friendly [56]. In some cases, clients felt that they were asked to write “essays,” which was not useful [59]. Clients also commented on the costs related to data charges [58], time and scheduling issues [222], and technical difficulties with access [65], which challenged the “suitability” of the interventions. From practitioners’ perspectives, the constraints of hectic practice, inadequate knowledge, and competing tasks made it more difficult to use [63].

A lack of human contact, real-time interaction, dialogue, and guidance left users feeling a responsibility that required too much from them and sometimes left them feeling lonely [58,61]. The absence of synchronous group interaction led to feeling a loss of interpersonal learning [222], which again seemed like a lost “opportunity” of meaningful therapeutic interaction.

### Research Question 3: Effectiveness of the Interventions

#### Overall Efficacy of Digital Psychotherapies on Depression

**Figure 2:**
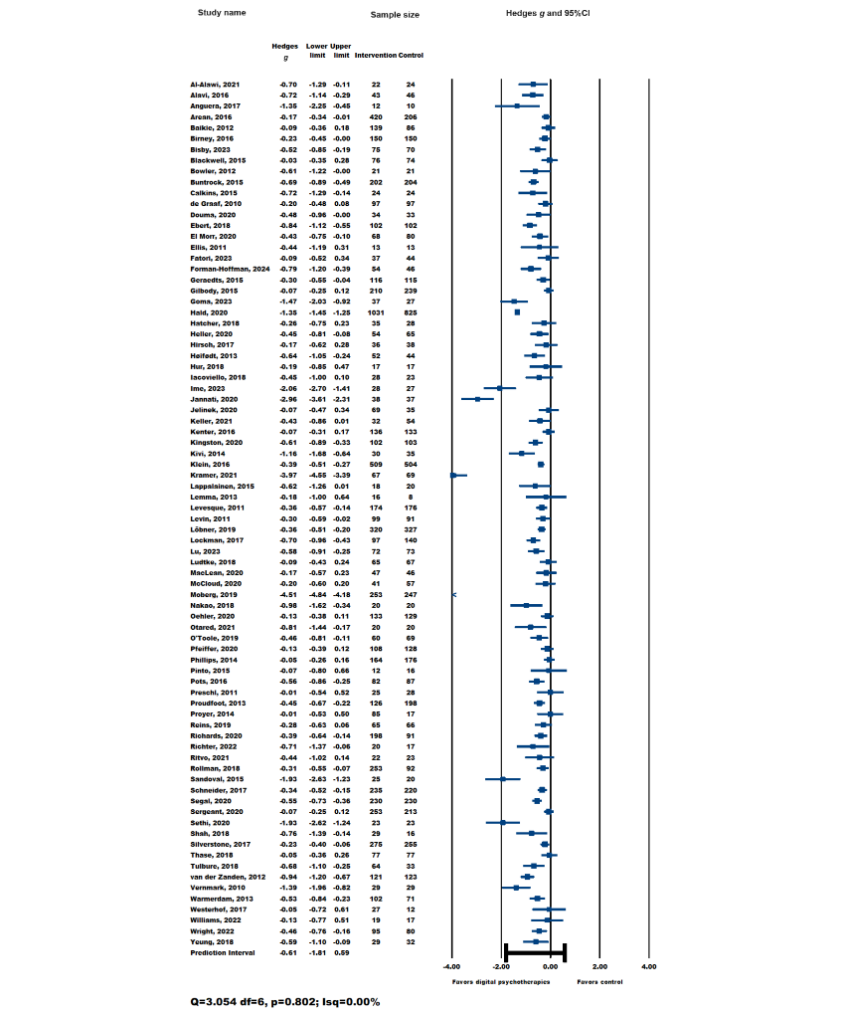
Overall efficacy of digital psychotherapies on depression outcomes in adults.

**Figure 3:**
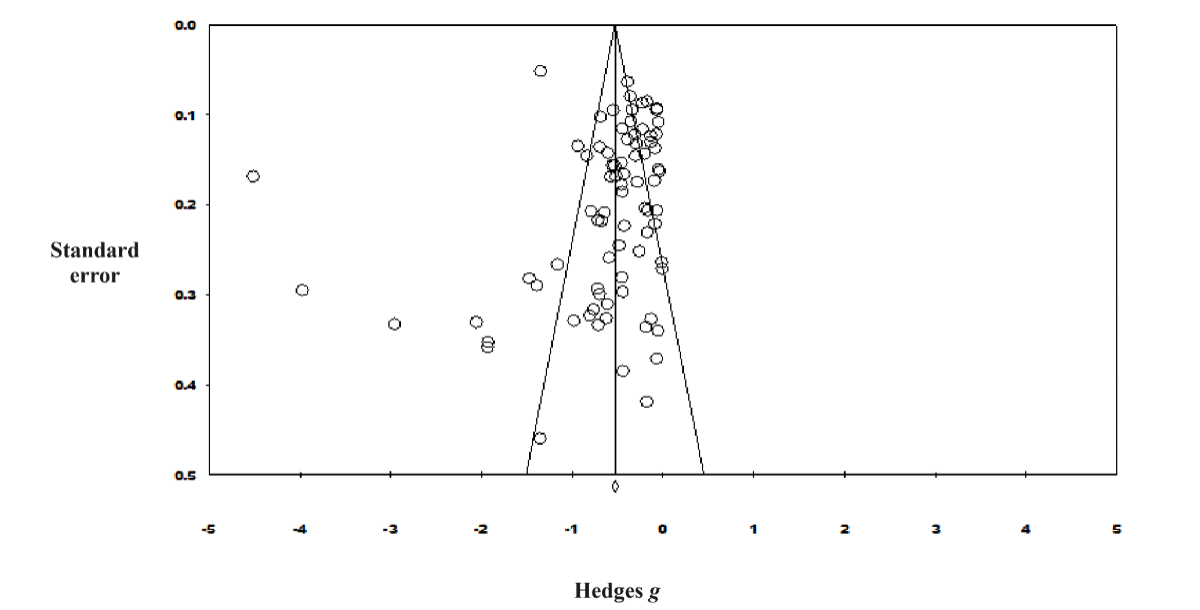
Funnel plot for overall effects without removal of outliers.

A total of 80 studies (intervention group, n=8444; control group, n=7628; total n=16,072) were included in the meta-analysis to examine the effects of digital psychotherapy interventions plus standard care compared to control groups (standard care alone, waiting list, or active controls) for depression in adult participants. The overall effect of digital psychotherapies on depression was moderate to large and statistically significant in favor of digital psychotherapies (Hedges *g*=–0.61, 95% CI –0.75 to –0.47; *Z*=–8.58; *P*<.001) immediately after the intervention (Figure 2 [40,52,59,65,68-80,82,87,89,91,93,94, 101,106,109,113,114,116-136,138-146,149-152,156-158,161-166, 168,170-174,177,178,181,189,191,195,198,201,206,212,213,216, 218,223,224]). The resulting funnel plot from the overall depression outcomes (Figure 3) did not appear to have significant asymmetry (Egger intercept=0.07; *P*=.94).

**Figure 4:**
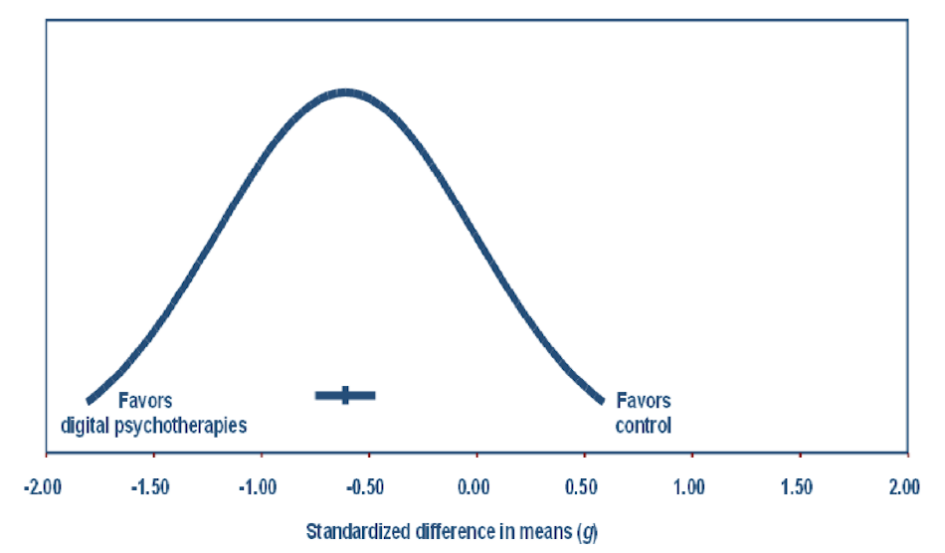
Distribution of true effects of the overall efficacy of digital psychotherapies for depression in adults. The mean effect size is –0.61 (95% CI–0.75 to –0.47). The true effect size is 95% of all comparable populations falls in the interval -1.81 to 0.59.

The *Q* test revealed a value of 1348.221 with 79 degrees of freedom and *P*<.001. Using a criterion α of .100, the null hypothesis that the true effect size is the same in all these studies was rejected. The *I*^2^ statistic indicates that 94% of the variance in observed effects reflects the variance in true effects rather than a sampling error. As shown in Figure 4, assuming that the true effects are normally distributed (in *g* units), it was estimated that the prediction interval falls within –1.811 to 0.587, indicating that the true effect size in 95% of all comparable populations falls in this interval.

**Figure 5:**
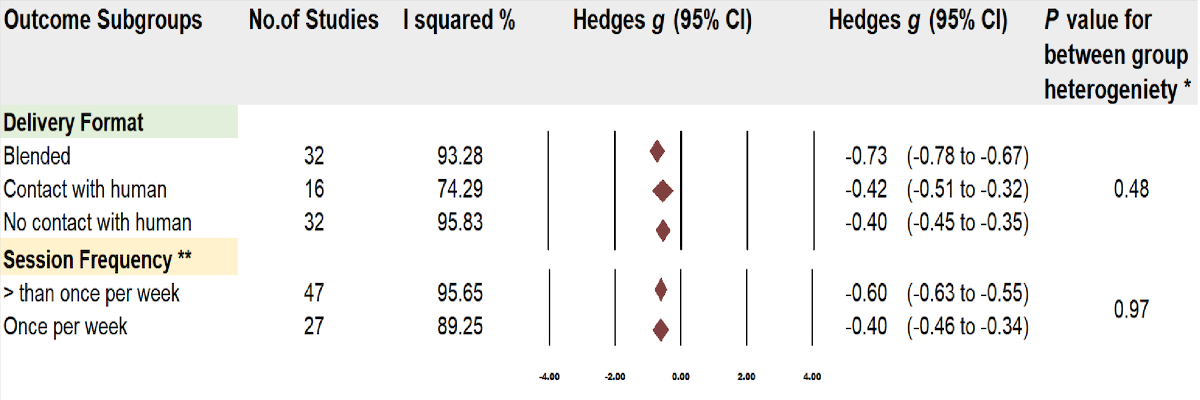
Subgroup analyses. *Q-test for between-group heterogeneity, mixed-effects model. **Six studies that did not offer session frequency data or did not fit into these two categories were excluded from this analysis. .

To explore the potential sources of heterogeneity, subgroup analyses (Figure 5; delivery format: blended [40,59,69,71, 75-78,80,94,106,109,113,116,120,123,130,132,136,138,163,178, 182,186,187,195,198,201,212,213,216,218], contact with human [52,65,68,82,118,121,127,144,157,161,167,170,174,175, 188,223], no human contact [67,70,72-74,79,87,89,91,93,101, 122,125,126,128,134,139,141-143,145,149,151,164,165, 171,177,181,191,206,224], session frequency: more than one per week [40,52,59,69,71,74-77,79,80,87,89,91,93, 101,106,113,120,121,123,125,128,130,132,134,138,139,144, 145,151,157,164,165,168,170,172,174,181,182, 186,188,195,201,213, 216], once per week [65,67,68,70, 72,73,78,82,94,116,118,122,136,141-143,161,163, 171,175,177,178, 187,191,212,223,224]) were conducted based on the intervention characteristics of the studies using a sufficient number of trials (80 for deliver format; 74 for session frequency) and participants. Therefore, the covariate distribution was not concerning in the subgroups. The type of delivery of the intervention (eg, blended, contact, or no contact with humans) did not have statistically significant modifying effects on the results of digital psychotherapies in comparison with the control group (*P*=.48). The intervention effects however are consistently in favor of digital psychotherapies for trials delivered in the 3 different formats studied, although the intervention effect is slightly greater for the trials delivered through a blended format (Hedges *g*=–0.73) than for the trials using contact with human (Hedges *g*=–0.42) or no contact with humans (Hedges *g*=–0.40). Similarly, the frequency of the intervention (once per week or more than once per week) did not have statistically significant modifying effects on the results of digital psychotherapies in comparison with the control group (*P*=.97). However, the intervention effect is slightly greater for trials that delivered the intervention more than once per week (Hedges *g*=–0.60) than for trials offering once per week intervention (Hedges *g*=–0.40). As the residual unexplained heterogeneity between the trials within all these subgroups is still persistent, the validity of the intervention effect is uncertain and requires further exploration to discuss the potential confounding variables.

#### Quality Appraisal

Methodological quality varied across the included studies (Multimedia Appendix 3).

Most of the qualitative studies (12/13, 92%) were assessed as meeting all 5 MMAT criteria [31] (marked as “yes”), 2 (15%) studies were judged as not using appropriate qualitative approaches to answer the research question, and the findings did not seem adequately derived from the data (judged as “no” and “can’t tell/unclear”). In 2 (15%) studies, the interpretation of] the results was judged as not being sufficiently substantiated by data.

Out of 15 nonrandomized quantitative trials (eg, quasi-experimental studies), 1 (7%) study was assessed as meeting all 5 MMAT criteria and 14 (93%) studies were assessed as “no” or “unclear” in terms of meeting at least 1 MMAT criteria, including presenting complete outcome data or appropriately accounting for confounder (n=8, 53%), administration of the intervention (n=7, 47%), having representative target samples (n=5, 33%), and using appropriate measurement tools (n=1, 7%).

Out of 25 quantitative descriptive studies, only 4 (16%) were judged as meeting all 5 criteria on MMAT; 13 (52%) studies were judged as “no” or “unclear” in terms of having representative samples, 5 (20%) for appropriate sampling strategies, and 4 (16%) in relation to measurement tools. A total of 18 (72%) studies were judged as “no” or “unclear” in terms of low risk of nonresponse bias, and 2 (8%) studies were judged as not having appropriate statistical analysis.

Most of the mixed methods studies without an RCT component were judged as not meeting at least 1 MMAT criteria (12/15, 80%). Only 2 (13%) studies met all 5 MMAT criteria, while 2 (13%) studies were judged as “no” or “unclear” for having adequate rationale for using mixed methods design. All studies (15/15, 100%) were judged as having qualitative and quantitative components of the study effectively integrated, but 4 (27%) studies were judged as “no” or “unclear” for providing adequate interpretation of the integration. A total of 10 (67%) studies were judged as “no” or “unclear” for adequately addressing divergences and inconsistencies between the quantitative and qualitative results, and 8 (53%) studies were judged as “no” or “unclear” for adherence to quality criteria for each type of methods.

Regarding the RCTs and mixed methods studies with an RCT component (83 studies) following Higgins and Green’s [30] risk-of-bias assessment tool, random sequence allocation was judged as having a low risk of bias for most of the studies (n=74, 89%). Studies used methods such as software-generated block random sequence [223], algorithm-generated sequence [67], web-based sequence generator [68], computer random number generator [69], manual random sequence generation written in sealed envelopes [70,71], and via independent researchers [72]. A total of 9 (11%) studies, while identified as randomized trials, did not offer sufficient information on how the randomization took place [52,73], and therefore, they were marked as having an “unclear” risk of bias. Many authors did not adequately report their method of concealment, and therefore the risk of bias for this criterion was evaluated as “unclear” for 47% (n=39) of the studies and “high” for 18% (n=15) of the studies. Only 34% (n=29) of the studies clearly reported their method of concealment. When done successfully, authors used blocked randomization delivered in sealed envelopes from a centralized point instead of dividing participants across multiple recruitment centers [23]. Blinding was assessed with respect to participants, personnel, and outcome assessors. We judged only 9% (n=8) of the studies as having implemented blinding successfully with a “low” risk of bias [74,75]. Many studies were not explicit in their reporting of this procedure (“unclear”; n=32, 39%). In some contexts, authors reported that it was difficult or impossible to blind the participants in relation to the type of intervention. In other studies [76,77], blinding of personnel involved in the study was not possible (“high”; n=41, 49%). The risk of bias for blinding of outcome assessment was marked as “unclear” for 38 (46%) studies [78,224] and “high” for 22% (n=18) of the studies. Only 32% (n=27) of the studies were judged as having “low” risk of bias in terms of blinding of outcomes [79,80].

There were some important differences between the outcome assessment measurements across the studies. Blinding of assessors was often not feasible when depression was assessed as a self-reported measure rather than a clinician-rated measure. In many instances, authors reported the reasons for dropout, offered transparent reporting of attrition, and used intention-to-treat analysis (n=74, 89%). This was “unclear” in 10% (n=8) of the studies, and the risk of bias was “high” in only 1% (n=1) of the studies. The risk of selective reporting of the results (including depression outcomes) was judged as “low” for 80% (n=66) of the studies, as the differences within and between intervention groups were given regardless of the direction, magnitude, or statistical significance. The risk of selective reporting was judged as “high” and “unclear” only in 10% (n=8) and 11% (n=9) of the studies, respectively. Overall, selection bias and performance bias were identified as very likely to influence the quality of the results in the included studies.

## Discussion

The systematic review identified the current literature on the topic of digital psychotherapies for adults experiencing depressive symptoms, including the most common types of interventions, the clients’ and practitioners’ views on helpful and unhelpful factors, and the effectiveness of the digital interventions for depression.

### Characteristics of Reviewed Studies

A total of 186 eligible papers (160 primary studies and 26 sibling studies) met the inclusion criteria for this review. The studies accounted for 70,720 participants, including 51,677 (female: 73.07%) participants, which constituted a 3:1 female-to-male participants ratio. When age was reported, a relatively young mean age was present (mean 38.52 years). It therefore appears that women and younger people were overrepresented in the sample. This is reflected in the mental health literature reporting that women are twice as likely to report depression than men [225-227], including postnatal depression. The impacts of social inequalities and gender-based oppression on mental health and depression are well documented by the World Health Organization’s Women’s Mental Health report [228] as well as more recently by other researchers [229-231], highlighting that younger women are particularly susceptible to depression, especially in parts of the world where women struggle with additional burdens linked with unequal division of labor based on gender roles. Other predisposing factors to depression include social roles, cultural norms, and higher vulnerability to violence. Furthermore, women may be attracted to using digital devices, as they can give easier and quicker access as well as more privacy and anonymity. It is possible that digital interventions are particularly attractive to younger women especially in postnatal periods [13], and it might be useful to tailor the developments of these interventions to this group in particular.

The fact that fewer men were included in the review may be closely linked with fixed gender roles. For example, it is possible that, for men, admitting that they have depression is seen as a sign of weakness [232], which may prevent them from accessing psychological help [233]. Studies also show differences between men and women in terms of externalizing and internalizing mental health issues [234]. It seems that men are likely to show depression in an external way, for example, by outbursts of anger [235], smoking [236], physical inactivity [237], or alcohol abuse [238]. For women, depression may be more internalized and linked with social isolation [239] and loneliness [236], and they are more likely to seek psychological help. It is possible that different ways of working with men, and young men in particular, may be needed in psychological therapies for depression by involving more physical or creative approaches to psychological support.

More than a half of the studies (105/160, 65.6%) recruited participants from health care settings or used online methods. In terms of the severity of depressive symptoms, participants had depression as their primary diagnosis with a wide range of severity from mild to severe. It was noticeable, however, that fewer studies focused on severe depression and that these studies tended to have smaller sample sizes. It is possible that because digital interventions are in early stages of development, participants with mild to moderate depression might be more suitable for these studies. It is also possible that digital psychotherapies may be less useful for people with severe depression where face-to-face or more individualized therapy may be more appropriate due to the need for careful monitoring of factors such as suicidal ideation or withdrawal. Köhnen et al [8] reported large heterogeneity of studies with acute depression (eg, due to treatment duration) as well as a higher likelihood of negative events during treatment (eg, deterioration, withdrawal, and dropout), which makes it more difficult to research.

Most studies (133/160, 83%) took place in countries from the global north (ie, the United States, Europe, Australia, and Canada), locations where digital technologies and attention to people’s mental health are prominent. However, the impact of race and ethnicity on mental health was often overlooked. As a result, >50% (84/160) of the studies did not report ethnic data about their participants. These omissions are salient in a context where inequalities in health care have been observable among ethnically diverse groups [240], although efforts have been made to adapt treatments for depression for different cultural groups [241,242]. The lack of collection and reporting of ethnic data in the sampled studies demonstrate that researchers need to engage with current debates on the usefulness, desirability, and feasibility of cross-sectional analysis of mental health issues concerning ethnic data.

Nevertheless, ethnicity data were gathered from 57.3% (40,523/70,720) of the participants included in this systematic review. Hispanic, Latino, or Mexican were the most common group of participants (n=16,684, 41.17%), but most of these participants came from 1 study that was conducted in Mexico [56]. Other studies reported fairly diverse race and ethnicity, including 1.76% (n=713) Asian, 1.83% (n=741) Black, 2.1% (n=851) Indigenous, 17.99% (n=7291) White, 8.79% (n=3560) other, and 0.42% (n=172) mixed. It is possible that the diversity in race and ethnicity in the studies where these data were collected suggests a high need for psychological support among non-White groups [240,243-246]. It is important to note, however, that the prevalence of depression and comorbid psychiatric disorders is not uniform across racial and ethnic groups [247,248]. Ongoing debates question the extent to which existing treatments are effective for diverse ethnic populations [249].

The multiple interpretations of the terms further complicate the report of race and ethnicity data. *Race* and *ethnicity* were sometimes used interchangeably, and *nationality* was used at times as a substitute. The 64 studies incorporating race and ethnicity data in their analysis showed a complex spectrum of classification, as evidenced by >30 reported ethnic groups within what could be considered as homogenous groups. Beyond observable physical characteristics (eg, blackness, brownness, or whiteness), studies referred to finer distinctions such as being African, African American, or African Canadian, which indicate how context can impact the experience of depression in individuals of a seemingly homogenous racial group. Similar findings were observed in the category of indigeneity where studies provided further descriptors such as Native Hawaiian, Pacific Islander, American Indian, Maori, and Aboriginal. The use of subgroups suggests the potential for a detailed examination of how ancestry, culture, and geographical context shape the understandings of depression.

Summaries of the key findings are presented in the subsequent sections in relation to the research questions of the study.

### Research Question 1: What are the Most Common Types of Digital Psychotherapeutic Interventions for Adults With Depression?

The most common types of digital psychotherapeutic interventions were MoodGYM and Beating the Blues, followed by Good Days Ahead, Health Tips, Mindful Mood Balance, and Project EVO. There were several programs with no human contact (58/160, 36.3%), but more than half of the studies (91/160, 56.9%) referred to blended versions. Over half of the programs (n=93, 58.1%) were web based with only a few involving virtual reality and avatars. Some included relaxation and mindful techniques and others included creative means such as games, music, and comic books. Therapeutically, the vast majority (132/160, 82.5%) used CBT as their framing approach delivered in 6 to 12 weeks in a self-paced manner.

### Research Question 2: What Are the Clients’ and Practitioners’ Perceptions on Helpful and Unhelpful Aspects of Digital Psychotherapeutic Interventions for Adults With Depression?

Clients’ and practitioners’ perspectives on helpful and unhelpful factors were examined for interventions that did not involve human contact (automated interventions), those that involved human contact (via digital media), and those that combined human contact with automated interventions (blended).

Non–human contact and automated interventions facilitated motivation and offered an opportunity, which otherwise would not have been available. Engaging in one’s own time, place, and overall pace was also named as a helpful factor. It was, however, easy for participants to feel overwhelmed and disconnected and to believe that they had simply received generic, common-sense advice. The interventions were experienced as too flexible in some cases, making engagement difficult to sustain over time. In other cases, the tasks were experienced as tedious work.

For interventions that involved human contact, for example, via a website, the most helpful factors from a client’s perspective related to usability, which included explanations about tasks. However, “usability” was at times experienced by participants as unhelpful, especially when explanations were not available and there was confusion about how a client should respond. Experience of social support and the perception that their symptoms were improving helped motivate clients to continue using the intervention. However, when they felt no significant changes in their symptoms, clients often became less motivated and even disengaged from the intervention.

For blended approaches, the use of daily practices, reminders, and resources to manage stress and anxiety were seen as motivating. Learning skills to deal with negative thoughts and feelings was also helpful. However, participants commented that they appreciated and felt more attracted to contents that were likable. Blended approaches also provided opportunities to engage with peers (online) and with the therapist; both sets of interactions were seen as helpful. Participants appreciated the flexibility of blended approaches, especially given the difficulty in accessing face-to-face services. The adaptability of blended approaches to patients’ needs and preferences has been noted in literature as different interventions can be easily tailored [250]. However, many unhelpful factors were also identified. These included difficulties with the usability of the interventions (eg, lengthy modules and limited attractiveness) and limited acceptability (not all the digital material made immediate sense; some were too structured or too challenging). In terms of opportunity, participants would have liked face-to-face interactions and synchronous groups, which were not available.

### Research Question 3: What is the Effectiveness of Digital Psychotherapeutic Interventions for Adults With Depression?

The meta-analysis that included 80 studies with 16,072 participants suggested that there is some certainty in the evidence showing moderate to large effects supporting that digital psychotherapies are likely to reduce depression in comparison with control conditions (Hedges *g*=–0.61). Furthermore, subgroup analysis revealed a positive effect of digital psychotherapies to reduce scores of depression across all delivery formats. The blended approach appeared to have the greatest positive effects (Hedges *g*=–0.73 over –0.42 for human contact or –0.40 for no human contact). These findings highlight the benefits of digital interventions. The high impact of the blended approach may be the result of combining the benefits of both human and automated engagement. The value of this type of intervention is also reflected in the range of helpful and unhelpful factors identified in the qualitative and mixed methods studies reviewed and discussed in the *Research Question 2: Clients’ and Practitioners’ Perspectives on Helpful and Unhelpful Factors Identified in Qualitative and Mixed Methods Studies* section. However, we do not know if there is a statistically significant difference between the different digital approaches since they all indicate moderate and high impact. Further investigation on the differences between these approaches will be needed.

It is also worth considering the group of participants targeted by the different interventions. For example, a recent systematic review and meta-analysis of RCTs of smartphone app–based psychological interventions [16] found greater reduction of symptoms in moderate to severe depression than in mild to moderate depression. However, they commented that the findings might be related to the groups of clients that normally do not access mental health services (eg, health care staff during the COVID-19 pandemic). Our systematic review revealed participants with a range of mild to severe symptoms of depression (with less focus on severe depression), and, as highlighted above, it is possible that blended approaches are more relevant for this group of participants. These interesting findings highlight the need to combine the growth of digital interventions with user preferences and personalized care.

Our subgroup analysis also revealed that all forms of delivery, regardless of the frequency and duration of the sessions, were likely to support the reduction of depression in comparison with control groups. However, there was a greater effect size for interventions delivered more often than once per week (Hedges *g*=–0.60 vs –0.40 for interventions delivered once per week), a format of delivery that is more easily provided digitally, minimizing cost and human effort. This challenges the common practice of in-person psychotherapy that tends to be once a week. Although there is evidence that higher session frequency could lead to faster recovery, this is not common practice in in-person psychotherapy. These findings [251] provide useful information that could have a direct impact on future digital developments.

Although we were unable to perform a subgroup analysis for digital psychotherapies with different types of delivery and content due to the diversity of interventions, it is well established that creative tools such as creative writing or use of music as well as yoga or relaxation can impact the levels of engagement [252]. Further exploration is needed in this area to develop digital interventions that do not rely so heavily on CBT and focus on meeting the needs of clients from diverse cultural, socioeconomic, or educational backgrounds [253,254]. An example of this could be Arts for the Blues, a creative therapy for depression that has been offered in a digital format [255].

### Comparison With Other Reviews

The review examines all digital forms of psychotherapy for depression; this is a new contribution to the existing evidence base, creating a cohesive picture. Unlike other reviews that have focused on specific client populations such as those with severe depression [8], chronic health conditions [9-11], and perinatal depression [13] or children and young people [14-17], this review examines digital forms of psychotherapy for all types of depression affecting all populations. Unlike systematic reviews conducted on specific psychotherapeutic approaches such as CBT only [11,18-20] or specific types of delivery (eg, smartphone app [12]), this review included all forms of psychotherapy and gathered information about different types of digital interventions. Given the increase in mental health concerns and depression worldwide, and the growth of digital psychotherapies in recent years, by bringing all populations, approaches, and digital products together in one review, we have been able to acquire a comprehensive picture of the field. This enables more insight and breadth to identify useful interventions as suggested by the recent NICE [3] guidelines.

As per other reviews [256], this review found that the evidence base would benefit from more diverse study participants, higher quality quantitative studies, and more detailed studies concerning which mechanisms within specific interventions lead to a change of outcomes.

### Limitations

The comprehensive nature of the review is certainly one of its major strengths. However, at the same time, depth may have been compromised to accommodate for this “broad stroke” approach. It is possible, for example, that this could be achieved in future studies if qualitative and quantitative studies were reviewed separately.

By contrast, if the aim is to acquire a greater bird’s eye view of this rich and fast-growing field, it is possible that future studies may also consider umbrella reviews (ie, overviews of systematic reviews) including multivariate analysis to obtain a better understanding of how the different reviews relate to each other.

Another limitation relates to the quality of the reviewed studies. Although most qualitative studies (12/13, 92%) were regarded as of “good enough” quality, meeting all MMAT criteria, over a third of the quantitative studies (55/146, 37.6%) had no randomization. In terms of quantitative studies with RCT design (83/146, 56.8%) most studies (80/83, 96%) did not meet all 5 of the MMAT criteria; they were of poor quality and therefore were given less significance in this review.

Despite the risk of bias identified in the RCTs, findings from the meta-analysis and the subgroup analyses offer an invaluable overview of the field, which has not been presented before. The inclusion of 80 studies with 16,072 participants suggests a degree of precision since it is likely that the total sample reaches powered levels (eg, in Grading of Recommendations, Assessment, Development, and Evaluations terms it reached an optimal information size).

### Potential Biases in the Review Process

All aspects of the review were checked by >1 reviewer to minimize possible biases. The initial general data extraction was completed by 4 people. This was followed by data extraction focused on specific characteristics of the studies, which was completed by most of the reviewers. Any issues and discrepancies were discussed in weekly meetings. The reviewers came from different professional backgrounds and research skills. The team comprised psychotherapists and arts therapists, for example, with specialized knowledge in the field and qualitative research skills essential for investigating clients’ and practitioners’ perspectives of helpful and unhelpful factors. It also comprised psychologists and allied health professionals with quantitative research skills, essential for calculating effect sizes.

### Implications for Practice, Policy, and Research

In the current context of a shortage of and increasing demand for mental health interventions, digital approaches hold great promise. However, this review demonstrates that while digital interventions offer flexibility and autonomy for both providers and participants, blended interventions seem important for a positive experience in the treatment of depression and related conditions. Some of the reviewed studies documented limitations with asynchronous interventions and interventions that have no human contact [41,42,48]; these should not be ignored, especially for susceptible and at-risk populations.

Blended approaches delivered more than once a week seem to be particularly useful for the participants, giving the opportunity for a contact with a therapist as well as a platform that can be used between sessions supporting engagement. Digital interventions seem to be helpful for people from diverse ethnic groups and young women in particular. It will be important to tailor and target the digital interventions specifically for this group, and more research is needed in this area.

Most of the reviewed studies focused on CBT, and it seems important to use alternative theoretical approaches for digital psychotherapies, including creative interventions that can accommodate a range of service users’ preferences especially minority populations. In addition, we agree with Fordham et al [257] that new research in the treatment of depression and other disorders ought to shift emphasis from investigating the general effectiveness of interventions to understanding how to achieve a greater effect size for specific populations.

Given the importance of clients’ perceptions of what is helpful in digital psychotherapies, it is paramount that interventions that have high levels of acceptability and usability and prioritize a positive user experience are investigated further.

The fact that this review found that motivation to engage in a treatment was one of the most helpful factors from the clients’ perspective suggests that digital interventions can have a useful auxiliary role to encourage clients to engage in treatments for depression and mitigate against the risk of dropout.

In the context of the COVID-19 pandemic, it is widely accepted that digital approaches show great promise as treatments for mental health problems [258]. However, this review found more research from the perspectives of clients rather than practitioners and therefore, further research is needed to assist practitioners to be aware of the evidence and efficacy of digital interventions they may use in clinical practice [258].

Nondigital interventions could consider the inclusion of a digital option to encourage participants to remain in treatment for depression. Further research could be done to understand the role of digital interventions for enhancing motivation and the mechanisms by which patients can be encouraged to stay in treatment, thus reducing dropout rates.

### Conclusions

The review examines the digital forms of psychotherapy for depression, which is a new contribution to the existing evidence base. Unlike other reviews that have focused on specific client populations and specific psychotherapeutic approaches or modes of delivery, this review included all forms of delivery and gathered information about different types of digital interventions for depression. Given the increase in mental health concerns and depression worldwide and the growth of digital psychotherapies in recent years, by bringing all populations, approaches, and digital products together in 1 review, we have been able to acquire a comprehensive picture of the field, which enables more insights and breadth to identify useful interventions as suggested by the recent NICE guidelines. The review aimed to answer three research questions in relation to digital psychotherapies for depression: (1) the most common types of interventions, (2) the clients’ and practitioners’ perspectives of helpful and unhelpful aspects, and (3) the effectiveness of the interventions. Digital interventions fell into 3 categories including interventions with no human contact and stand-alone interventions, interventions with human contact, and blended including both stand-alone and “human contact” interventions. Blended interventions formed the biggest group of studies. Most of the digital interventions were web based and involved online psychoeducation drawn primarily from CBT delivered once a week. Blended approaches seem to be particularly useful for participants, giving the opportunity for contact with a therapist as well as a platform that can be used between sessions supporting engagement. In terms of the effectiveness of the digital interventions for depression, meta-analysis revealed a moderate to large effect on depression. Analysis of studies with blended approaches revealed the largest effect size in comparison to interventions involving human contact only or no human contact. In addition, the review found that digital interventions seem to be particularly helpful for people from diverse ethnic groups and young women and therefore new research in the treatment of depression ought to shift emphasis from investigating the general effectiveness of interventions to understanding how to achieve a greater effect size for specific populations.

## Appendix

Multimedia Appendix 1 Studies in systematic review.

Multimedia Appendix 2 Characteristics of reviewed studies.

Multimedia Appendix 3 Risk of bias grading and quality appraisal tables.

Multimedia Appendix 4 PRISMA (Preferred Reporting Items for Systematic Reviews and Meta-Analyses) 2020 checklist.

## Abbreviations

CBT: cognitive behavioral therapy
MMAT: Mixed Methods Appraisal Tool
NICE: National Institute for Health and Care Excellence
PICOS: Population, Intervention, Comparison, Outcomes and Study Design
PRISMA: Preferred Reporting Items for Systematic Reviews and Meta-Analyses
RCT: randomized controlled trial
SMD: standardized mean difference
TIDieR: Template for Intervention Description and Replication

## Data Availability

The data sets analyzed during this study are available in Multimedia Appendix 2.

No new data were generated during this study.

## References

[1] (2023). Depressive disorder (depression). World Health Organization.

[2] (2019). Global burden of disease study 2019 (GBD 2019). Global Burden of Disease Collaborative Network.

[3] (2022). Depression in adults: treatment and management. NG 222. National Institute for Health and Care Excellence.

[4] Liverpool S, Moinuddin M, Aithal S, Owen M, Bracegirdle K, Caravotta M, Walker R, Murphy C, Karkou V (2023). Mental health and wellbeing of further and higher education students returning to face-to-face learning after COVID-19 restrictions. PLoS One.

[5] (2020). IAPT guide for delivering treatment remotely during the coronavirus pandemic. National Health Service England and National Health Service Improvements.

[6] (2016). The five year forward view for mental health. The Independent Mental Health Taskforce to the NHS in England. National Health Service England.

[7] (2019). NHS long term plan. National Health Service England.

[8] Köhnen M, Dreier M, Seeralan T, Kriston L, Härter M, Baumeister H, Liebherz S (2021). Evidence on technology-based psychological interventions in diagnosed depression: systematic review. JMIR Ment Health.

[9] White KM, Williamson C, Bergou N, Oetzmann C, de Angel V, Matcham F, Henderson C, Hotopf M (2022). A systematic review of engagement reporting in remote measurement studies for health symptom tracking. NPJ Digit Med.

[10] Paalimäki-Paakki K, Virtanen M, Henner A, Nieminen MT, Kääriäinen M (2022). Effectiveness of digital counseling environments on anxiety, depression, and adherence to treatment among patients who are chronically ill: systematic review. J Med Internet Res.

[11] Lee S, Oh JW, Park KM, Lee S, Lee E (2023). Digital cognitive behavioral therapy for insomnia on depression and anxiety: a systematic review and meta-analysis. NPJ Digit Med.

[12] Serrano-Ripoll MJ, Zamanillo-Campos R, Fiol-DeRoque MA, Castro A, Ricci-Cabello I (2022). Impact of smartphone app-based psychological interventions for reducing depressive symptoms in people with depression: systematic literature review and meta-analysis of randomized controlled trials. JMIR Mhealth Uhealth.

[13] Motrico E, Conejo-Cerón S, Martín-Gómez C, Gómez I, Fonseca A, Moreno-Peral P (2021). Effectiveness of web-based and mobile-based psychological interventions to prevent perinatal depression: study protocol for a systematic review and meta-analysis of randomized controlled trials. Internet Interv.

[14] Ivlev I, Beil TL, Haynes JS, Patnode CD (2022). Rapid evidence review of digital cognitive-behavioral therapy for adolescents with depression. J Adolesc Health.

[15] Wickersham A, Barack T, Cross L, Downs J (2022). Computerized cognitive behavioral therapy for treatment of depression and anxiety in adolescents: systematic review and meta-analysis. J Med Internet Res.

[16] Li SH, Achilles MR, Werner-Seidler A, Beames JR, Subotic-Kerry M, O'Dea B (2022). Appropriate use and operationalization of adherence to digital cognitive behavioral therapy for depression and anxiety in youth: systematic review. JMIR Ment Health.

[17] Martinez K, Menéndez-Menéndez MI, Bustillo A (2021). Awareness, prevention, detection, and therapy applications for depression and anxiety in serious games for children and adolescents: systematic review. JMIR Serious Games.

[18] Chow DY, Jiang X, You JH (2022). Information technology-based versus face-to-face cognitive-behavioural therapy for anxiety and depression: a systematic review and meta-analysis. J Affect Disord.

[19] Kambeitz-Ilankovic L, Rzayeva U, Völkel L, Wenzel J, Weiske J, Jessen F, Reininghaus U, Uhlhaas PJ, Alvarez-Jimenez M, Kambeitz J (2022). A systematic review of digital and face-to-face cognitive behavioral therapy for depression. NPJ Digit Med.

[20] Fadipe MF, Aggarwal S, Johnson C, Beauchamp JE (2023). Effectiveness of online cognitive behavioural therapy on quality of life in adults with depression: a systematic review. J Psychiatr Ment Health Nurs.

[21] Andrews G, Basu A, Cuijpers P, Craske M, McEvoy P, English C, Newby J (2018). Computer therapy for the anxiety and depression disorders is effective, acceptable and practical health care: an updated meta-analysis. J Anxiety Disord.

[22] Renn BN, Hoeft TJ, Lee HS, Bauer AM, Areán PA (2019). Preference for in-person psychotherapy versus digital psychotherapy options for depression: survey of adults in the U.S. NPJ Digit Med.

[23] Berger T (2017). The therapeutic alliance in internet interventions: a narrative review and suggestions for future research. Psychother Res.

[24] Russell GI (2015). Screen Relations: The Limits of Computer-Mediated Psychoanalysis and Psychotherapy.

[25] Martinez-Martin N, Kreitmair K (2018). Ethical issues for direct-to-consumer digital psychotherapy apps: addressing accountability, data protection, and consent. JMIR Ment Health.

[26] Smith K, Moller N, Cooper M, Gabriel L, Roddy J, Sheehy R (2021). Video counselling and psychotherapy: a critical commentary on the evidence base. Couns Psychother Res.

[27] Page MJ, McKenzie JE, Bossuyt PM, Boutron I, Hoffmann TC, Mulrow CD, Shamseer L, Tetzlaff JM, Akl EA, Brennan SE, Chou R, Glanville J, Grimshaw JM, Hróbjartsson A, Lalu MM, Li T, Loder EW, Mayo-Wilson E, McDonald S, McGuinness LA, Stewart LA, Thomas J, Tricco AC, Welch VA, Whiting P, Moher D (2021). The PRISMA 2020 statement: an updated guideline for reporting systematic reviews. BMJ.

[28] Bowling A, Ebrahim S (2005). Handbook of Health Research Methods.

[29] Hoffmann TC, Glasziou PP, Boutron I, Milne R, Perera R, Moher D, Altman DG, Barbour V, Macdonald H, Johnston M, Lamb SE, Dixon-Woods M, McCulloch P, Wyatt JC, Chan A, Michie S (2014). Better reporting of interventions: template for intervention description and replication (TIDieR) checklist and guide. BMJ.

[30] Higgins JP, Green S (2011). Cochrane Handbook for Systematic Reviews of Interventions.

[31] Hong QN, Fàbregues S, Bartlett G, Boardman F, Cargo M, Dagenais P, Gagnon M, Griffiths F, Nicolau B, O’Cathain A, Rousseau M, Vedel I, Pluye P (2018). The Mixed Methods Appraisal Tool (MMAT) version 2018 for information professionals and researchers. Educ Inf.

[32] Popay J, Roberts H, Sowden A (2006). Guidance on the conduct of narrative synthesis in systematic reviews: a product from the ESRC methods programme. Lancaster University.

[33] Michie S, van Stralen MM, West R (2011). The behaviour change wheel: a new method for characterising and designing behaviour change interventions. Implement Sci.

[34] Liverpool S, Mota C, Sales CM, Čuš A, Carletto S, Hancheva C, Sousa S, Cerón SC, Moreno-Peral P, Pietrabissa G, Moltrecht B, Ulberg R, Ferreira N, Edbrooke-Childs J (2020). Engaging children and young people in digital mental health interventions: systematic review of modes of delivery, facilitators, and barriers. J Med Internet Res.

[35] Andrade C (2020). Mean difference, standardized mean difference (SMD), and their use in meta-analysis: as simple as it gets. J Clin Psychiatry.

[36] Higgins JP, Eldridge S, Li T, Higgins J, Thomas J, Chandler J, Cumpston M, Li T, Page MJ (2023). Including variants on randomized trials. Cochrane Handbook for Systematic Reviews of Interventions. Version 6.4.

[37] Egger M, Davey Smith G, Schneider M, Minder C (1997). Bias in meta-analysis detected by a simple, graphical test. BMJ.

[38] Sterne JA, Sutton AJ, Ioannidis JP, Terrin N, Jones DR, Lau J, Carpenter J, Rücker G, Harbord RM, Schmid CH, Tetzlaff J, Deeks JJ, Peters J, Macaskill P, Schwarzer G, Duval S, Altman DG, Moher D, Higgins JP (2011). Recommendations for examining and interpreting funnel plot asymmetry in meta-analyses of randomised controlled trials. BMJ.

[39] Borenstein M, Higgins JP (2013). Meta-analysis and subgroups. Prev Sci.

[40] Ellis LA, Campbell AJ, Sethi S, O'Dea BM (2011). Comparative randomized trial of an online cognitive-behavioral therapy program and an online support group for depression and anxiety. J Cyber Ther Rehabil.

[41] Gerhards SA, Abma TA, Arntz A, de Graaf LE, Evers SM, Huibers MJ, Widdershoven GA (2011). Improving adherence and effectiveness of computerised cognitive behavioural therapy without support for depression: a qualitative study on patient experiences. J Affect Disord.

[42] Knowles SE, Lovell K, Bower P, Gilbody S, Littlewood E, Lester H (2015). Patient experience of computerised therapy for depression in primary care. BMJ Open.

[43] Lucassen MF, Hatcher S, Stasiak K, Fleming T, Shepherd M, Merry SN (2014). The views of lesbian, gay and bisexual youth regarding computerised self-help for depression: an exploratory study. Adv Ment Health.

[44] Richards D, Timulak L (2012). Client-identified helpful and hindering events in therapist-delivered vs. self-administered online cognitive-behavioural treatments for depression in college students. Couns Psychol Q.

[45] Richards D, Timulak L (2013). Satisfaction with therapist-delivered vs. self-administered online cognitive behavioural treatments for depression symptoms in college students. Br J Guid Counc.

[46] Rozbroj T, Lyons A, Pitts M, Mitchell A, Christensen H (2015). Improving self-help e-therapy for depression and anxiety among sexual minorities: an analysis of focus groups with lesbians and gay men. J Med Internet Res.

[47] Schneider J, Sarrami Foroushani P, Grime P, Thornicroft G (2014). Acceptability of online self-help to people with depression: users' views of MoodGYM versus informational websites. J Med Internet Res.

[48] Walsh S, Szymczynska P, Taylor SJ, Priebe S (2018). The acceptability of an online intervention using positive psychology for depression: a qualitative study. Internet Interv.

[49] Shkel J, Green G, Le S, Kaveladze B, Marcotte V, Rushton K, Nguyen T, Schueller SM (2023). Understanding users' experiences of a novel web-based cognitive behavioral therapy platform for depression and anxiety: qualitative interviews from pilot trial participants. JMIR Form Res.

[50] Fuller-Tyszkiewicz M, Richardson B, Klein B, Skouteris H, Christensen H, Austin D, Castle D, Mihalopoulos C, O'Donnell R, Arulkadacham L, Shatte A, Ware A (2018). A mobile app-based intervention for depression: end-user and expert usability testing study. JMIR Ment Health.

[51] Ekberg S, Barnes RK, Kessler DS, Malpass A, Shaw AR (2016). Managing clients' expectations at the outset of online Cognitive Behavioural Therapy (CBT) for depression. Health Expect.

[52] Lemma A, Fonagy P (2013). Feasibility study of a psychodynamic online group intervention for depression. Psychoanal Psychol.

[53] Nogami W, Nakagawa A, Kato N, Sasaki Y, Kishimoto T, Horikoshi M, Mimura M (2022). Efficacy and acceptability of remote cognitive behavioral therapy for patients with major depressive disorder in Japanese clinical settings: a case series. Cogn Behav Pract.

[54] Eichenberg C, Schott M, Sawyer A, Aumayr G, Plößnig M (2018). Feasibility and conceptualization of an e-Mental health treatment for depression in older adults: mixed-methods study. JMIR Aging.

[55] Felder J, Dimidjian S, Beck A, Boggs JM, Segal Z (2014). Mindful mood balance: a case report of Web-based treatment of residual depressive symptoms. Perm J.

[56] Lara MA, Tiburcio M, Aguilar Abrego A, Sánchez-Solís A (2014). A four-year experience with a web-based self-help intervention for depressive symptoms in Mexico. Rev Panam Salud Publica.

[57] Schlosser DA, Campellone TR, Truong B, Anguera JA, Vergani S, Vinogradov S, Arean P (2017). The feasibility, acceptability, and outcomes of PRIME-D: a novel mobile intervention treatment for depression. Depress Anxiety.

[58] Seshu U, Khan HA, Bhardwaj M, Sangeetha C, Aarthi G, John S, Thara R, Raghavan V (2021). A qualitative study on the use of mobile-based intervention for perinatal depression among perinatal mothers in rural Bihar, India. Int J Soc Psychiatry.

[59] Westerhof G, Lamers SM, Postel MG, Bohlmeijer ET (2019). Online therapy for depressive symptoms: an evaluation of counselor-led and peer-supported life review therapy. Gerontologist.

[60] Mol M, Dozeman E, Provoost S, van Schaik A, Riper H, Smit JH (2018). Behind the scenes of online therapeutic feedback in blended therapy for depression: mixed-methods observational study. J Med Internet Res.

[61] Holst A, Nejati S, Björkelund C, Eriksson MC, Hange D, Kivi M, Wikberg C, Petersson E (2017). Patients' experiences of a computerised self-help program for treating depression - a qualitative study of internet mediated cognitive behavioural therapy in primary care. Scand J Prim Health Care.

[62] Danaher BG, Milgrom J, Seeley JR, Stuart S, Schembri C, Tyler MS, Ericksen J, Lester W, Gemmill AW, Lewinsohn P (2012). Web-based intervention for postpartum depression: formative research and design of the MomMoodBooster program. JMIR Res Protoc.

[63] Wilhelmsen M, Høifødt RS, Kolstrup N, Waterloo K, Eisemann M, Chenhall R, Risør MB (2014). Norwegian general practitioners' perspectives on implementation of a guided web-based cognitive behavioral therapy for depression: a qualitative study. J Med Internet Res.

[64] Currie SL, McGrath PJ, Day V (2010). Development and usability of an online CBT program for symptoms of moderate depression, anxiety, and stress in post-secondary students. Comput Human Behav.

[65] Hatcher S, Whittaker R, Patton M, Miles WS, Ralph N, Kercher K, Sharon C (2018). Web-based therapy plus support by a coach in depressed patients referred to secondary mental health care: randomized controlled trial. JMIR Ment Health.

[66] Gega L, Smith J, Reynolds S (2013). Cognitive behaviour therapy (CBT) for depression by computer vs. therapist: patient experiences and therapeutic processes. Psychother Res.

[67] Sergeant S, Mongrain M (2014). An online optimism intervention reduces depression in pessimistic individuals. J Consult Clin Psychol.

[68] Geraedts AS, van Dongen JM, Kleiboer AM, Wiezer NM, van Mechelen W, Cuijpers P, Bosmans JE (2015). Economic evaluation of a web-based guided self-help intervention for employees with depressive symptoms: results of a randomized controlled trial. J Occup Environ Med.

[69] Anguera JA, Gunning FM, Areán PA (2017). Improving late life depression and cognitive control through the use of therapeutic video game technology: a proof-of-concept randomized trial. Depress Anxiety.

[70] Jelinek L, Arlt S, Moritz S, Schröder J, Westermann S, Cludius B (2020). Brief web-based intervention for depression: randomized controlled trial on behavioral activation. J Med Internet Res.

[71] O'Toole MS, Arendt MB, Pedersen CM (2019). Testing an app-assisted treatment for suicide prevention in a randomized controlled trial: effects on suicide risk and depression. Behav Ther.

[72] Keller A, Babl A, Berger T, Schindler L (2021). Efficacy of the web-based PaarBalance program on relationship satisfaction, depression and anxiety - a randomized controlled trial. Internet Interv.

[73] Kingston J, Becker L, Woeginger J, Ellett L (2020). A randomised trial comparing a brief online delivery of mindfulness-plus-values versus values only for symptoms of depression: does baseline severity matter?. J Affect Disord.

[74] Blackwell SE, Browning M, Mathews A, Pictet A, Welch J, Davies J, Watson P, Geddes JR, Holmes EA (2015). Positive imagery-based cognitive bias modification as a web-based treatment tool for depressed adults: a randomized controlled trial. Clin Psychol Sci.

[75] El Morr C, Ritvo P, Ahmad F, Moineddin R, MVC Team (2020). Effectiveness of an 8-week web-based mindfulness virtual community intervention for university students on symptoms of stress, anxiety, and depression: randomized controlled trial. JMIR Ment Health.

[76] Douma M, Maurice-Stam H, Gorter B, Krol Y, Verkleij M, Wiltink L, Scholten L, Grootenhuis MA (2021). Online psychosocial group intervention for parents: positive effects on anxiety and depression. J Pediatr Psychol.

[77] Nakao S, Nakagawa A, Oguchi Y, Mitsuda D, Kato N, Nakagawa Y, Tamura N, Kudo Y, Abe T, Hiyama M, Iwashita S, Ono Y, Mimura M (2018). Web-based cognitive behavioral therapy blended with face-to-face sessions for major depression: randomized controlled trial. J Med Internet Res.

[78] Calkins AW, McMorran KE, Siegle GJ, Otto MW (2014). The effects of computerized cognitive control training on community adults with depressed mood. Behav Cogn Psychother.

[79] Rollman BL, Herbeck Belnap B, Abebe KZ, Spring MB, Rotondi AJ, Rothenberger SD, Karp JF (2018). Effectiveness of online collaborative care for treating mood and anxiety disorders in primary care: a randomized clinical trial. JAMA Psychiatry.

[80] Vernmark K, Lenndin J, Bjärehed J, Carlsson M, Karlsson J, Oberg J, Carlbring P, Eriksson T, Andersson G (2010). Internet administered guided self-help versus individualized e-mail therapy: a randomized trial of two versions of CBT for major depression. Behav Res Ther.

[81] Ahmedani BK, Belville-Robertson T, Hirsch A, Jurayj A (2016). An online mental health and wellness intervention supplementing standard care of depression and anxiety. Arch Psychiatr Nurs.

[82] Alavi N, Hirji A, Sutton C, Naeem F (2016). Online CBT is effective in overcoming cultural and language barriers in patients with depression. J Psychiatr Pract.

[83] Alavi N, Moghimi E, Stephenson C, Gutierrez G, Jagayat J, Kumar A, Shao Y, Miller S, Yee CS, Stefatos A, Gholamzadehmir M, Abbaspour Z, Shirazi A, Gizzarelli T, Khan F, Patel C, Patel A, Yang M, Omrani M (2023). Comparison of online and in-person cognitive behavioral therapy in individuals diagnosed with major depressive disorder: a non-randomized controlled trial. Front Psychiatry.

[84] Andrews B, Klein B, Corboy D, McLaren S, Watson S (2023). Video chat therapist assistance in an adaptive digital intervention for anxiety and depression: reflections from participants and therapists. Prof Psychol Res Pr.

[85] Andrews B, Klein B, van Nguyen H, Corboy D, McLaren S, Watson S (2023). Efficacy of a digital mental health biopsychosocial transdiagnostic intervention with or without therapist assistance for adults with anxiety and depression: adaptive randomized controlled trial. J Med Internet Res.

[86] Anguera JA, Jordan JT, Castaneda D, Gazzaley A, Areán PA (2016). Conducting a fully mobile and randomised clinical trial for depression: access, engagement and expense. BMJ Innov.

[87] Arean PA, Hallgren KA, Jordan JT, Gazzaley A, Atkins DC, Heagerty PJ, Anguera JA (2016). The use and effectiveness of mobile apps for depression: results from a fully remote clinical trial. J Med Internet Res.

[88] Araghi NM, Zarei MA, Saei S, Yousefi Nodeh HR, Mahmoudi E (2022). The effect of online cognitive behavioral therapy on depressive symptoms in recovered patients with COVID-19. J Educ Health Promot.

[89] Baikie KA, Geerligs L, Wilhelm K (2012). Expressive writing and positive writing for participants with mood disorders: an online randomized controlled trial. J Affect Disord.

[90] Bantjes J, Kazdin AE, Cuijpers P, Breet E, Dunn-Coetzee M, Davids c, Stein DJ, Kessler RC (2021). A web-based group cognitive behavioral therapy intervention for symptoms of anxiety and depression among university students: open-label, pragmatic trial. JMIR Ment Health.

[91] Birney AJ, Gunn R, Russell JK, Ary DV (2016). MoodHacker mobile web app with email for adults to self-manage mild-to-moderate depression: randomized controlled trial. JMIR Mhealth Uhealth.

[92] Bowie CR, Gupta M, Holshausen K, Jokic R, Best M, Milev R (2013). Cognitive remediation for treatment-resistant depression: effects on cognition and functioning and the role of online homework. J Nerv Ment Dis.

[93] Bowler JO, Mackintosh B, Dunn BD, Mathews A, Dalgleish T, Hoppitt L (2012). A comparison of cognitive bias modification for interpretation and computerized cognitive behavior therapy: effects on anxiety, depression, attentional control, and interpretive bias. J Consult Clin Psychol.

[94] Buntrock C, Ebert D, Lehr D, Riper H, Smit F, Cuijpers P, Berking M (2015). Effectiveness of a web-based cognitive behavioural intervention for subthreshold depression: pragmatic randomised controlled trial. Psychother Psychosom.

[95] Christensen H, Farrer L, Batterham PJ, Mackinnon A, Griffiths KM, Donker T (2013). The effect of a web-based depression intervention on suicide ideation: secondary outcome from a randomised controlled trial in a helpline. BMJ Open.

[96] Clarke J, Proudfoot J, Birch MR, Whitton AE, Parker G, Manicavasagar V, Harrison V, Christensen H, Hadzi-Pavlovic D (2014). Effects of mental health self-efficacy on outcomes of a mobile phone and web intervention for mild-to-moderate depression, anxiety and stress: secondary analysis of a randomised controlled trial. BMC Psychiatry.

[97] Cluxton-Keller F, Buteau J, Williams M, Stolte P, Monroe-Cassel M, Bruce ML (2019). Engaging rural young mothers in a technology-based intervention for depression. Child Youth Serv.

[98] Collins S, Byrne M, Hawe J, O'Reilly G (2018). Evaluation of a computerized cognitive behavioural therapy programme, MindWise (2.0), for adults with mild-to-moderate depression and anxiety. Br J Clin Psychol.

[99] Danaher BG, Milgrom J, Seeley JR, Stuart S, Schembri C, Tyler MS, Ericksen J, Lester W, Gemmill AW, Kosty DB, Lewinsohn P (2013). MomMoodBooster web-based intervention for postpartum depression: feasibility trial results. J Med Internet Res.

[100] Darnell D, Pullmann MD, Hull TD, Chen S, Areán P (2022). Predictors of disengagement and symptom improvement among adults with depression enrolled in talkspace, a technology-mediated psychotherapy platform: naturalistic observational study. JMIR Form Res.

[101] de Graaf LE, Hollon SD, Huibers MJ (2010). Predicting outcome in computerized cognitive behavioral therapy for depression in primary care: a randomized trial. J Consult Clin Psychol.

[102] de Graaf LE, Gerhards SA, Arntz A, Riper H, Metsemakers JF, Evers SM, Severens JL, Widdershoven G, Huibers MJ (2011). One-year follow-up results of unsupported online computerized cognitive behavioural therapy for depression in primary care: a randomized trial. J Behav Ther Exp Psychiatry.

[103] Dehn Lb, Kater L, Piefke M, Botsch M, Driessen M, Beblo T (2018). Training in a comprehensive everyday-like virtual reality environment compared to computerized cognitive training for patients with depression. Comput Hum Behav.

[104] Dimidjian S, Beck A, Felder JN, Boggs JM, Gallop R, Segal ZV (2014). Web-based mindfulness-based cognitive therapy for reducing residual depressive symptoms: an open trial and quasi-experimental comparison to propensity score matched controls. Behav Res Ther.

[105] Duarte A, Walker S, Littlewood E, Brabyn S, Hewitt C, Gilbody S, Palmer S (2017). Cost-effectiveness of computerized cognitive-behavioural therapy for the treatment of depression in primary care: findings from the Randomised Evaluation of the Effectiveness and Acceptability of Computerised Therapy (REEACT) trial. Psychol Med.

[106] Ebert DD, Buntrock C, Lehr D, Smit F, Riper H, Baumeister H, Cuijpers P, Berking M (2018). Effectiveness of web- and mobile-based treatment of subthreshold depression with adherence-focused guidance: a single-blind randomized controlled trial. Behav Ther.

[107] Eriksson MC, Kivi M, Hange D, Petersson EL, Ariai N, Häggblad P, Ågren H, Spak F, Lindblad U, Johansson B, Björkelund C (2017). Long-term effects of Internet-delivered cognitive behavioral therapy for depression in primary care - the PRIM-NET controlled trial. Scand J Prim Health Care.

[108] Farrer L, Christensen H, Griffiths KM, Mackinnon A (2012). Web-based cognitive behavior therapy for depression with and without telephone tracking in a national helpline: secondary outcomes from a randomized controlled trial. J Med Internet Res.

[109] Fatori D, Zuccolo P, Xavier MO, Matijasevich A, Polanczyk GV (2023). Smartphone-assisted online brief cognitive behavioral therapy to treat maternal depression: findings of a randomized controlled trial. Braz J Psychiatry.

[110] Figueroa CA, DeMasi O, Hernandez-Ramos R, Aguilera A (2021). Who benefits most from adding technology to depression treatment and how? An analysis of engagement with a texting adjunct for psychotherapy. Telemed J E Health.

[111] Fogarty AS, Proudfoot J, Whittle EL, Clarke J, Player MJ, Christensen H, Wilhelm K (2017). Preliminary evaluation of a brief web and mobile phone intervention for men with depression: men's positive coping strategies and associated depression, resilience, and work and social functioning. JMIR Ment Health.

[112] Forman-Hoffman VL, Nelson BW, Ranta K, Nazander A, Hilgert O, de Quevedo J (2021). Significant reduction in depressive symptoms among patients with moderately-severe to severe depressive symptoms after participation in a therapist-supported, evidence-based mobile health program delivered via a smartphone app. Internet Interv.

[113] Forman-Hoffman VL, Sihvonen S, Wielgosz J, Kuhn E, Nelson BW, Peiper NC, Gould CE (2024). Therapist-supported digital mental health intervention for depressive symptoms: a randomized clinical trial. J Affect Disord.

[114] Fundoiano-Hershcovitz Y, Breuer Asher I, Ritholz MD, Feniger E, Manejwala O, Goldstein P (2023). Specifying the efficacy of digital therapeutic tools for depression and anxiety: retrospective, 2-cohort, real-world analysis. J Med Internet Res.

[115] Gerhards SA, de Graaf LE, Jacobs LE, Severens JL, Huibers MJ, Arntz A, Riper H, Widdershoven G, Metsemakers JF, Evers SM (2010). Economic evaluation of online computerised cognitive-behavioural therapy without support for depression in primary care: randomised trial. Br J Psychiatry.

[116] Gilbody S, Littlewood E, Hewitt C, Brierley G, Tharmanathan P, Araya R, Barkham M, Bower P, Cooper C, Gask L, Kessler D, Lester H, Lovell K, Parry G, Richards DA, Andersen P, Brabyn S, Knowles S, Shepherd C, Tallon D, White D (2015). Computerised cognitive behaviour therapy (cCBT) as treatment for depression in primary care (REEACT trial): large scale pragmatic randomised controlled trial. BMJ.

[117] Goldin PR, Lindholm R, Ranta K, Hilgert O, Helteenvuori T, Raevuori A (2019). Feasibility of a therapist-supported, mobile phone-delivered online intervention for depression: longitudinal observational study. JMIR Form Res.

[118] Gomà M, Arias-Pujol E, Prims E, Ferrer J, Lara S, Glover V, Martinez M, Llairó A, Nanzer N (2024). Internet-based interdisciplinary therapeutic group (Grupo Interdisciplinar Online, GIO) for perinatal anxiety and depression-a randomized pilot study during COVID-19. Arch Womens Ment Health.

[119] Gräfe V, Berger T, Hautzinger M, Hohagen F, Lutz W, Meyer B, Moritz S, Rose M, Schröder J, Späth C, Klein JP, Greiner W (2019). Health economic evaluation of a web-based intervention for depression: the EVIDENT-trial, a randomized controlled study. Health Econ Rev.

[120] Hald GM, Ciprić A, Øverup CS, Štulhofer A, Lange T, Sander S, Gad Kjeld S, Strizzi JM (2020). Randomized controlled trial study of the effects of an online divorce platform on anxiety, depression, and somatization. J Fam Psychol.

[121] Heller HM, Hoogendoorn AW, Honig A, Broekman BF, van Straten A (2020). The effectiveness of a guided internet-based tool for the treatment of depression and anxiety in pregnancy (MamaKits online): randomized controlled trial. J Med Internet Res.

[122] Hirsch A, Luellen J, Holder JM, Steinberg G, Dubiel T, Blazejowskyj A, Schladweiler K (2017). Managing depressive symptoms in the workplace using a web-based self-care tool: a pilot randomized controlled trial. JMIR Res Protoc.

[123] Høifødt RS, Lillevoll KR, Griffiths KM, Wilsgaard T, Eisemann M, Waterloo K, Kolstrup N (2013). The clinical effectiveness of web-based cognitive behavioral therapy with face-to-face therapist support for depressed primary care patients: randomized controlled trial. J Med Internet Res.

[124] Hollinghurst S, Peters TJ, Kaur S, Wiles N, Lewis G, Kessler D (2010). Cost-effectiveness of therapist-delivered online cognitive-behavioural therapy for depression: randomised controlled trial. Br J Psychiatry.

[125] Hur jw, Kim B, Park D, Choi SW (2018). A scenario-based cognitive behavioral therapy mobile app to reduce dysfunctional beliefs in individuals with depression: a randomized controlled trial. Telemed J E Health.

[126] Iacoviello BM, Murrough JW, Hoch MM, Huryk KM, Collins KA, Cutter GR, Iosifescu DV, Charney DS (2018). A randomized, controlled pilot trial of the emotional faces memory task: a digital therapeutic for depression. NPJ Digit Med.

[127] İme Y (2023). The effect of online cognitive behavioral group counseling on anxiety, depression, stress and resilience in maraş-centered earthquake survivors. J Ration Emot Cogn Behav Ther.

[128] Jannati N, Mazhari S, Ahmadian L, Mirzaee M (2020). Effectiveness of an app-based cognitive behavioral therapy program for postpartum depression in primary care: a randomized controlled trial. Int J Med Inform.

[129] Jonassaint CR, Gibbs P, Belnap BH, Karp JF, Abebe KK, Rollman BL (2017). Engagement and outcomes for a computerised cognitive-behavioural therapy intervention for anxiety and depression in African Americans. BJPsych Open.

[130] Kenter RM, Cuijpers P, Beekman A, van Straten A (2016). Effectiveness of a web-based guided self-help intervention for outpatients with a depressive disorder: short-term results from a randomized controlled trial. J Med Internet Res.

[131] Kim DR, Hantsoo L, Thase ME, Sammel M, Epperson CN (2014). Computer-assisted cognitive behavioral therapy for pregnant women with major depressive disorder. J Womens Health (Larchmt).

[132] Kivi M, Eriksson MC, Hange D, Petersson EL, Vernmark K, Johansson B, Björkelund C (2014). Internet-based therapy for mild to moderate depression in Swedish primary care: short term results from the PRIM-NET randomized controlled trial. Cogn Behav Ther.

[133] Kladnitski N, Smith J, Allen A, Andrews G, Newby JM (2018). Online mindfulness-enhanced cognitive behavioural therapy for anxiety and depression: outcomes of a pilot trial. Internet Interv.

[134] Klein JP, Berger T, Schröder J, Späth C, Meyer B, Caspar F, Lutz W, Arndt A, Greiner W, Gräfe V, Hautzinger M, Fuhr K, Rose M, Nolte S, Löwe B, Anderssoni G, Vettorazzi E, Moritz S, Hohagen F (2016). Effects of a psychological internet intervention in the treatment of mild to moderate depressive symptoms: results of the EVIDENT study, a randomized controlled trial. Psychother Psychosom.

[135] Lutz W, Arndt A, Rubel J, Berger T, Schröder J, Späth C, Meyer B, Greiner W, Gräfe V, Hautzinger M, Fuhr K, Rose M, Nolte S, Löwe B, Hohagen F, Klein JP, Moritz S (2017). Defining and predicting patterns of early response in a web-based intervention for depression. J Med Internet Res.

[136] Krämer LV, Grünzig SD, Baumeister H, Ebert DD, Bengel J (2021). Effectiveness of a guided web-based intervention to reduce depressive symptoms before outpatient psychotherapy: a pragmatic randomized controlled trial. Psychother Psychosom.

[137] Krusche A, Cyhlarova E, Williams JM (2013). Mindfulness online: an evaluation of the feasibility of a web-based mindfulness course for stress, anxiety and depression. BMJ Open.

[138] Lappalainen P, Langrial S, Oinas-Kukkonen H, Tolvanen A, Lappalainen R (2015). Web-based acceptance and commitment therapy for depressive symptoms with minimal support: a randomized controlled trial. Behav Modif.

[139] Levesque DA, van Marter DF, Schneider RJ, Bauer MR, Goldberg DN, Prochaska JO, Prochaska JM (2011). Randomized trial of a computer-tailored intervention for patients with depression. Am J Health Promot.

[140] Liu J, Duan W, Xiao Z, Wu Y (2024). The effectiveness of online group mindfulness-based cognitive therapy for outpatients with depression in China. J Affect Disord.

[141] Löbner M, Pabst A, Stein J, Dorow M, Matschinger H, Luppa M, Maroß A, Kersting A, König HH, Riedel-Heller SG (2018). Computerized cognitive behavior therapy for patients with mild to moderately severe depression in primary care: A pragmatic cluster randomized controlled trial (@ktiv). J Affect Disord.

[142] Lokman S, Leone SS, Sommers-Spijkerman M, van der Poel A, Smit F, Boon B (2017). Complaint-directed mini-interventions for depressive complaints: a randomized controlled trial of unguided web-based self-help interventions. J Med Internet Res.

[143] Lu Y, Li Y, Huang Y, Zhang X, Wang J, Wu L, Cao F (2023). Effects and mechanisms of a web- and mobile-based acceptance and commitment therapy intervention for anxiety and depression symptoms in nurses: fully decentralized randomized controlled trial. J Med Internet Res.

[144] Lüdtke T, Westermann S, Pult LK, Schneider BC, Pfuhl G, Moritz S (2018). Evaluation of a brief unguided psychological online intervention for depression: a controlled trial including exploratory moderator analyses. Internet Interv.

[145] MacLean S, Corsi DJ, Litchfield S, Kucharski J, Genise K, Selaman Z, Testa V, Hatcher S (2020). Coach-facilitated web-based therapy compared with information about web-based resources in patients referred to secondary mental health care for depression: randomized controlled trial. J Med Internet Res.

[146] Mahoney A, Li I, Haskelberg H, Millard M, Newby JM (2021). The uptake and effectiveness of online cognitive behaviour therapy for symptoms of anxiety and depression during COVID-19. J Affect Disord.

[147] Mahoney A, Shiner CT, Grierson AB, Sharrock MJ, Loughnan SA, Harrison V, Millard M (2023). Online cognitive behaviour therapy for maternal antenatal and postnatal anxiety and depression in routine care. J Affect Disord.

[148] Marcelle ET, Nolting L, Hinshaw SP, Aguilera A (2019). Effectiveness of a multimodal digital psychotherapy platform for adult depression: a naturalistic feasibility study. JMIR Mhealth Uhealth.

[149] McCloud T, Jones R, Lewis G, Bell V, Tsakanikos E (2020). Effectiveness of a mobile app intervention for anxiety and depression symptoms in university students: randomized controlled trial. JMIR Mhealth Uhealth.

[150] McMurchie W, Macleod F, Power K, Laidlaw K, Prentice N (2013). Computerised cognitive behavioural therapy for depression and anxiety with older people: a pilot study to examine patient acceptability and treatment outcome. Int J Geriatr Psychiatry.

[151] Moberg C, Niles A, Beermann D (2019). Guided self-help works: randomized waitlist controlled trial of Pacifica, a mobile app integrating cognitive behavioral therapy and mindfulness for stress, anxiety, and depression. J Med Internet Res.

[152] Moghimi E, Stephenson C, Agarwal A, Nikjoo N, Malakouti N, Layzell G, O'Riordan A, Jagayat J, Shirazi A, Gutierrez G, Khan F, Patel C, Yang M, Omrani M, Alavi N (2023). Efficacy of an electronic coganitive behavioral therapy program delivered via the online psychotherapy tool for depression and anxiety related to the COVID-19 pandemic: pre-post pilot study. JMIR Ment Health.

[153] Morthland M, Shah A, Meadows JT, Scogin F (2020). Development of an audio and computer cognitive behavioral therapy for depression in older adults. Aging Ment Health.

[154] Moskowitz JT, Addington EL, Shiu E, Bassett SM, Schuette S, Kwok I, Freedman ME, Leykin Y, Saslow LR, Cohn MA, Cheung EO (2021). Facilitator contact, discussion boards, and virtual badges as adherence enhancements to a web-based, self-guided, positive psychological intervention for depression: randomized controlled trial. J Med Internet Res.

[155] Motter JN, Grinberg A, Lieberman DH, Iqnaibi WB, Sneed JR (2019). Computerized cognitive training in young adults with depressive symptoms: effects on mood, cognition, and everyday functioning. J Affect Disord.

[156] Nelson Cb, Abraham Km, Walters H, Pfeiffer Pn, Valenstein M (2014). Integration of peer support and computer-based CBT for veterans with depression. Comput Human Behav.

[157] Oehler C, Görges F, Rogalla M, Rummel-Kluge C, Hegerl U (2020). Efficacy of a guided web-based self-management intervention for depression or dysthymia: randomized controlled trial with a 12-month follow-up using an active control condition. J Med Internet Res.

[158] Ofoegbu TO, Asogwa U, Otu MS, Ibenegbu C, Muhammed A, Eze B (2020). Efficacy of guided internet-assisted intervention on depression reduction among educational technology students of Nigerian universities. Medicine (Baltimore).

[159] Openshaw Dk, Pfister R, Silverbaltt H, Moen D (2011). Providing mental health services to women diagnosed with depression in rural Utah communities: using technologically assisted psychotherapeutic intervention as the delivery medium. Rural Ment Health.

[160] Orr LC, Graham AK, Mohr DC, Greene CJ (2020). Engagement and clinical improvement among older adult primary care patients using a mobile intervention for depression and anxiety: case studies. JMIR Ment Health.

[161] Otared N, Moharrampour NG, Vojoudi B, Najafabadi AJ (2021). A group-based online acceptance and commitment therapy treatment for depression, anxiety symptomns and quality of life in healthcare workers during COVID-19. Int J Psicol Psicol Ther.

[162] Pettitt AK, Nelson BW, Forman-Hoffman VL, Goldin PR, Peiper NC (2024). Longitudinal outcomes of a therapist-supported digital mental health intervention for depression and anxiety symptoms: a retrospective cohort study. Psychol Psychother.

[163] Pfeiffer PN, Pope B, Houck M, Benn-Burton W, Zivin K, Ganoczy D, Kim HM, Walters H, Emerson L, Nelson CB, Abraham KM, Valenstein M (2020). Effectiveness of peer-supported computer-based CBT for depression among veterans in primary care. Psychiatr Serv.

[164] Phillips R, Schneider J, Molosankwe I, Leese M, Foroushani PS, Grime P, McCrone P, Morriss R, Thornicroft G (2014). Randomized controlled trial of computerized cognitive behavioural therapy for depressive symptoms: effectiveness and costs of a workplace intervention. Psychol Med.

[165] Pinto MD, Greenblatt AM, Hickman RL, Rice HM, Thomas TM, Clochesy JM (2016). Assessing the critical parameters of eSMART-MH: a promising avatar-based digital therapeutic intervention to reduce depressive symptoms. Perspect Psychiatr Care.

[166] Pinto MD, Hickman RL, Clochesy J, Buchner M (2013). Avatar-based depression self-management technology: promising approach to improve depressive symptoms among young adults. Appl Nurs Res.

[167] Pots WT, Fledderus M, Meulenbeek PA, ten Klooster PM, Schreurs KM, Bohlmeijer ET (2016). Acceptance and commitment therapy as a web-based intervention for depressive symptoms: randomised controlled trial. Br J Psychiatry.

[168] Pots WT, Trompetter HR, Schreurs KM, Bohlmeijer ET (2016). How and for whom does web-based acceptance and commitment therapy work? Mediation and moderation analyses of web-based ACT for depressive symptoms. BMC Psychiatry.

[169] Pratap A, Renn BN, Volponi J, Mooney SD, Gazzaley A, Arean PA, Anguera JA (2018). Using mobile apps to assess and treat depression in Hispanic and Latino populations: fully remote randomized clinical trial. J Med Internet Res.

[170] Preschl B, Maercker A, Wagner B (2011). The working alliance in a randomized controlled trial comparing online with face-to-face cognitive-behavioral therapy for depression. BMC Psychiatry.

[171] Proudfoot J, Clarke J, Birch MR, Whitton AE, Parker G, Manicavasagar V, Harrison V, Christensen H, Hadzi-Pavlovic D (2013). Impact of a mobile phone and web program on symptom and functional outcomes for people with mild-to-moderate depression, anxiety and stress: a randomised controlled trial. BMC Psychiatry.

[172] Proyer RT, Gander F, Wellenzohn S, Ruch W (2014). Positive psychology interventions in people aged 50-79 years: long-term effects of placebo-controlled online interventions on well-being and depression. Aging Ment Health.

[173] Pugh NE, Hadjistavropoulos HD, Fuchs CM (2014). Internet therapy for postpartum depression: a case illustration of emailed therapeutic assistance. Arch Womens Ment Health.

[174] Reins JA, Boß L, Lehr D, Berking M, Ebert DD (2019). The more I got, the less I need? Efficacy of internet-based guided self-help compared to online psychoeducation for major depressive disorder. J Affect Disord.

[175] Richards D, Enrique A, Eilert N, Franklin M, Palacios J, Duffy D, Earley C, Chapman J, Jell G, Sollesse S, Timulak L (2020). A pragmatic randomized waitlist-controlled effectiveness and cost-effectiveness trial of digital interventions for depression and anxiety. NPJ Digit Med.

[176] Richards D, Timulak L, Hevey D (2013). A comparison of two online cognitive‐behavioural interventions for symptoms of depression in a student population: the role of therapist responsiveness. Couns Psychother Res.

[177] Richter LM, Machleit-Ebner A, Scherbaum N, Bonnet U (2023). How effective is a web-based mental health intervention (Deprexis) in the treatment of moderate and major depressive disorders when started during routine psychiatric inpatient treatment as an adjunct therapy? A pragmatic parallel-group randomized controlled trial. Fortschr Neurol Psychiatr.

[178] Ritvo P, Knyahnytska Y, Pirbaglou M, Wang W, Tomlinson G, Zhao H, Linklater R, Bai S, Kirk M, Katz J, Harber L, Daskalakis Z (2021). Online mindfulness-based cognitive behavioral therapy intervention for youth with major depressive disorders: randomized controlled trial. J Med Internet Res.

[179] Rotondi AJ, Belnap BH, Rothenberger S, Feldman R, Hanusa B, Rollman BL (2024). Predictors of use and drop out from a web-based cognitive behavioral therapy program and health community for depression and anxiety in primary care patients: secondary analysis of a randomized controlled trial. JMIR Ment Health.

[180] Sampson E (2023). Implementing digital cognitive-behavioral therapy for major depressive disorder in routine psychiatric appointments: a pilot project in a rural population. J Psychosoc Nurs Ment Health Serv.

[181] Sandoval LR, Buckey JC, Ainslie R, Tombari M, Stone W, Hegel MT (2017). Randomized controlled trial of a computerized interactive media-based problem solving treatment for depression. Behav Ther.

[182] Schneider BC, Schröder J, Berger T, Hohagen F, Meyer B, Späth C, Greiner W, Hautzinger M, Lutz W, Rose M, Vettorazzi E, Moritz S, Klein JP (2018). Corrigendum to ``bridging the "digital divide": a comparison of use and effectiveness of an online intervention for depression between baby boomers and millennials''. J Affect Disord.

[183] Schueller SM, Mohr DC (2015). Initial field trial of a coach-supported web-based depression treatment. Int Conf Pervasive Comput Technol Healthc.

[184] Schuster R, Kalthoff I, Walther A, Köhldorfer L, Partinger E, Berger T, Laireiter AR (2019). Effects, adherence, and therapists' perceptions of web- and mobile-supported group therapy for depression: mixed-methods study. J Med Internet Res.

[185] Schuster R, Sigl S, Berger T, Laireiter AR (2018). Patients' experiences of web- and mobile-assisted group therapy for depression and implications of the group setting: qualitative follow-up study. JMIR Ment Health.

[186] Segal ZV, Dimidjian S, Beck A, Boggs JM, Vanderkruik R, Metcalf CA, Gallop R, Felder JN, Levy J (2020). Outcomes of online mindfulness-based cognitive therapy for patients with residual depressive symptoms: a randomized clinical trial. JAMA Psychiatry.

[187] Sethi S (2020). Treating youth depression and anxiety: a randomised controlled trial examining the efficacy of computerised versus face‐to‐face cognitive behaviour therapy. Aust Psychol.

[188] Shah A, Morthland M, Scogin F, Presnell A, DiNapoli EA, DeCoster J, Yang X (2018). Audio and computer cognitive behavioral therapy for depressive symptoms in older adults: a pilot randomized controlled trial. Behav Ther.

[189] Sharry J, Davidson R, McLoughlin O, Doherty G (2013). A service-based evaluation of a therapist-supported online cognitive behavioral therapy program for depression. J Med Internet Res.

[190] Silva Almodovar A, Surve S, Axon DR, Cooper D, Nahata MC (2018). Self-directed engagement with a mobile app (Sinasprite) and its effects on confidence in coping skills, depression, and anxiety: retrospective longitudinal study. JMIR Mhealth Uhealth.

[191] Silverstone PH, Rittenbach K, Suen VY, Moretzsohn A, Cribben I, Bercov M, Allen A, Pryce C, Hamza DM, Trew M (2017). Depression outcomes in adults attending family practice were not improved by screening, stepped-care, or online CBT during a 12-week study when compared to controls in a randomized trial. Front Psychiatry.

[192] Spates CR, Kalata AH, Ozeki S, Stanton CE, Peters S (2013). Initial open trial of a computerized behavioral activation treatment for depression. Behav Modif.

[193] Stearns-Yoder KA, Ryan AT, Smith AA, Forster JE, Barnes SM, Brenner LA (2022). Computerized cognitive behavioral therapy intervention for depression among veterans: acceptability and feasibility study. JMIR Form Res.

[194] Thase ME, McCrone P, Barrett MS, Eells TD, Wisniewski SR, Balasubramani GK, Brown GK, Wright JH (2020). Improving cost-effectiveness and access to cognitive behavior therapy for depression: providing remote-ready, computer-assisted psychotherapy in times of crisis and beyond. Psychother Psychosom.

[195] Thase ME, Wright JH, Eells TD, Barrett MS, Wisniewski SR, Balasubramani GK, McCrone P, Brown GK (2018). Improving the efficiency of psychotherapy for depression: computer-assisted versus standard CBT. Am J Psychiatry.

[196] Titov N, Dear BF, Staples LG, Bennett-Levy J, Klein B, Rapee RM, Shann C, Richards D, Andersson G, Ritterband L, Purtell C, Bezuidenhout G, Johnston L, Nielssen OB (2015). MindSpot clinic: an accessible, efficient, and effective online treatment service for anxiety and depression. Psychiatr Serv.

[197] Titzler I, Egle V, Berking M, Gumbmann C, Ebert DD (2022). Blended psychotherapy: treatment concept and case report for the integration of internet- and mobile-based interventions into brief psychotherapy of depressive disorders. Verhaltenstherapie.

[198] Tulbure BT, Rusu A, Sava FA, Sălăgean N, Farchione TJ (2018). A web-based transdiagnostic intervention for affective and mood disorders: randomized controlled trial. JMIR Ment Health.

[199] van der Zanden R, Curie K, van Londen M, Kramer J, Steen G, Cuijpers P (2014). Web-based depression treatment: associations of clients' word use with adherence and outcome. J Affect Disord.

[200] van der Zanden R, Galindo-Garre F, Curie K, Kramer J, Cuijpers P (2014). Online cognitive-based intervention for depression: exploring possible circularity in mechanisms of change. Psychol Med.

[201] van der Zanden R, Kramer J, Gerrits R, Cuijpers P (2012). Effectiveness of an online group course for depression in adolescents and young adults: a randomized trial. J Med Internet Res.

[202] Venkatesan A, Rahimi L, Kaur M, Mosunic C (2020). Digital cognitive behavior therapy intervention for depression and anxiety: retrospective study. JMIR Ment Health.

[203] Wagner B, Horn AB, Maercker A (2014). Internet-based versus face-to-face cognitive-behavioral intervention for depression: a randomized controlled non-inferiority trial. J Affect Disord.

[204] Wahle F, Kowatsch T, Fleisch E, Rufer M, Weidt S (2016). Mobile sensing and support for people with depression: a pilot trial in the wild. JMIR Mhealth Uhealth.

[205] Wang L, Miller L (2023). Assessment and disruption of ruminative episodes to enhance mobile cognitive behavioral therapy just-in-time adaptive interventions in clinical depression: pilot randomized controlled trial. JMIR Form Res.

[206] Warmerdam L, van Straten A, Jongsma J, Twisk J, Cuijpers P (2010). Online cognitive behavioral therapy and problem-solving therapy for depressive symptoms: exploring mechanisms of change. J Behav Ther Exp Psychiatry.

[207] Watts S, Mackenzie A, Thomas C, Griskaitis A, Mewton L, Williams A, Andrews G (2013). CBT for depression: a pilot RCT comparing mobile phone vs. computer. BMC Psychiatry.

[208] Welch ES, Weigand A, Hooker JE, Philip NS, Tyrka AR, Press DZ, Carpenter LL (2019). Feasibility of computerized cognitive-behavioral therapy combined with bifrontal transcranial direct current stimulation for treatment of major depression. Neuromodulation.

[209] Whiteside U, Richards J, Steinfeld B, Simon G, Caka S, Tachibana C, Stuckey S, Ludman E (2014). Online cognitive behavioral therapy for depressed primary care patients: a pilot feasibility project. Perm J.

[210] Whitton AE, Proudfoot J, Clarke J, Birch MR, Parker G, Manicavasagar V, Hadzi-Pavlovic D (2015). Breaking open the black box: isolating the most potent features of a web and mobile phone-based intervention for depression, anxiety, and stress. JMIR Ment Health.

[211] Wijnen BF, Lokman S, Leone S, Evers SM, Smit F (2018). Complaint-directed mini-interventions for depressive symptoms: a health economic evaluation of unguided web-based self-help interventions based on a randomized controlled trial. J Med Internet Res.

[212] Williams C, McClay CA, Martinez R, Morrison J, Haig C, Jones R, Farrand P (2022). Online cognitive behavioral therapy (CBT) life skills program for depression: pilot randomized controlled trial. JMIR Form Res.

[213] Wright JH, Owen J, Eells TD, Antle B, Bishop LB, Girdler R, Harris LM, Wright RB, Wells MJ, Gopalraj R, Pendleton ME, Ali S (2022). Effect of computer-assisted cognitive behavior therapy vs usual care on depression among adults in primary care: a randomized clinical trial. JAMA Netw Open.

[214] Wu MS, Wickham RE, Chen SY, Chen C, Lungu A (2021). Examining the impact of digital components across different phases of treatment in a blended care cognitive behavioral therapy intervention for depression and anxiety: pragmatic retrospective study. JMIR Form Res.

[215] Xiang X, Kayser J, Ash S, Zheng C, Sun Y, Weaver A, Dunkle R, Blackburn JA, Halavanau A, Xue J, Himle JA (2023). Web-based cognitive behavioral therapy for depression among homebound older adults: development and usability study. JMIR Aging.

[216] Yeung A, Wang F, Feng F, Zhang J, Cooper A, Hong L, Wang W, Griffiths K, Bennett K, Bennett A, Alpert J, Fava M (2018). Outcomes of an online computerized cognitive behavioral treatment program for treating chinese patients with depression: a pilot study. Asian J Psychiatr.

[217] Zhang R, Nicholas J, Knapp AA, Graham AK, Gray E, Kwasny MJ, Reddy M, Mohr DC (2019). Clinically meaningful use of mental health apps and its effects on depression: mixed methods study. J Med Internet Res.

[218] Bisby MA, Balakumar T, Scott AJ, Titov N, Dear BF (2024). An online therapist-guided ultra-brief treatment for depression and anxiety: a randomized controlled trial. Psychol Med.

[219] Amer NA, Shohieb SM, Eladrosy WM, Elbakry HM, Elrazek SM (2023). Sokoon: a gamification-based cognitive behavioral therapy application – an application for depression, stress, and anxiety. Int J Gaming Comput Mediat Simul.

[220] Mohr DC, Schueller SM, Tomasino KN, Kaiser SM, Alam N, Karr C, Vergara JL, Gray EL, Kwasny MJ, Lattie EG (2019). Comparison of the effects of coaching and receipt of app recommendations on depression, anxiety, and engagement in the IntelliCare platform: factorial randomized controlled trial. J Med Internet Res.

[221] Broglia E, Millings A, Barkham M (2019). Counseling with guided use of a mobile well-being app for students experiencing anxiety or depression: clinical outcomes of a feasibility trial embedded in a student counseling service. JMIR Mhealth Uhealth.

[222] Boggs JM, Beck A, Felder JN, Dimidjian S, Metcalf CA, Segal ZV (2014). Web-based intervention in mindfulness meditation for reducing residual depressive symptoms and relapse prophylaxis: a qualitative study. J Med Internet Res.

[223] Al-Alawi M, McCall RK, Sultan A, Al Balushi N, Al-Mahrouqi T, Al Ghailani A, Al Sabti H, Al-Maniri A, Panchatcharam SM, Al Sinawi H (2021). Efficacy of a six-week-long therapist-guided online therapy versus self-help internet-based therapy for COVID-19-induced anxiety and depression: open-label, pragmatic, randomized controlled trial. JMIR Ment Health.

[224] Levin W, Campbell DR, McGovern KB, Gau JM, Kosty DB, Seeley JR, Lewinsohn PM (2010). A computer-assisted depression intervention in primary care. Psychol Med.

[225] Ferrari AJ, Charlson FJ, Norman RE, Patten SB, Freedman G, Murray CJ, Vos T, Whiteford HA (2013). Burden of depressive disorders by country, sex, age, and year: findings from the global burden of disease study 2010. PLoS Med.

[226] Kuehner C (2017). Why is depression more common among women than among men?. Lancet Psychiatry.

[227] Lim GY, Tam WW, Lu Y, Ho CS, Zhang MW, Ho RC (2018). Prevalence of depression in the community from 30 countries between 1994 and 2014. Sci Rep.

[228] (2000). Women's mental health evidence based review. World Health Organization.

[229] Yu S (2018). Uncovering the hidden impacts of inequality on mental health: a global study. Transl Psychiatry.

[230] Rungreangkulkij S, Rujiraprasert N (2019). Gender inequality identified as an underlying cause of depression in Thai women. J Int Women Stud.

[231] Rafful C, Medina-Mora ME, Borges G, Benjet C, Orozco R (2012). Depression, gender, and the treatment gap in Mexico. J Affect Disord.

[232] Krumm S, Checchia C, Koesters M, Kilian R, Becker T (2017). Men's views on depression: a systematic review and metasynthesis of qualitative research. Psychopathology.

[233] Seidler ZE, Dawes AJ, Rice SM, Oliffe JL, Dhillon HM (2016). The role of masculinity in men's help-seeking for depression: a systematic review. Clin Psychol Rev.

[234] Otten D, Tibubos AN, Schomerus G, Brähler E, Binder H, Kruse J, Ladwig K, Wild PS, Grabe HJ, Beutel ME (2021). Similarities and differences of mental health in women and men: a systematic review of findings in three large German cohorts. Front Public Health.

[235] Winkler D, Pjrek E, Kasper S (2005). Anger attacks in depression--evidence for a male depressive syndrome. Psychother Psychosom.

[236] Beutel ME, Brähler E, Wiltink J, Kerahrodi JG, Burghardt J, Michal M, Schulz A, Wild PS, Münzel T, Schmidtmann I, Lackner KJ, Pfeiffer N, Borta A, Tibubos AN (2018). New onset of depression in aging women and men: contributions of social, psychological, behavioral, and somatic predictors in the community. Psychol Med.

[237] Perna L, Mielck A, Lacruz ME, Emeny RT, Holle R, Breitfelder A, Ladwig KH (2012). Socioeconomic position, resilience, and health behaviour among elderly people. Int J Public Health.

[238] Angst J, Gamma A, Gastpar M, Lépine JP, Mendlewicz J, Tylee A, Depression S (2002). Gender differences in depression. epidemiological findings from the European DEPRES I and II studies. Eur Arch Psychiatry Clin Neurosci.

[239] Häfner S, Emeny R, Lacruz M, Baumert J, Herder C, Koenig W, Thorand B, Ladwig K (2011). Association between social isolation and inflammatory markers in depressed and non-depressed individuals: results from the MONICA/KORA study. Brain Behav Immun.

[240] Smedley A, Smedley BD (2005). Race as biology is fiction, racism as a social problem is real: anthropological and historical perspectives on the social construction of race. Am Psychol.

[241] Husain A, Hodge DR (2016). Islamically modified cognitive behavioral therapy: enhancing outcomes by increasing the cultural congruence of cognitive behavioral therapy self-statements. Int Soc Work.

[242] Bernal G, Jiménez-Chafey MI, Domenech Rodríguez MM (2009). Cultural adaptation of treatments: a resource for considering culture in evidence-based practice. Prof Psychol Res Pr.

[243] Ratcliff KS (2017). The Social Determinants of Health: Looking Upstream.

[244] Carmona-Huerta J, Durand-Arias S, Rodriguez A, Guarner-Catalá C, Cardona-Muller D, Madrigal-de-León E, Alvarado R (2021). Community mental health care in Mexico: a regional perspective from a mid-income country. Int J Ment Health Syst.

[245] Wainberg ML, Scorza P, Shultz JM, Helpman L, Mootz JJ, Johnson KA, Neria Y, Bradford JE, Oquendo MA, Arbuckle MR (2017). Challenges and opportunities in global mental health: a research-to-practice perspective. Curr Psychiatry Rep.

[246] Kiekens WJ, la Roi C, Dijkstra JK (2021). Sexual identity disparities in mental health among U.K. adults, U.S. adults, and U.S. adolescents: examining heterogeneity by race/ethnicity. Psychol Sex Orientat Gend Divers.

[247] Lee H, Singh GK (2021). Monthly trends in self-reported health status and depression by race/ethnicity and socioeconomic status during the COVID-19 pandemic, United States, April 2020 - May 2021. Ann Epidemiol.

[248] Weller BE, Blanford KL, Butler AM (2018). Estimated prevalence of psychiatric comorbidities in U.S. adolescents with depression by race/ethnicity, 2011-2012. J Adolesc Health.

[249] Le HN, Boyd RC, Lara MA, Richards CS, O'Hara MW (2014). Treatment of depressive disorders and comorbidity in ethnic minority groups. The Oxford Handbook of Depression and Comorbidity.

[250] Wentzel J, van der Vaart R, Bohlmeijer ET, van Gemert-Pijnen JE (2016). Mixing online and face-to-face therapy: how to benefit from blended care in mental health care. JMIR Ment Health.

[251] Erekson DM, Lambert MJ, Eggett DL (2015). The relationship between session frequency and psychotherapy outcome in a naturalistic setting. J Consult Clin Psychol.

[252] Omylinska‐Thurston J, Karkou V, Parsons A, Nair K, Dubrow‐Marshall L, Starkey J, Thurston S, Dudley‐Swarbrick I, Sharma S (2020). Arts for the Blues: the development of a new evidence‐based creative group psychotherapy for depression. Couns Psychother Res.

[253] Martin C, Iqbal Z, Airey ND, Marks L (2022). Improving access to psychological therapies (IAPT) has potential but is not sufficient: how can it better meet the range of primary care mental health needs?. Br J Clin Psychol.

[254] Omylinska‐Thurston J, McMeekin A, Walton P, Proctor G (2019). Clients' perceptions of unhelpful factors in CBT in IAPT serving a deprived area of the UK. Couns Psychother Res.

[255] Farish-Edwards F, Parsons AS, Starkey J, Dubrow-Marshall L, Thurston SD, Omylinska-Thurston J, Karkou V, Prescott J (2022). Digitising creative psychological therapy: arts for the blues (a4b). Digital Innovations for Mental Health Support.

[256] Eisenstadt M, Liverpool S, Infanti E, Ciuvat RM, Carlsson C (2021). Mobile apps that promote emotion regulation, positive mental health, and well-being in the general population: systematic review and meta-analysis. JMIR Ment Health.

[257] Fordham B, Sugavanam T, Edwards K, Stallard P, Howard R, das Nair R, Copsey B, Lee H, Howick J, Hemming K, Lamb SE (2021). The evidence for cognitive behavioural therapy in any condition, population or context: a meta-review of systematic reviews and panoramic meta-analysis. Psychol Med.

[258] Marshall JM, Dunstan DA, Bartik W (2021). Smartphone psychological therapy during COVID-19: a study on the effectiveness of five popular mental health apps for anxiety and depression. Front Psychol.

