# Correction: Digital Psychotherapies for Adults Experiencing Depressive Symptoms: Systematic Review and Meta-Analysis

**DOI:** 10.2196/67439

**Published:** 2024-10-21

**Authors:** Joanna Omylinska-Thurston, Supritha Aithal, Shaun Liverpool, Rebecca Clark, Zoe Moula, January Wood, Laura Viliardos, Edgar Rodríguez-Dorans, Fleur Farish-Edwards, Ailsa Parsons, Mia Eisenstadt, Marcus Bull, Linda Dubrow-Marshall, Scott Thurston, Vicky Karkou

**Affiliations:** 1 School of Health and Society University of Salford Manchester United Kingdom; 2 Faculty of Health, Social Care and Medicine Edge Hill University Ormskirk United Kingdom; 3 Faculty of Nursing, Midwifery and Palliative Care King's College London London United Kingdom; 4 School of Health and Social Science University of Edinburgh Edinburgh United Kingdom; 5 Evidence Based Practice Unit University College London London United Kingdom; 6 Faculty of Life Sciences and Education University of South Wales Newport United Kingdom

In “Digital Psychotherapies for Adults Experiencing Depressive Symptoms: Systematic Review and Meta-Analysis” (JMIR Ment Health. 2024 Sep 30:11:e55500. doi: 10.2196/55500) the authors noted one error.

The surname of author JO-T has been revised from:

Omylinska Thurston

To:

Omylinska-Thurston

Furthermore, the author’s two mentions in the body of the paper have been revised from:

JOT

To:

JO-T

The correction will appear in the online version of the paper on the JMIR Publications website on October 21, 2024, together with the publication of this correction notice. Because this was made after submission to PubMed, PubMed Central, and other full-text repositories, the corrected article has also been resubmitted to those repositories.

