# Supplementary material for: Digital Psychotherapies for Adults Experiencing Depressive Symptoms: Systematic Review and Meta-Analysis
Source: JMIR Ment Health. 2024 Sep 30;11:e55500. doi: 10.2196/55500 (PMC11474132; doi:10.2196/55500)
Supplement: Multimedia Appendix 1 [file mental_v11i1e55500_app1.docx]

**Studies used in systematic review, references for appendices 2 and 3**

[1] Ahmedani BK, Belville-Robertson T, Hirsch A, Jurayj A. An Online Mental Health and Wellness Intervention Supplementing Standard Care of Depression and Anxiety. *Archives of Psychiatric Nursing*. 2016;*30*(6):666–670. [doi:10.1016/j.apnu.2016.03.003](https://doi.org/10.1016/j.apnu.2016.03.003)

[2] Al-Alawi M, McCall RK, Sultan A, et al. Efficacy of a Six-Week-Long Therapist-Guided Online Therapy Versus Self-help Internet-Based Therapy for COVID-19-Induced Anxiety and Depression: Open-label, Pragmatic, Randomized Controlled Trial. *JMIR Mental Health*. 2021;*8*(2):e26683. [doi:10.2196/26683](https://doi.org/10.2196/26683)

[3] Alavi N, Hirji A, Sutton C, Naeem F. Online CBT Is Effective in Overcoming Cultural and Language Barriers in Patients With Depression. *Journal of psychiatric practice*. 2016;*22*(1):2–8. [doi:10.1097/PRA.0000000000000119](https://doi.org/10.1097/PRA.0000000000000119)

[4] Alavi N, Moghimi E, Stephenson C, Gutierrez G, Jagayat J, Kumar A, Shao Y, Miller S, Yee CS, Stefatos A, Gholamzadehmir M, Abbaspour Z, Shirazi A, Gizzarelli T, Khan F, Patel C, Patel A, Yang M, Omrani M. Comparison of online and in-person cognitive behavioral therapy in individuals diagnosed with major depressive disorder: a non-randomized controlled trial. *Front Psychiatry*. 2023;14:1113956. [doi:10.3389/fpsyt.2023.1113956](https://doi/10.3389/fpsyt.2023.1113956)

[5] Amer NA, Shohieb SM, Eladrosy WM, Elbakry HM, Elrazek SMA. Sokoon: A Gamification-Based Cognitive Behavioral Therapy Application – An Application for Depression, Stress, and Anxiety. *International Journal of Gaming and Computer-Mediated Simulations*. 2023;*15*(1):1–26. [doi:10.4018/IJGCMS.324098](https://doi.org/10.4018/IJGCMS.324098)

[6] Andrews B, Klein B, Corboy D, McLaren S, Watson S. Video chat therapist assistance in an adaptive digital intervention for anxiety and depression: Reflections from participants and therapists. *Professional Psychology: Research and Practice.* 2023;*54*(6):418–429. [doi:10.1037/pro0000527](https://psycnet.apa.org/doi/10.1037/pro0000527)

[7] Andrews B, Klein B, Van Nguyen H, Corboy D, McLaren S, Watson S. Efficacy of a Digital Mental Health Biopsychosocial Transdiagnostic Intervention With or Without Therapist Assistance for Adults With Anxiety and Depression: Adaptive Randomized Controlled Trial. *J Med Internet Res*. 2023;25:e45135. [doi:10.2196/45135](https://doi/10.2196/45135)

[8] Anguera JA, Gunning FM, Areán PA. Improving late life depression and cognitive control through the use of therapeutic video game technology: A proof-of-concept randomized trial. *Depression and anxiety*, 2017;*34*(6):508–517. [doi:10.1002/da.22588](https://doi.org/10.1002/da.22588)

[9] Anguera JA, Jordan JT, Castaneda D, Gazzaley A, Areán PA. Conducting a fully mobile and randomised clinical trial for depression: access, engagement and expense. *BMJ Innovations*. 2015;*2*(1):14–21. [doi:10.1136/bmjinnov-2015-000098](https://doi.org/10.1136/bmjinnov-2015-000098)

[10] Araghi NM, Zarei MA, Saei S, Yousefi Nodeh HR, Mahmoudi E. The effect of online cognitive behavioral therapy on depressive symptoms in recovered patients with COVID-19. *J Educ Health Promot*. 2022;11:70. [doi:10.4103/jehp.jehp_727_21](https://doi/10.4103/jehp.jehp_727_21)

[11] Arean PA, Hallgren KA, Jordan JT, et al. The Use and Effectiveness of Mobile Apps for Depression: Results From a Fully Remote Clinical Trial. *Journal of Medical Internet Research*. 2016;*18*(12):e330. [doi:10.2196/jmir.6482](https://doi.org/10.2196/jmir.6482)

[12] Baikie KA, Geerligs L, Wilhelm K. Expressive writing and positive writing for participants with mood disorders: An online randomized controlled trial. *Journal of Affective Disorders*. 2012;*136*(3):310–319. [doi:10.1016/j.jad.2011.11.032](https://doi.org/10.1016/j.jad.2011.11.032)

[13] Bantjes J, Kazdin AE, Cuijpers P, Breet E, Dunn-Coetzee M, Davids C, Stein DJ, Kessler RC. A Web-Based Group Cognitive Behavioral Therapy Intervention for Symptoms of Anxiety and Depression Among University Students: Open-Label, Pragmatic Trial. *JMIR Ment Health*. 2021;8(5):e27400. [doi:10.2196/27400](https://doi/10.2196/27400)

[14] Birney AJ, Gunn R, Russell JK, Ary DV. MoodHacker Mobile Web App With Email for Adults to Self-Manage Mild-to-Moderate Depression: Randomized Controlled Trial. *JMIR mHealth and uHealth*. 2016;*4*(1):e8. [doi:10.2196/mhealth.4231](https://doi.org/10.2196/mhealth.4231)

[15] Bisby MA, Balakumar T, Scott AJ, Titov N, Dear BF. An online therapist-guided ultra-brief treatment for depression and anxiety: a randomized controlled trial. *Psychol Med.* 2024;54(5):902-913. [doi:10.1017/S003329172300260X](https://doi/10.1017/S003329172300260X)

[16] Blackwell SE, Browning M, Mathews A, et al. Positive Imagery-Based Cognitive Bias Modification as a Web-Based Treatment Tool for Depressed Adults: A Randomized Controlled Trial. *Clinical Psychological Science*. 2015;*3*(1):91–111. [doi:10.1177/2167702614560746](https://doi.org/10.1177/2167702614560746)

[17] Boggs JM, Beck A, Felder JN, Dimidjian S, Metcalf CA, Segal ZV. Web-based intervention in mindfulness meditation for reducing residual depressive symptoms and relapse prophylaxis: a qualitative study. *Journal of Medical Internet Research*. 2014;*16*(3):e87. [doi:10.2196/jmir.3129](https://doi.org/10.2196/jmir.3129)

[18] Bowie CR, Gupta M, Holshausen K, Jokic R, Best M, Milev R. Cognitive remediation for treatment-resistant depression: effects on cognition and functioning and the role of online homework. *The Journal of nervous and mental disease*. 2013;*201*(8):680–685. [doi:10.1097/NMD.0b013e31829c50](https://doi.org/10.1097/NMD.0b013e31829c50)

[19] Bowler JO, Mackintosh B, Dunn BD, Mathews A, Dalgleish T, Hoppitt L. A Comparison of Cognitive Bias Modification for Interpretation and Computerized Cognitive Behavior Therapy: Effects on Anxiety, Depression, Attentional Control, and Interpretive Bias. *Journal of Consulting and Clinical Psychology*. 2012;*80*(6):1021–1033. [doi:10.1037/a0029932](https://doi.org/10.1037/a0029932)

[20] Broglia E, Millings A, Barkham M. Counseling With Guided Use of a Mobile Well-Being App for Students Experiencing Anxiety or Depression: Clinical Outcomes of a Feasibility Trial Embedded in a Student Counseling Service. *JMIR mHealth and uHealth*. 2019;*7*(8):e14318. [doi:10.2196/14318](https://doi.org/10.2196/14318)

[21] Buntrock C, Ebert D, Lehr D, et al. (2015). Effectiveness of a Web-Based Cognitive Behavioural Intervention for Subthreshold Depression: Pragmatic Randomised Controlled Trial. *Psychotherapy and Psychosomatics*. 2015;*84*(6):348–358. [doi:10.1159/000438673](https://doi.org/10.1159/000438673)

[22] Calkins AW, McMorran KE, Siegle GJ, Otto MW. The Effects of Computerized Cognitive Control Training on Community Adults with Depressed Mood. *Behavioural and Cognitive Psychotherapy*, 2015;*43*(5):578–589. [doi:10.1017/S1352465814000046](https://doi.org/10.1017/S1352465814000046)

[23] Christensen H, Farrer L, Batterham PJ, Mackinnon A, Griffiths KM, Donker T. The effect of a web-based depression intervention on suicide ideation: secondary outcome from a randomised controlled trial in a helpline. *BMJ open*. 2013;*3*(6):e002886. [doi:10.1136/bmjopen-2013-002886](https://doi.org/10.1136/bmjopen-2013-002886)

[24] Clarke J, Proudfoot J, Birch MR, et al. Effects of mental health self-efficacy on outcomes of a mobile phone and web intervention for mild-to-moderate depression, anxiety and stress: secondary analysis of a randomised controlled trial. *BMC Psychiatry*. 2014;*14*(1):272. [doi:10.1186/s12888-014-0272-1](https://doi.org/10.1186/s12888-014-0272-1)

[25] Cluxton-Keller F, Buteau J, Williams M, Stolte P, Monroe-Cassel M, Bruce M. Engaging rural young mothers in a technology-based intervention for depression. *Child & Youth Services*. 2019;*40*(2):158–183. [doi:10.1080/0145935X.2018.1561264](https://doi.org/10.1080/0145935X.2018.1561264)

[26] Collins S, Byrne M, Hawe J, O’Reilly G. Evaluation of a computerized cognitive behavioural therapy programme, MindWise (2.0), for adults with mild‐to‐moderate depression and anxiety. *British Journal of Clinical Psychology*. 2017;*57*(2):255–269. [doi:10.1111/bjc.12165](https://doi.org/10.1111/bjc.12165)

[27] Currie SL, McGrath PJ, Day V. Development and usability of an online CBT program for symptoms of moderate depression, anxiety, and stress in post-secondary students. *Computers in Human Behavior*. 2010;*26*(6):1419–1426. [doi:10.1016/j.chb.2010.04.020](https://doi.org/10.1016/j.chb.2010.04.020)

[28] Danaher BG, Milgrom J, Seeley JR, et al. Web-Based Intervention for Postpartum Depression: Formative Research and Design of the MomMoodBooster Program. *JMIR Research Protocols*. 2012;*1*(2):e18. [doi:10.2196/resprot.2329](https://doi.org/10.2196/resprot.2329)

[29] Danaher BG, Milgrom J, Seeley JR, et al. MomMoodBooster web-based intervention for postpartum depression: feasibility trial results. *Journal of Medical Internet Research*. 2013;*15*(11):e242. [doi:10.2196/jmir.2876](https://doi.org/10.2196/jmir.2876)

[30] Darnell D, Pullmann MD, Hull TD, Chen S, Areán P. Predictors of Disengagement and Symptom Improvement Among Adults With Depression Enrolled in Talkspace, a Technology-Mediated Psychotherapy Platform: Naturalistic Observational Study. *JMIR Form Res*. 2022;6(6):e36521. [doi:10.2196/36521](https://doi/10.2196/36521)

[31] de Graaf LE, Hollon SD, Huibers MJ. Predicting outcome in computerized cognitive behavioral therapy for depression in primary care: A randomized trial. *Journal of consulting and clinical psychology*. 2010;*78*(2):184–189. [doi:10.1037/a0018324](https://doi.org/10.1037/a0018324)

[32] de Graaf L, Gerhards SA, Arntz A, et al. One-year follow-up results of unsupported online computerized cognitive behavioural therapy for depression in primary care: A randomized trial. *Journal of Behavior Therapy and Experimental Psychiatry*. 2011;*42*(1):89–95. [doi:10.1016/j.jbtep.2010.07.003](https://doi.org/10.1016/j.jbtep.2010.07.003)

[33] Dehn LB, Kater L, Piefke M, Botsch M, Driessen M, Beblo T. Training in a comprehensive everyday-like virtual reality environment compared to computerized cognitive training for patients with depression. *Computers in Human Behavior*. 2018;*79*:40–52. [doi:10.1016/j.chb.2017.10.019](https://doi.org/10.1016/j.chb.2017.10.019)

[34] Dimidjian S, Beck A, Felder JN, Boggs JM, Gallop R, Segal ZV. Web-based Mindfulness-based Cognitive Therapy for reducing residual depressive symptoms: An open trial and quasi-experimental comparison to propensity score matched controls. *Behaviour Research and Therapy*. 2014;*63*:83–89. [doi:10.1016/j.brat.2014.09.004](https://doi.org/10.1016/j.brat.2014.09.004)

[35] Douma M, Maurice-Stam H, Gorter B, et al. Online psychosocial group intervention for parents: Positive effects on anxiety and depression. *Journal of pediatric psychology*. 2020;*46*(2):123–134. [doi:10.1093/jpepsy/jsaa102](https://doi.org/10.1093/jpepsy/jsaa102)

[36] Duarte A, Walker S, Littlewood E, et al. Cost-effectiveness of computerized cognitive–behavioural therapy for the treatment of depression in primary care: findings from the Randomised Evaluation of the Effectiveness and Acceptability of Computerised Therapy (REEACT) trial. *Psychological Medicine*. 2017;*47*(10):1825–1835. [doi:10.1017/S0033291717000289](https://doi.org/10.1017/S0033291717000289)

[37] Ebert DD, Buntrock C, Lehr D, et al. Effectiveness of web- and mobile-based treatment of subthreshold depression with adherence-focused guidance. A single-blind randomised controlled trial. *Behavior Therapy*. 2018;*49*(1):71–83. [doi:10.1016/j.beth.2017.05.004](https://doi.org/10.1016/j.beth.2017.05.004)

[38] Eichenberg C, Schott M, Sawyer A, Aumayr G, Plößnig M. Feasibility and Conceptualization of an e-Mental Health Treatment for Depression in Older Adults: Mixed-Methods Study. *JMIR Aging*, 2018;*1*(2):e10973. [doi:10.2196/10973](https://doi.org/10.2196/10973)

[39] Ekberg S, Barnes RK, Kessler DS, Malpass A, Shaw, ARG. Managing clients’ expectations at the outset of online Cognitive Behavioural Therapy (CBT) for depression. *Health Expectations : an International Journal of Public Participation in Health Care and Health Policy*. 2016;*19*(3):557–569. [doi:10.1111/hex.12227](https://doi.org/10.1111/hex.12227)

[40] El Morr C, Ritvo P, Ahmad F, Moineddin R. Effectiveness of an 8-Week Web-Based Mindfulness Virtual Community Intervention for University Students on Symptoms of Stress, Anxiety, and Depression: Randomized Controlled Trial. *JMIR Mental Health*. 2020;*7*(7):e18595. [doi:10.2196/18595](https://doi.org/10.2196/18595)

[41] Ellis LA, Campbell AJ, Sethi S, O'Dea BM. Comparative randomized trial of an online cognitive-behavioral therapy program and an online support group for depression and anxiety. *Journal of Cybertherapy and Rehabilitation.* 2011;*4*(4):461–467.

[42] Eriksson MCM, Kivi M, Hange D, et al. Long-term effects of Internet-delivered cognitive behavioral therapy for depression in primary care - the PRIM-NET controlled trial. *Scandinavian Journal of Primary Health Care*. 2017;*35*(2):126–136. [doi:10.1080/02813432.2017.1333299](https://doi.org/10.1080/02813432.2017.1333299)

[43] Farrer L, Christensen H, Griffiths KM, Mackinnon A. Web-based cognitive behavior therapy for depression with and without telephone tracking in a national helpline: secondary outcomes from a randomized controlled trial. *Journal of medical Internet research*. 2012;*14*(3):e68. [doi:10.2196/jmir.1859](https://doi.org/10.2196/jmir.1859)

[44] Fatori D, Zuccolo P, Xavier MO, Matijasevich A, Polanczyk GV. Smartphone-assisted online brief cognitive behavioral therapy to treat maternal depression: findings of a randomized controlled trial. *Braz J Psychiatry*. 2023;45(1):50-53. [doi:10.47626/1516-4446-2022-2679](https://doi/10.47626/1516-4446-2022-2679)

[45] Felder J, Dimidjian S, Beck A, Boggs JM, Segal Z. Mindful mood balance: a case report of Web-based treatment of residual depressive symptoms. *Permanente Journal*. 2014;*18*(4):58–62. [doi:10.7812/TPP/14-019](https://doi.org/10.7812/TPP/14-019)

[46] Figueroa CA, DeMasi O, Hernandez-Ramos R, Aguilera A. Who Benefits Most from Adding Technology to Depression Treatment and How? An Analysis of Engagement with a Texting Adjunct for Psychotherapy. *Telemedicine journal and e-health : the official journal of the American Telemedicine Association*. 2021;*27*(1):39–46. [doi:10.1089/tmj.2019.0248](https://doi.org/10.1089/tmj.2019.0248)

[47] Fogarty AS, Proudfoot J, Whittle EL, et al. Preliminary Evaluation of a Brief Web and Mobile Phone Intervention for Men With Depression: Men’s Positive Coping Strategies and Associated Depression, Resilience, and Work and Social Functioning. *JMIR Mental Health*. 2017;*4*(3):e33. [doi:10.2196/mental.7769](https://doi.org/10.2196/mental.7769)

[48] Forman-Hoffman VL, Nelson BW, Ranta K, Nazander A, Hilgert O, de Quevedo J. Significant reduction in depressive symptoms among patients with moderately-severe to severe depressive symptoms after participation in a therapist-supported, evidence-based mobile health program delivered via a smartphone app. *Internet Interv*. 2021;25:100408. [doi:10.1016/j.invent.2021](https://doi/10.1016/j.invent.2021)

[49] Forman-Hoffman VL, Sihvonen S, Wielgosz J, Kuhn E, Nelson BW, Peiper NC, Gould CE. Therapist-supported digital mental health intervention for depressive symptoms: A randomized clinical trial. *J Affect Disord*. 2024;349:494-501. [doi:10.1016/j.jad.2024.01.057](https://doi/10.1016/j.jad.2024.01.057)

[50] Fuller-Tyszkiewicz M, Richardson B, Klein B, et al. A Mobile App-Based Intervention for Depression: End-User and Expert Usability Testing Study. *JMIR Mental Health*. 2018;*5*(3):e54. [doi:10.2196/mental.9445](https://doi.org/10.2196/mental.9445)

[51] Fundoiano-Hershcovitz Y, Breuer Asher I, Ritholz MD, Feniger E, Manejwala O, Goldstein P. Specifying the Efficacy of Digital Therapeutic Tools for Depression and Anxiety: Retrospective, 2-Cohort, Real-World Analysis. *J Med Internet Res*. 2023;25:e47350. [doi:10.2196/47350](https://doi/10.2196/47350)

[52] Gega L, Smith J, Reynolds S. Cognitive behaviour therapy (CBT) for depression by computer vs. therapist: Patient experiences and therapeutic processes. *Psychotherapy Research*. 2013;*23*(2):218–231. [doi:10.1080/10503307.2013.766941](https://doi.org/10.1080/10503307.2013.766941)

[53] Geraedts AS, van Dongen JM, Kleiboer AM, et al. Economic Evaluation of a Web-Based Guided Self-Help Intervention for Employees With Depressive Symptoms: Results of a Randomized Controlled Trial. *Journal of occupational and environmental medicine*. 2015;*57*(6):666–675. [doi:10.1097/JOM.0000000000000423](https://doi.org/10.1097/JOM.0000000000000423)

[54] Gerhards SA, Abma TA, Arntz A, et al. Improving adherence and effectiveness of computerised cognitive behavioural therapy without support for depression: a qualitative study on patient experiences. *Journal of affective disorders*. 2011;*129*(1-3):117–125. [doi:10.1016/j.jad.2010.09.012](https://doi.org/10.1016/j.jad.2010.09.012)

[55] Gerhards S, Graaf E, de Jacobs L, et al. Economic evaluation of online computerised cognitive-behavioural therapy without support for depression in primary care: randomised trial. *British Journal of Psychiatry*. 2010;*196*(4):310–318. [doi:10.1192/bjp.bp.109.065748](https://doi.org/10.1192/bjp.bp.109.065748)

[56] Gilbody S, Littlewood E, Hewitt C, et al. Computerised cognitive behaviour therapy (cCBT) as treatment for depression in primary care (REEACT trial): large scale pragmatic randomised controlled trial. *BMJ (Online)*. 2015;*351*:h5627. [doi:10.1136/bmj.h5627](https://doi.org/10.1136/bmj.h5627)

[57] Goldin PR, Lindholm R, Ranta K, Hilgert O, Helteenvuori T, Raevuori A. Feasibility of a Therapist-Supported, Mobile Phone-Delivered Online Intervention for Depression: Longitudinal Observational Study. *JMIR Formative Research*. 2019;*3*(1):e11509. [doi:10.2196/11509](https://doi.org/10.2196/11509)

[58] Gomà M, Arias-Pujol E, Prims E, Ferrer J, Lara S, Glover V, Martinez M, Llairó A, Nanzer N. Internet-based interdisciplinary therapeutic group (Grupo Interdisciplinar Online, GIO) for perinatal anxiety and depression-a randomized pilot study during COVID-19. *Arch Womens Ment Health* [doi:10.1007/s00737-023-01412-2](https://doi/10.1007/s00737-023-01412-2)

[59] Gräfe V, Berger T, Hautzinger M, et al. Health economic evaluation of a web-based intervention for depression: the EVIDENT-trial, a randomized controlled study. *Health Economics Review*. 2019;*9*(1):16–13. [doi:10.1186/s13561-019-0233-y](https://doi.org/10.1186/s13561-019-0233-y)

[60] Hald GM, Ciprić A, Øverup CS, et al. Randomized controlled trial study of the effects of an online divorce platform on anxiety, depression, and somatization. *Journal of family psychology : JFP : journal of the Division of Family Psychology of the American Psychological Association (Division 43)*. 2020;*34*(6):740–751. [doi:10.1037/fam0000635](https://doi.org/10.1037/fam0000635)

[61] Hatcher S, Whittaker R, Patton M, et al. Web-based Therapy Plus Support by a Coach in Depressed Patients Referred to Secondary Mental Health Care: Randomized Controlled Trial. *JMIR Mental Health*. 2018;*5*(1):e5. [doi:10.2196/mental.8510](https://doi.org/10.2196/mental.8510)

[62] Heller HM, Hoogendoorn AW, Honig A, Broekman BF, van Straten A. The effectiveness of a guided Internet-based tool for the treatment of depression and anxiety in pregnancy (Mamakits online): Randomized controlled trial. *Journal of Medical Internet Research*. 2020;*22*(3):e15172. [doi:10.2196/15172](https://doi.org/10.2196/15172)

[63] Hirsch A, Luellen J, Holder JM, Steinberg G, Dubiel T, Blazejowskyj A, Schladweiler K. Managing Depressive Symptoms in the Workplace Using a Web-Based Self-Care Tool: A Pilot Randomized Controlled Trial. *JMIR Research Protocols*. 2017;*6*(4):e51. [doi:10.2196/resprot.7203](https://doi.org/10.2196/resprot.7203)

[64] Høifødt RS, Lillevoll KR, Griffiths KM, et al. The clinical effectiveness of web-based cognitive behavioral therapy with face-to-face therapist support for depressed primary care patients: randomized controlled trial. *Journal of Medical Internet Research*. 2013;*15*(8):e153. [doi:10.2196/jmir.2714](https://doi.org/10.2196/jmir.2714)

[65] Hollinghurst S, Peters TJ, Kaur S, Wiles N, Lewisand G, Kessler D. Cost-effectiveness of therapist-delivered online cognitive–behavioural therapy for depression: randomised controlled trial. *British Journal of Psychiatry*. 2010;*197*(4):297–304. doi:10.1192/bjp.bp.109.073080

[66] Holst A, Nejati S, Björkelund C, et al. Patients’ experiences of a computerised self-help program for treating depression - a qualitative study of Internet mediated cognitive behavioural therapy in primary care. *Scandinavian Journal of Primary Health Care*. 2017;*35*(1):46–53. [doi:10.1080/02813432.2017.1288813](https://doi.org/10.1080/02813432.2017.1288813)

[67] Hur JW, Kim B, Park D, Choi SW. A Scenario-Based Cognitive Behavioral Therapy Mobile App to Reduce Dysfunctional Beliefs in Individuals with Depression: A Randomized Controlled Trial. *Telemedicine journal and e-health : the official journal of the American Telemedicine Association*. 2018;*24*(9):710–716. [doi:10.1089/tmj.2017.0214](https://doi.org/10.1089/tmj.2017.0214)

[68] Iacoviello BM, Murrough JW, Hoch MM, et al. A randomized, controlled pilot trial of the Emotional Faces Memory Task: a digital therapeutic for depression. *NPJ Digital Medicine*. 2019;*1*(1). [doi:10.1038/s41746-018-0025-5](https://doi.org/10.1038/s41746-018-0025-5)

[69] İme, Y. The Effect of Online Cognitive Behavioral Group Counseling on Anxiety, Depression, Stress and Resilience in Maraş-Centered Earthquake Survivors. *Journal of Rational-Emotive and Cognitive-Behavior Therapy*. *Journal of Rational-Emotive & Cognitive-Behavior Therapy*. 2023. [doi:10.1007/s10942-023-00526-x](https://doi.org/10.1007/s10942-023-00526-x)

[70] Jannati N, Mazhari S, Ahmadian L, Mirzaee M. Effectiveness of an app-based cognitive behavioral therapy program for postpartum depression in primary care: A randomized controlled trial. *International Journal of Medical Informatics (Shannon, Ireland)*. 2020;*141*:104145. [doi:10.1016/j.ijmedinf.2020.104145](https://doi.org/10.1016/j.ijmedinf.2020.104145)

[71] Jelinek L, Arlt S, Moritz S, Schröder J, Westermann S, Cludius B. Brief Web-Based Intervention for Depression: Randomized Controlled Trial on Behavioral Activation. *Journal of Medical Internet Research*. 2020;*22*(3):e15312. [doi:10.2196/15312](https://doi.org/10.2196/15312)

[72] Jonassaint CR, Gibbs P, Belnap BH, Karp JF, Abebe KZ, Rollman BL. Engagement and outcomes for a computerised cognitive-behavioural therapy intervention for anxiety and depression in African Americans. *BJPsych Open*. 2017;*3*(1):1–5. [doi:10.1192/bjpo.bp.116.003657](https://doi.org/10.1192/bjpo.bp.116.003657)

[73] Keller A, Babl A, Berger T, Schindler L. Efficacy of the web-based PaarBalance program on relationship satisfaction, depression and anxiety – A randomized controlled trial. *Internet Interventions : the Application of Information Technology in Mental and Behavioural Health*. 2021; *23*:100360. [doi:10.1016/j.invent.2020.100360](https://doi.org/10.1016/j.invent.2020.100360)

[74] Kenter RM, Cuijpers P, Beekman A, van Straten A. Effectiveness of a web-based guided self-help intervention for outpatients with a depressive disorder: Short-term results from a randomized controlled trial. *Journal of Medical Internet Research*. 2016;*18*(3):e80. [doi:10.2196/jmir.4861](https://doi.org/10.2196/jmir.4861)

[75] Kim DR, Hantsoo L, Thase ME, Sammel M, Epperson CN. Computer-assisted cognitive behavioral therapy for pregnant women with major depressive disorder. *Journal of women's health (2002)*. 2014;*23*(10):842–848. [doi:10.1089/jwh.2014.4867](https://doi.org/10.1089/jwh.2014.4867)

[76] Kingston J, Becker L, Woeginger J, Ellett L. A randomised trial comparing a brief online delivery of mindfulness-plus-values versus values only for symptoms of depression: Does baseline severity matter? *Journal of Affective Disorders*. 2020;*276*:936–944. [doi:10.1016/j.jad.2020.07.087](https://doi.org/10.1016/j.jad.2020.07.087)

[77] Kivi M, Eriksson MCM, Hange D, et al. Internet-Based Therapy for Mild to Moderate Depression in Swedish Primary Care: Short Term Results from the PRIM-NET Randomized Controlled Trial. *Cognitive Behaviour Therapy*. 2014;*43*(4):289–298. [doi:10.1080/16506073.2014.921834](https://doi.org/10.1080/16506073.2014.921834)

[78] Kladnitski N, Smith J, Allen A, Andrews G, Newby JM. Online mindfulness-enhanced cognitive behavioural therapy for anxiety and depression: Outcomes of a pilot trial. *Internet Interventions : the Application of Information Technology in Mental and Behavioural Health*. 2018;*13*:41–50. [doi:10.1016/j.invent.2018.06.003](https://doi.org/10.1016/j.invent.2018.06.003)

[79] Klein JP, Berger T, Schröder J, et al. Effects of a Psychological Internet Intervention in the Treatment of Mild to Moderate Depressive Symptoms: Results of the EVIDENT Study, a Randomized Controlled Trial. *Psychotherapy and Psychosomatics*. 2016;*85*(4):218–228. [doi:10.1159/000445355](https://doi.org/10.1159/000445355)

[80] Knowles SE, Lovell K, Bower P, Gilbody S, Littlewood E, Lester H. Patient experience of computerised therapy for depression in primary care. *BMJ open*. 2015;*5*(11):e008581. [doi:10.1136/bmjopen-2015-008581](https://doi.org/10.1136/bmjopen-2015-008581)

[81] Krämer LV, Grünzig SD, Baumeister H, Ebert DD, Bengel J. Effectiveness of a Guided Web-Based Intervention to Reduce Depressive Symptoms before Outpatient Psychotherapy: A Pragmatic Randomized Controlled Trial. *Psychother Psychosom*. 2021;90(4):233-242. [doi:10.1159/000515625](https://doi/10.1159/000515625)

[82] Krusche A, Cyhlarova E, Williams JMG. Mindfulness online: an evaluation of the feasibility of a web-based mindfulness course for stress, anxiety and depression. *BMJ Open*. 2013*3*(11):e003498. [doi:10.1136/bmjopen-2013-003498](https://doi.org/10.1136/bmjopen-2013-003498)

[83] Lappalainen P, Langrial S, Oinas-Kukkonen H, Tolvanen A, Lappalainen R. Web-Based Acceptance and Commitment Therapy for Depressive Symptoms With Minimal Support: A Randomized Controlled Trial. *Behavior Modification*. 2015;*39*(6):805–834. [doi:10.1177/0145445515598142](https://doi.org/10.1177/0145445515598142)

[84] Lara MA, Tiburcio M, Aguilar Abrego A, Sánchez-Solís A. A four-year experience with a Web-based self-help intervention for depressive symptoms in Mexico. *Revista panamericana de salud publica = Pan American journal of public health*. 2014;*35*(5-6):399–406.

[85] Lemma A, Fonagy P. Feasibility study of a psychodynamic online group intervention for depression. *Psychoanalytic Psychology. 2013;30*(3):367–380. [doi:10.1037/a0033239](https://psycnet.apa.org/doi/10.1037/a0033239)

[86] Levesque DA, Van Marter DF, Schneider RJ, et al. Randomized Trial of a Computer-Tailored Intervention for Patients with Depression. *American Journal of Health Promotion. 2011;26*(2):77–89. [doi:10.4278/ajhp.090123-QUAN-27](https://doi.org/10.4278/ajhp.090123-QUAN-27)

[87] Levin W, Campbell DR, McGovern KB, et al. A computer-assisted depression intervention in primary care. *Psychological medicine*. 2011;*41*(7):1373–1383. [doi:10.1017/S0033291710001935](https://doi.org/10.1017/S0033291710001935)

[88] Liu J, Duan W, Xiao Z, Wu Y. The effectiveness of online group mindfulness-based cognitive therapy for outpatients with depression in China. *J Affect Disord*. 2024;351:387-391.

[89] Löbner M, Pabst A, Stein J, et al. Computerized cognitive behavior therapy for patients with mild to moderately severe depression in primary care: A pragmatic cluster randomized controlled trial (@ktiv). *Journal of Affective Disorders*. 2018;*238*:317–326. [doi:10.1016/j.jad.2018.06.008](https://doi.org/10.1016/j.jad.2018.06.008)

[90] Lokman S, Leone SS, Sommers-Spijkerman MPJ, van der Poel A, Smit F, Boon B. Complaint-Directed Mini-Interventions for Depressive Complaints: A Randomized Controlled Trial of Unguided Web-Based Self-Help Interventions. *Journal of Medical Internet Research*. 2017;*19*(1):e4. [doi:10.2196/jmir.6581](https://doi.org/10.2196/jmir.6581)

[91] Lu Y, Li Y, Huang Y, Zhang X, Wang J, Wu L, Cao F. Effects and Mechanisms of a Web- and Mobile-Based Acceptance and Commitment Therapy Intervention for Anxiety and Depression Symptoms in Nurses: Fully Decentralized Randomized Controlled Trial. *J Med Internet Res*. 2023;25:e51549. doi:10.2196/51549

[92] Lucassen MFG, Hatcher S, Stasiak K, Fleming T, Shepherd M, Merry SN. The views of lesbian, gay and bisexual youth regarding computerised self-help for depression: An exploratory study. *Advances in Mental Health*. 2013;*12*(1):22–33. [doi:10.5172/jamh.2013.12.1.22](https://doi.org/10.5172/jamh.2013.12.1.22)

[93] Lüdtke T, Westermann S, Pult LK, Schneider BC, Pfuhl G, Moritz, S. Evaluation of a brief unguided psychological online intervention for depression: A controlled trial including exploratory moderator analyses. *Internet Interventions : the Application of Information Technology in Mental and Behavioural Health*. 2018;*13*:73–81. [doi:10.1016/j.invent.2018.06.004](https://doi.org/10.1016/j.invent.2018.06.004)

[94] Lutz W, Arndt A, Rubel J, et al. Defining and Predicting Patterns of Early Response in a Web-Based Intervention for Depression. *Journal of Medical Internet Research*. 2017;*19*(6):e206. [doi:10.2196/jmir.7367](https://doi.org/10.2196/jmir.7367)

[95] MacLean S, Corsi DJ, Litchfield S, et al. Coach-Facilitated Web-Based Therapy Compared With Information About Web-Based Resources in Patients Referred to Secondary Mental Health Care for Depression: Randomized Controlled Trial. *Journal of Medical Internet Research*. 2020;*22*(6):e15001. [doi:10.2196/15001](https://doi.org/10.2196/15001)

[96] Mahoney A, Li I, Haskelberg H, Millard M, Newby JM. The uptake and effectiveness of online cognitive behaviour therapy for symptoms of anxiety and depression during COVID-19. *J Affect Disord*. 2021;292:197-203.

[97] Mahoney A, Shiner CT, Grierson AB, Sharrock MJ, Loughnan SA, Harrison V, Millard M. Online cognitive behaviour therapy for maternal antenatal and postnatal anxiety and depression in routine care. *J Affect Disord*. 2023;338:121-128.

[98] Marcelle ET, Nolting L, Hinshaw SP, Aguilera A. Effectiveness of a Multimodal Digital Psychotherapy Platform for Adult Depression: A Naturalistic Feasibility Study. *JMIR mHealth and uHealth* 2019;*7*(1):e10948. [doi:10.2196/10948](https://doi.org/10.2196/10948)

[99] McCloud T, Jones R, Lewis G, Bell V, Tsakanikos E. Effectiveness of a Mobile App Intervention for Anxiety and Depression Symptoms in University Students: Randomized Controlled Trial. *JMIR mHealth and uHealth*. 2020;*8*(7):e15418. [doi:10.2196/15418](https://doi.org/10.2196/15418)

[100] McMurchie W, Macleod F, Power K, Laidlaw K, Prentice N. Computerised cognitive behavioural therapy for depression and anxiety with older people: a pilot study to examine patient acceptability and treatment outcome. *International Journal of Geriatric Psychiatry*. 2013;*28*(11):1147–1156. [doi:10.1002/gps.3935](https://doi.org/10.1002/gps.3935)

[101] Moberg C, Niles A, Beermann D. Guided Self-Help Works: Randomized Waitlist Controlled Trial of Pacifica, a Mobile App Integrating Cognitive Behavioral Therapy and Mindfulness for Stress, Anxiety, and Depression. *Journal of Medical Internet Research*. 2019;*21*(6):e12556–e12556. [doi:10.2196/12556](https://doi.org/10.2196/12556)

[102] Moghimi E, Stephenson C, Agarwal A, Nikjoo N, Malakouti N, Layzell G, O'Riordan A, Jagayat J, Shirazi A, Gutierrez G, Khan F, Patel C, Yang M, Omrani M, Alavi N. Efficacy of an Electronic Cognitive Behavioral Therapy Program Delivered via the Online Psychotherapy Tool for Depression and Anxiety Related to the COVID-19 Pandemic: Pre-Post Pilot Study. *JMIR Ment Health*. 2023;10:e51102. doi:10.2196/51102

[103] Mohr DC, Duffecy J, Jin L, Ludman EJ, Lewis A, Begale M, McCarthy M. Multimodal e-mental health treatment for depression: a feasibility trial. *Journal of medical Internet research*. 2010;*12*(5):e48. [doi:10.2196/jmir.1370](https://doi.org/10.2196/jmir.1370)

[104] Mohr DC, Schueller SM, Tomasino KN, et al. Comparison of the Effects of Coaching and Receipt of App Recommendations on Depression, Anxiety, and Engagement in the IntelliCare Platform: Factorial Randomized Controlled Trial. *Journal of Medical Internet Research*. 2019;*21*(8):e13609. [doi:10.2196/13609](https://doi.org/10.2196/13609)

[105] Mol M, Dozeman E, Provoost S, van Schaik A, Riper H, Smit JH. Behind the Scenes of Online Therapeutic Feedback in Blended Therapy for Depression: Mixed-Methods Observational Study. *Journal of medical Internet research*. 2018;*20*(5):e174. [doi:10.2196/jmir.9890](https://doi.org/10.2196/jmir.9890)

[106] Morthland M, Shah A, Meadows JT, Scogin F. Development of an audio and computer cognitive behavioral therapy for depression in older adults. *Aging & Mental Health*. 2020;*24*(8):1207–1215. [doi:10.1080/13607863.2019.1609901](https://doi.org/10.1080/13607863.2019.1609901)

[107] Moskowitz JT, Addington EL, Shiu E, Bassett SM, Schuette S, Kwok I, Freedman ME, Leykin Y, Saslow LR, Cohn MA, Cheung EO. Facilitator Contact, Discussion Boards, and Virtual Badges as Adherence Enhancements to a Web-Based, Self-guided, Positive Psychological Intervention for Depression: Randomized Controlled Trial. *J Med Internet Res*. 2021;23(9):e25922. doi:10.2196/25922

[108] Motter JN, Grinberg A, Lieberman DH, Iqnaibi WB, Sneed JR. Computerized cognitive training in young adults with depressive symptoms: Effects on mood, cognition, and everyday functioning. *Journal of Affective Disorders*. 2019;*245*:28–37. [doi:10.1016/j.jad.2018.10.109](https://doi.org/10.1016/j.jad.2018.10.109)

[109] Nakao S, Nakagawa A, Oguchi Y, et al. Web-Based Cognitive Behavioral Therapy Blended With Face-to-Face Sessions for Major Depression: Randomized Controlled Trial. *Journal of Medical Internet Research*. 2018;*20*(9):e10743. [doi:10.2196/10743](https://doi.org/10.2196/10743)

[110] Nelson CB, Abraham KM, Walters H, Pfeiffer PN, Valenstein M. Integration of peer support and computer-based CBT for veterans with depression. *Computers in Human Behavior*. 2014;*31*:57–64. [doi:10.1016/j.chb.2013.10.012](https://doi.org/10.1016/j.chb.2013.10.012)

[111] Nogami W, Nakagawa A, Kato N, Sasaki Y, Kishimoto T, Horikoshi M, Mimura M. Efficacy and Acceptability of Remote Cognitive Behavioral Therapy for Patients With Major Depressive Disorder in Japanese Clinical Settings: A Case Series. *Cogn Behav Pract*. 2022.

[112] Oehler C, Görges F, Rogalla M, Rummel-Kluge C, Hegerl U. Efficacy of a Guided Web-Based Self-Management Intervention for Depression or Dysthymia: Randomized Controlled Trial With a 12-Month Follow-Up Using an Active Control Condition. *Journal of Medical Internet Research*. 2020;*22*(7):e15361. [doi:10.2196/15361](https://doi.org/10.2196/15361)

[113] Ofoegbu TO, Asogwa U, Otu MS, Ibenegbu C, Muhammed A, Eze, B. Efficacy of guided internet-assisted intervention on depression reduction among educational technology students of Nigerian universities. *Medicine (Baltimore)*. 2020;*99*(6):e18774. [doi:10.1097/MD.0000000000018774](https://doi.org/10.1097/MD.0000000000018774)

[114] Openshaw DK, Pfister R, Silverbaltt H, Moen D. Providing Mental Health Services to Women Diagnosed with Depression in Rural Utah Communities: Using Technologically Assisted Psychotherapeutic Intervention as the Delivery Medium. *Journal of Rural Mental Health*. 2011;*35*(1):23–31. [doi:10.1037/h0094782](https://doi.org/10.1037/h0094782)

[115] Orr LC, Graham AK, Mohr DC, Greene CJ. Engagement and Clinical Improvement Among Older Adult Primary Care Patients Using a Mobile Intervention for Depression and Anxiety: Case Studies. *JMIR Mental Health*. 2020;*7*(7):e16341. [doi:10.2196/16341](https://doi.org/10.2196/16341)

[116] Otared N, Moharrampour NG, Vojoudi B, Najafabadi AJ. A Group-based Online Acceptance and Commitment therapy Treatment for Depression, anxiety symptomns and quality of Life in Healthcare Workers during COVID-19 Pandemic: A Randomized Controlled Trial. *International Journal of Psychology and Psychological Therapy.* 2021;21(3):399-411.

[117] O’Toole MS, Arendt MB, Pedersen CM. Testing an App-Assisted Treatment for Suicide Prevention in a Randomized Controlled Trial: Effects on Suicide Risk and Depression. *Behavior Therapy*. 2019;*50*(2):421–429. [doi:10.1016/j.beth.2018.07.007](https://doi.org/10.1016/j.beth.2018.07.007)

[118] Pettitt AK, Nelson BW, Forman-Hoffman VL, Goldin PR, Peiper NC. Longitudinal outcomes of a therapist-supported digital mental health intervention for depression and anxiety symptoms: A retrospective cohort study. *Psychol Psychother*. 2024. doi:10.1111/papt.12517

[119] Pfeiffer PN, Pope B, Houck M, et al. Effectiveness of Peer-Supported Computer-Based CBT for Depression Among Veterans in Primary Care. *Psychiatric Services (Washington, D.C.)*. 2020;*71*(3):256–262. [doi:10.1176/appi.ps.201900283](https://doi.org/10.1176/appi.ps.201900283)

[120] Phillips R, Schneider J, Molosankwe I, et al. Randomized controlled trial of computerized cognitive behavioural therapy for depressive symptoms: effectiveness and costs of a workplace intervention. *Psychological medicine*. 2014;*44*(4):741–752. [doi:10.1017/S0033291713001323](https://doi.org/10.1017/S0033291713001323)

[121] Pinto MD, Greenblatt AM, Hickman RL, Rice HM, Thomas TL, Clochesy, JM. Assessing the Critical Parameters of eSMART-MH: A Promising Avatar-Based Digital Therapeutic Intervention to Reduce Depressive Symptoms. *Perspectives in Psychiatric Care*. 2015;*52*(3):157–168. [doi:10.1111/ppc.12112](https://doi.org/10.1111/ppc.12112)

[122] Pinto MD, Hickman RL, Clochesy J, Buchner M. Avatar-based depression self-management technology: promising approach to improve depressive symptoms among young adults. *Applied Nursing Research*. 2013;*26*(1):45–48. [doi:10.1016/j.apnr.2012.08.003](https://doi.org/10.1016/j.apnr.2012.08.003)

[123] Pots WT, Fledderus M, Meulenbeek PA, ten Klooster PM, Schreurs KM, Bohlmeijer ET. Acceptance and commitment therapy as a web-based intervention for depressive symptoms: randomised controlled trial. *The British journal of psychiatry : the journal of mental science*. 2016;*208*(1):69–77. [doi:10.1192/bjp.bp.114.146068](https://doi.org/10.1192/bjp.bp.114.146068)

[124] Pots WT, Trompetter HR, Schreurs KM, Bohlmeijer ET. How and for whom does web-based acceptance and commitment therapy work? Mediation and moderation analyses of web-based ACT for depressive symptoms. *BMC Psychiatry*. 2016;*16*(157):158. [doi.10.1186/s12888-016-0841-6](https://doi.org/10.1186/s12888-016-0841-6)

[125] Pratap A, Renn BN, Volponi J, et al. Using Mobile Apps to Assess and Treat Depression in Hispanic and Latino Populations: Fully Remote Randomized Clinical Trial. *Journal of Medical Internet Research*. 2018;*20*(8):e10130. [doi.10.2196/10130](https://doi.org/10.2196/10130)

[126] Preschl B, Maercker A, Wagner B. The working alliance in a randomized controlled trial comparing online with face-to-face cognitive-behavioral therapy for depression. *BMC Psychiatry*. 2011;*11*(1):189. [doi:10.1186/1471-244X-11-189](https://doi.org/10.1186/1471-244X-11-189)

[127] Proudfoot J, Clarke J, Birch MR, et al Impact of a mobile phone and web program on symptom and functional outcomes for people with mild-to-moderate depression, anxiety and stress: a randomised controlled trial. *BMC Psychiatry*. 2013;*13*(1):312. [doi:10.1186/1471-244X-13-312](https://doi.org/10.1186/1471-244X-13-312)

[128] Proyer RT, Gander F, Wellenzohn S, Ruch W. Positive psychology interventions in people aged 50-79 years: long-term effects of placebo-controlled online interventions on well-being and depression. *Aging & Mental Health*. 2014;*18*(8):997–1005. [doi:10.1080/13607863.2014.899978](https://doi.org/10.1080/13607863.2014.899978)

[129] Pugh NE, Hadjistavropoulos HD, Fuchs CM. Internet therapy for postpartum depression: a case illustration of emailed therapeutic assistance. *Archives of women's mental health*. 2014;*17*(4):327–337. [doi:10.1007/s00737-014-0439-2](https://doi.org/10.1007/s00737-014-0439-2)

[130] Reins JA, Boß L, Lehr D, Berking M, Ebert DD. The more I got, the less I need? Efficacy of Internet-based guided self-help compared to online psychoeducation for major depressive disorder. *Journal of Affective Disorders*. 2019;*246*:695–705. [doi:10.1016/j.jad.2018.12.065](https://doi.org/10.1016/j.jad.2018.12.065)

[131] Richards D, Enrique A, Eilert N, et al. A pragmatic randomized waitlist-controlled effectiveness and cost-effectiveness trial of digital interventions for depression and anxiety. *NPJ Digital Medicine*. 2020;*3*(1):85. [doi:10.1038/s41746-020-0293-8](https://doi.org/10.1038/s41746-020-0293-8)

[132] Richards D, Timulak L. Client-identified helpful and hindering events in therapist-delivered vs. self-administered online cognitive-behavioural treatments for depression in college students. *Counselling Psychology Quarterly*. 2012;*25*(3):251–262. [doi:10.1080/09515070.2012.703129](https://doi.org/10.1080/09515070.2012.703129)

[133] Richards D, Timulak L. Satisfaction with therapist-delivered vs. self-administered online cognitive behavioural treatments for depression symptoms in college students. *British Journal of Guidance & Counselling*. 2013;*41*(2):193–207. [doi:10.1080/03069885.2012.726347](https://doi.org/10.1080/03069885.2012.726347)

[134] Richards D, Timulak L, Hevey D. A comparison of two online cognitive-behavioural interventions for symptoms of depression in a student population: The role of therapist responsiveness. *Counselling and Psychotherapy Research*. 2012;*13*(3):184–193. [doi:10.1080/14733145.2012.733715](https://doi.org/10.1080/14733145.2012.733715)

[135] Richter LE, Machleit-Ebner A, Scherbaum N, Bonnet U. How Effective is a Web-Based Mental Health Intervention (Deprexis) in the Treatment of Moderate and Major Depressive Disorders when started during Routine Psychiatric Inpatient Treatment as an Adjunct Therapy? A Pragmatic Parallel-Group Randomized Controlled Trial. *Fortschr Neurol Psychiatr*;91(7-08):297-310. doi:10.1055/a-1826-2888

[136] Ritvo P, Knyahnytska Y, Pirbaglou M, Wang W, Tomlinson G, Zhao H, Linklater R, Bai S, Kirk M, Katz J, Harber L, Daskalakis Z. Online Mindfulness-Based Cognitive Behavioral Therapy Intervention for Youth With Major Depressive Disorders: Randomized Controlled Trial. *J Med Internet Res*. 2021;23(3):e24380. doi:10.2196/24380

[137] Rollman BL, Herbeck Belnap B, Abebe KZ, et al. Effectiveness of Online Collaborative Care for Treating Mood and Anxiety Disorders in Primary Care: A Randomized Clinical Trial. *JAMApsychiatry*. 2018;*75*(1):56–64. [doi:10.1001/jamapsychiatry.2017.3379](https://doi.org/10.1001/jamapsychiatry.2017.3379)

[138] Rotondi A, Belnap B, Rothenberger S, Feldman R, Hanusa B, Rollman B. Predictors of Use and Drop Out From a Web-Based Cognitive Behavioral Therapy Program and Health Community for Depression and Anxiety in Primary Care Patients: Secondary Analysis of a Randomized Controlled Trial. *JMIR Ment Health*. 2024;11:e52197 doi

[139] Rozbroj T, Lyons A, Pitts M, Mitchell A, Christensen H. Improving self-help e-therapy for depression and anxiety among sexual minorities: an analysis of focus groups with lesbians and gay men. *Journal of Medical Internet Research*. 20115;*17*(3):e66. [doi:10.2196/jmir.4013](https://doi.org/10.2196/jmir.4013)

[140] Sampson E. Implementing Digital Cognitive-Behavioral Therapy for Major Depressive Disorder in Routine Psychiatric Appointments: A Pilot Project in a Rural Population. *J Psychosoc Nurs Ment Health Serv*. 2023;61(10):44-51. doi:10.3928/02793695-20230424-01

[141] Sandoval LR, Buckey JC, Ainslie R, Tombari M, Stone W, Hegel MT. Randomized Controlled Trial of a Computerized Interactive Media-Based Problem Solving Treatment for Depression. *Behavior Therapy*. 2017;*48*(3):413–425. [doi:10.1016/j.beth.2016.04.001](https://doi.org/10.1016/j.beth.2016.04.001)

[142] Schlosser DA, Campellone TR, Truong B, et al. The feasibility, acceptability, and outcomes of PRIME‐D: A novel mobile intervention treatment for depression. *Depression and Anxiety*. 2017;*34*(6):546–554. [doi:10.1002/da.22624](https://doi.org/10.1002/da.22624)

[143] Schneider BC, Schröder J, Berger T, et al. Corrigendum to ``Bridging the “digital divide”: A comparison of use and effectiveness of an online intervention for depression between Baby Boomers and Millennials’’. *Journal of Affective Disorders*. 2018;*241*:635–635. [doi:10.1016/j.jad.2018.05.031](https://doi.org/10.1016/j.jad.2018.05.031)

[144] Schneider J, Sarrami Foroushani P, Grime, P, Thornicroft G. Acceptability of online self-help to people with depression: users’ views of MoodGYM versus informational websites. *Journal of Medical Internet Research*. 2014;*16*(3):e90. [doi:10.2196/jmir.2871](https://doi.org/10.2196/jmir.2871)

[145] Schueller S, Mohr D. Initial Field Trial of a Coach-Supported Web-Based Depression Treatment. *EAI Endorsed Transactions on Serious Games*. 2015; *2*(7). [doi:10.4108/icst.pervasivehealth.2015.260115](https://doi.org/10.4108/icst.pervasivehealth.2015.260115)

[146] Schuster R, Kalthoff I, Walther A, et al. Effects, Adherence, and Therapists’ Perceptions of Web- and Mobile-Supported Group Therapy for Depression: Mixed-Methods Study. *Journal of Medical Internet Research*. 2019;*21*(5):e11860. [doi:10.2196/11860](https://doi.org/10.2196/11860)

[147] Schuster R, Sigl S, Berger T, Laireiter AR. Patients’ Experiences of Web- and Mobile-Assisted Group Therapy for Depression and Implications of the Group Setting: Qualitative Follow-Up Study. *JMIR Mental Health*. 2018;*5*(3):e49. [doi:10.2196/mental.9613](https://doi.org/10.2196/mental.9613)

[148] Segal ZV, Dimidjian S, Beck A, et al. Outcomes of Online Mindfulness-Based Cognitive Therapy for Patients With Residual Depressive Symptoms: A Randomized Clinical Trial. *JAMA psychiatry*. 2020;*77*(6):563–573. [doi:10.1001/jamapsychiatry.2019.4693](https://doi.org/10.1001/jamapsychiatry.2019.4693)

[149] Sergeant S, Mongrain M. An Online Optimism Intervention Reduces Depression in Pessimistic Individuals. *Journal of Consulting and Clinical Psychology*. 2014;*82*(2):263–274. [doi:10.1037/a0035536](https://doi.org/10.1037/a0035536)

[150] Seshu U, Khan HA, Bhardwaj M, et al. A qualitative study on the use of mobile-based intervention for perinatal depression among perinatal mothers in rural Bihar, India. *International Journal of Social Psychiatry*. 2020;*67*(5):467–471. [doi:10.1177/0020764020966003](https://doi.org/10.1177/0020764020966003)

[151] Sethi S. Treating Youth Depression and Anxiety: A Randomised Controlled Trial Examining the Efficacy of Computerised versus Face-to-face Cognitive Behaviour Therapy. *Australian Psychologist*. 2013;*48*(4):249–257. [doi:10.1111/ap.12006](https://doi.org/10.1111/ap.12006)

[152] Shah A, Morthland M, Scogin F, Presnell A, DiNapoli EA, DeCoster J, Yang X. Audio and Computer Cognitive Behavioral Therapy for Depressive Symptoms in Older Adults: A Pilot Randomized Controlled Trial. *Behavior Therapy*. 2018;*49*(6):904–916. [doi:10.1016/j.beth.2018.06.002](https://doi.org/10.1016/j.beth.2018.06.002)

[153] Sharry J, Davidson R, McLoughlin O, Doherty G.. A service-based evaluation of a therapist-supported online cognitive behavioral therapy program for depression. *Journal of Medical Internet Research*. 2013;*15*(6):e121. [doi:10.2196/jmir.2248](https://doi.org/10.2196/jmir.2248)

[154] Shkel J, Green G, Le S, Kaveladze B, Marcotte V, Rushton K, Nguyen T, Schueller SM. Understanding Users' Experiences of a Novel Web-Based Cognitive Behavioral Therapy Platform for Depression and Anxiety: Qualitative Interviews From Pilot Trial Participants. *JMIR Form Res*. 2023;7:e46062. doi:10.2196/46062

[155] Silva Almodovar A, Surve S, Axon DR, Cooper D, Nahata MC. Self-Directed Engagement with a Mobile App (Sinasprite) and Its Effects on Confidence in Coping Skills, Depression, and Anxiety: Retrospective Longitudinal Study. *JMIR mHealth and uHealth*. 2018;*6*(3):e64. [doi:10.2196/mhealth.9612](https://doi.org/10.2196/mhealth.9612)

[156] Silverstone PH, Rittenbach K, Suen VYM, et al. Depression Outcomes in Adults Attending Family Practice Were Not Improved by Screening, Stepped-Care, or Online CBT during a 12-Week Study when Compared to Controls in a Randomized Trial. *Frontiers in Psychiatry*. 2017;*8*:32–32. [doi:10.3389/fpsyt.2017.00032](https://doi.org/10.3389/fpsyt.2017.00032)

[157] Spates CR, Kalata AH, Ozeki S, Stanton CE, Peters S. Initial Open Trial of a Computerized Behavioral Activation Treatment for Depression. *Behavior Modification*. 2012;*37*(3):259–297. [doi:10.1177/0145445512455051](https://doi.org/10.1177/0145445512455051)

[158] Stearns-Yoder KA, Ryan AT, Smith AA, Forster JE, Barnes SM, Brenner LA. Computerized Cognitive Behavioral Therapy Intervention for Depression Among Veterans: Acceptability and Feasibility Study. *JMIR Form Res*. 2022;6(4):e31835. doi:10.2196/31835

[159] Thase ME, McCrone P, Barrett MS, et al. (2020). Improving Cost-effectiveness and Access to Cognitive Behavior Therapy for Depression: Providing Remote-Ready, Computer-Assisted Psychotherapy in Times of Crisis and Beyond. *Psychotherapy and Psychosomatics*. 2020;*89*(5):307–313. [doi:10.1159/000508143](https://doi.org/10.1159/000508143)

[160] Thase ME, Wright JH, Eells TD, et al. Improving the Efficiency of Psychotherapy for Depression: Computer-Assisted Versus Standard CBT. *The American journal of psychiatry*. *2018;175*(3):242–250. [doi:10.1176/appi.ajp.2017.17010089](https://doi.org/10.1176/appi.ajp.2017.17010089)

[161] Titov N, Dear BF, Staples LG, et al. MindSpot Clinic: An Accessible, Efficient, and Effective Online Treatment Service for Anxiety and Depression. *Psychiatric Services (Washington, D.C.)*. 2015;*66*(10):1043–1050. [doi:10.1176/appi.ps.201400477](https://doi.org/10.1176/appi.ps.201400477)

[162] Titzler I, Egle V, Berking M, Gumbmann C, Ebert DD. Blended Psychotherapy: Treatment Concept and Case Report for the Integration of Internet- and Mobile-Based Interventions into Brief Psychotherapy of Depressive Disorders. *Verhaltenstherapie*. 2022;32(1): 230-244. [doi:10.6084/m9.figshare.10505021](https://doi.org/10.6084/m9.figshare.10505021)

[163] Tulbure BT, Rusu A, Sava FA, Sălăgean N, Farchione TJ. A Web-Based Transdiagnostic Intervention for Affective and Mood Disorders: Randomized Controlled Trial. *JMIR Mental Health*. *2018;5*(2):e36. [doi:10.2196/mental.8901](https://doi.org/10.2196/mental.8901)

[164] van der Zanden R, Curie K, Van Londen M, Kramer J, Steen G, Cuijpers P. Web-based depression treatment: associations of clients' word use with adherence and outcome. *Journal of affective disorders*. 2014;*160*:10–13. [doi:10.1016/j.jad.2014.01.005](https://doi.org/10.1016/j.jad.2014.01.005)

[165] van der Zanden R, Galindo-Garre F, Curie K, Kramer J, Cuijpers P. Online cognitive-based intervention for depression: exploring possible circularity in mechanisms of change. *Psychological Medicine*. 2013;*44*(6):1159–1170. [doi:10.1017/S003329171300175X](https://doi.org/10.1017/S003329171300175X)

[166] van der Zanden R, Kramer J, Gerrits R, Cuijpers P. Effectiveness of an online group course for depression in adolescents and young adults: a randomized trial. *Journal of medical Internet research*. 2012;*14*(3):e86. [doi:10.2196/jmir.2033](https://doi.org/10.2196/jmir.2033)

[167] Venkatesan A, Rahimi L, Kaur M, Mosunic C. Digital Cognitive Behavior Therapy Intervention for Depression and Anxiety: Retrospective Study. *JMIR Mental Health*. 2020;*7*(8):e21304. [doi:10.2196/21304](https://doi.org/10.2196/21304)

[168] Vernmark K, Lenndin J, Bjärehed J, et al. Internet administered guided self-help versus individualized e-mail therapy: A randomized trial of two versions of CBT for major depression. *Behaviour Research and Therapy*. 2010;*48*(5):368–376. [doi:10.1016/j.brat.2010.01.005](https://doi.org/10.1016/j.brat.2010.01.005)

[169] Wagner B, Horn AB, Maercker A. Internet-based versus face-to-face cognitive-behavioral intervention for depression: A randomized controlled non-inferiority trial. *Journal of Affective Disorders*. 2014;*152*:113–121. [doi:10.1016/j.jad.2013.06.032](https://doi.org/10.1016/j.jad.2013.06.032)

[170] Wahle F, Kowatsch T, Fleisch E, Rufer M, Weidt S. Mobile Sensing and Support for People With Depression: A Pilot Trial in the Wild. *JMIR mHealth and uHealth*. 2016;*4*(3):e111. [doi:10.2196/mhealth.5960](https://doi.org/10.2196/mhealth.5960)

[171] Walsh S, Szymczynska P, Taylor SJC, Priebe S. The acceptability of an online intervention using positive psychology for depression: A qualitative study. *Internet Interventions : the Application of Information Technology in Mental and Behavioural Health*. 2018;*13*:60–66. <https://doi.org/10.1016/j.invent.2018.07.003>

[172] Wang L, Miller L. Assessment and Disruption of Ruminative Episodes to Enhance Mobile Cognitive Behavioral Therapy Just-in-Time Adaptive Interventions in Clinical Depression: Pilot Randomized Controlled Trial. *JMIR Form Res*. 2023;7:e37270. doi:10.2196/37270

[173] Warmerdam E, van Straten A, Jongsma J, Twisk JW, Cuijpers P. (2010). Online cognitive behavioral therapy and problem-solving therapy for depressive symptoms: Exploring mechanisms of change.  *Journal of Behavior Therapy and Experimental Psychiatry*. 2010;*41*(1):64–70. [doi:10.1016/j.jbtep.2009.10.003](https://doi.org/10.1016/j.jbtep.2009.10.003)

[174] Watts S, Mackenzie A, Thomas C, Griskaitis A, Mewton L, Williams A, Andrews G. CBT for depression: a pilot RCT comparing mobile phone vs. computer. *BMC Psychiatry*. 2013;*13*(1):49. [doi:10.1186/1471-244X-13-49](https://doi.org/10.1186/1471-244X-13-49)

[175] Welch ES, Weigand A, Hooker JE, et al. Feasibility of Computerized Cognitive‐Behavioral Therapy Combined With Bifrontal Transcranial Direct Current Stimulation for Treatment of Major Depression. *Neuromodulation (Malden, Mass.)*. 2019;*22*(8):898–903. [doi:10.1111/ner.12807](https://doi.org/10.1111/ner.12807)

[176] Westerhof GJ, Lamers SMA, Postel MG, Bohlmeijer ET. Online Therapy for Depressive Symptoms: An Evaluation of Counselor-Led and Peer-Supported Life Review Therapy. *The Gerontologist*. 2017;*59*(1):135–146. [doi:10.1093/geront/gnx140](https://doi.org/10.1093/geront/gnx140)

[177] Whiteside U, Richards J, Steinfeld B, et al. Online cognitive behavioral therapy for depressed primary care patients: a pilot feasibility project. *Permanente Journal*. 2014;*18*(2):21–27. [doi:10.7812/TPP/13-155](https://doi.org/10.7812/TPP/13-155)

[178] Whitton AE, Proudfoot J, Clarke J, et al. Breaking Open the Black Box: Isolating the Most Potent Features of a Web and Mobile Phone-Based Intervention for Depression, Anxiety, and Stress. *JMIR Mental Health*. 2015;*2*(1):e3. [doi:10.2196/mental.3573](https://doi.org/10.2196/mental.3573)

[179] Wijnen BF, Lokman S, Leone S, Evers SM, Smit F. Complaint-Directed Mini-Interventions for Depressive Symptoms: A Health Economic Evaluation of Unguided Web-Based Self-Help Interventions Based on a Randomized Controlled Trial. *Journal of medical Internet research*. 2018;*20*(10):e10455. [doi:10.2196/10455](https://doi.org/10.2196/10455)

[180] Wilhelmsen M, Høifødt RS, Kolstrup N, et al Norwegian general practitioners’ perspectives on implementation of a guided web-based cognitive behavioral therapy for depression: a qualitative study. *Journal of Medical Internet Research*. 2014;*16*(9):e208. [doi:10.2196/jmir.3556](https://doi.org/10.2196/jmir.3556)

[181] Williams C, McClay CA, Martinez R, Morrison J, Haig C, Jones R, Farrand P. Online Cognitive Behavioral Therapy (CBT) Life Skills Program for Depression: Pilot Randomized Controlled Trial. *JMIR Form Res*. 2022;6(2):e30489. doi:10.2196/30489

[182] Wright JH, Owen J, Eells TD, Antle B, Bishop LB, Girdler R, Harris LM, Wright RB, Wells MJ, Gopalraj R, Pendleton ME, Ali S. Effect of Computer-Assisted Cognitive Behavior Therapy vs Usual Care on Depression Among Adults in Primary Care: A Randomized Clinical Trial. *JAMA Netw Open*. 2022;5(2):e2146716. doi:10.1001/jamanetworkopen.2021.46716

[183] Wu MS, Wickham RE, Chen SY, Chen C, Lungu A. Examining the Impact of Digital Components Across Different Phases of Treatment in a Blended Care Cognitive Behavioral Therapy Intervention for Depression and Anxiety: Pragmatic Retrospective Study. *JMIR Form Res*. 2021;5(12):e33452. doi:

[184] Xiang X, Kayser J, Ash S, Zheng C, Sun Y, Weaver A, Dunkle R, Blackburn JA, Halavanau A, Xue J, Himle JA. Web-Based Cognitive Behavioral Therapy for Depression Among Homebound Older Adults: Development and Usability Study. *JMIR Aging*. 2023;6:e47691. doi:10.2196/47691

[185] Yeung A, Wang F, Feng F, et al. Outcomes of an online computerized cognitive behavioral treatment program for treating chinese patients with depression: A pilot study. *Asian Journal of Psychiatry*. 2018;*38*:102–107. [doi:10.1016/j.ajp.2017.11.007](https://doi.org/10.1016/j.ajp.2017.11.007)

[186] Zhang R, Nicholas J, Knapp AA, et al. Clinically Meaningful Use of Mental Health Apps and its Effects on Depression: Mixed Methods Study. *Journal of Medical Internet Research*. 2019;*21*(12):e15644. [doi:10.2196/15644](https://doi.org/10.2196/15644)
