# Supplementary material for: Digital Psychotherapies for Adults Experiencing Depressive Symptoms: Systematic Review and Meta-Analysis
Source: JMIR Ment Health. 2024 Sep 30;11:e55500. doi: 10.2196/55500 (PMC11474132; doi:10.2196/55500)
Supplement: Multimedia Appendix 3 [file mental_v11i1e55500_app3.docx]

**Risk of Bias Grading Tables**

| **Table 5**. Risk of bias grading for studies with an RCT component | | | | | | |
| --- | --- | --- | --- | --- | --- | --- |
| **Author and year** | **Random sequence generation** | **Allocation concealment** | **Blinding of participants and personnel** | **Blinding of outcome assessment** | **Incomplete outcome data addressed** | **Selective reporting** |
| [2] Al-Alawi et al, 2021 | L | L | H | L | L | U |
| [3] Alavi et al,  2016 | L | U | U | U | L | L |
| [6] Andrews et al, 2023 | L | L | H | L | L | L |
| [8] Anguera et al, 2017 | L | U | U | U | L | L |
| [11] Arean et al, 2016 | L | H | H | H | L | L |
| [12] Baikie et al, 2012 | U | U | U | U | L | L |
| [14] Birney at al, 2016 | L | U | U | U | L | L |
| [15] Bisby et al, 2023 | L | L | H | H | L | L |
| [16]  Blackwell al al, 2015 | L | L | L | L | L | L |
| [19] Bowler et al, 2012 | U | U | U | U | L | L |
| [21] Buntrock et al, 2015 | L | U | L | U | L | L |
| [22] Calkins et al, 2015 | L | U | U | U | L | L |
| [31] De Graaf et al, 2010 | L | U | U | U | L | L |
| [35] Douma et al, 2020 | L | H | H | H | L | L |
| [37] Ebert et al, 2018 | L | L | H | L | L | L |
| [41] Ellis et al, 2011 | L | U | U | U | U | H |
| [40] El Morr et al, 2020 | L | L | L | L | L | L |
| [44] Fatori et al, 2023 | L | L | H | L | L | L |
| [49] Forman-Hoffman et al, 2024 | L | U | H | L | L | L |
| [56] Gilbody et al, 2015 | L | H | H | L | L | U |
| [58] Goma et al, 2023 | L | H | H | L | L | U |
| [64] Høifødt et al, 2013 | L | L | L | L | L | L |
| [60] Hald et al, 2020 | L | U | U | L | L | L |
| [61] Hatcher et al, 2018 | L | L | H | L | L | L |
| [62] Heller et al, 2020 | L | L | H | U | L | L |
| [63] Hirsch et al, 2017 | L | L | H | U | U | H |
| [67] Hur et al,  2018 | L | L | U | U | L | U |
| [68] Iacoviello et al, 2018 | L | U | L | L | H | H |
| [69] Ime et al, 2023 | U | H | H | H | L | U |
| [70] Jannati et al, 2020 | L | L | H | U | L | L |
| [71] Jelinek et al, 2020 | L | U | U | U | L | L |
| [77] Kivi et al,  2014 | L | L | U | U | L | L |
| [79] Klein et al, 2016 | U | U | U | U | L | U |
| [73] Keller et al, 2021 | L | L | U | U | L | L |
| [74] Kenter et al, 2016 | L | L | U | U | L | L |
| [76] Kingston et al, 2020 | U | U | L | U | L | L |
| [81] Kramer et al, 2021 | L | U | H | L | L | L |
| [89] Löbner et al, 2019 | L | H | H | L | L | L |
| [93] Ludtke et al, 2018 | L | H | H | H | L | L |
| [83 Lappalainen et al, 2015 | L | U | U | U | L | L |
| [85] Lemma et al, 2013 | U | U | U | U | U | L |
| [86] Levesque et al, 2011 | L | U | U | U | L | L |
| [87] Levin et al, 2011 | L | U | U | U | L | L |
| [91] Lu et al, 2023 | L | L | H | L | L | L |
| [95] MacLean et al, 2020 | L | H | H | H | L | L |
| [99] McCloud et al, 2020 | L | H | H | H | L | L |
| [101] Moberg et al, 2019 | L | H | H | U | L | L |
| [107] Moskowitz et al, 2021 | U | U | H | H | L | L |
| [109] Nakao et al, 2018 | L | H | H | L | L | L |
| [112] Oehler et al, 2020 | L | U | H | U | L | L |
| [116] Otared et al, 2021 | U | U | H | U | U | U |
| [117] O’Toole et al, 2019 | L | L | H | H | L | L |
| [119] Pfeiffer et al, 2020 | L | L | H | H | L | L |
| [120] Phillips et al, 2014 | L | L | L | L | L | H |
| [121] Pinto et al, 2016 | U | U | U | U | L | U |
| [123] Pots et al, 2016 | U | U | U | U | L | L |
| [126] Preschl et al, 2011 | L | H | H | U | L | L |
| [127] Proudfoot et al, 2013 | L | H | H | H | L | L |
| [128] Proyer et al, 2014 | L | U | U | U | L | L |
| [130] Reins et al, 2019 | L | U | U | L | L | H |
| [131] Richards et al, 2020 | L | U | U | H | L | H |
| [131] Richter et al, 2022 | L | H | H | H | L | L |
| [136] Ritvo et al, 2021 | L | U | H | H | L | L |
| [137] Rollman et al, 2018 | L | L | H | L | L | L |
| [141] Sandoval et al, 2015 | L | H | H | H | L | H |
| [143] Schneider et al, 2017 | L | L | H | L | U | L |
| [148] Segal et al, 2020 | L | U | U | L | L | L |
| [149] Sergeant et al, 2014 | L | U | U | U | L | L |
| [151] Sethi et al, 2013 | L | L | H | H | L | L |
| [152] Shah et al, 2018 | L | L | H | L | L | L |
| [156] Silverstone et al, 2017 | L | U | H | H | U | H |
| [160] Thase et al, 2018 | L | U | U | U | L | L |
| [163] Tulbure et al, 2018 | L | U | U | U | U | L |
| [166] van der Zanden et al,  2012 | L | U | U | U | L | L |
| [168] Vernmark et al, 2010 | L | L | U | L | L | L |
| [172] Wang, 2023 | L | L | L | L | L | L |
| [173] Warmerdam et al,  2013 | L | L | U | U | L | L |
| [176] Westerhof et al, 2017 | L | U | U | U | L | L |
| [181] Williams et al, 2022 | L | U | H | L | U | L |
| [182] Wright et al, 2022 | L | U | H | H | L | U |
| [90] Lokman et al, 2017 | L | H | H | U | L | L |
| [185] Yeung et al, 2018 | L | U | U | U | L | L |

| **Table 6.** Quality appraisal grading for studies with qualitative design | | | | | |
| --- | --- | --- | --- | --- | --- |
| **Author and year** | **Approach appropriate for research question** | **Data collection adequate** | **Findings derived from data** | **Result substantiated by data** | **Qualitative data coherence** |
| [17] Boggs et al, 2014 | L | L | L | L | L |
| [27] Curie et al, 2010 | L | L | L | L | L |
| [39] Ekberg et al, 2016 | L | L | L | L | L |
| [92] Lucassen et al, 2013 | L | L | L | L | L |
| [115] Orr et al, 2020 | H | L | H | L | L |
| [129] Pugh et al, 2014 | L | L | L | L | L |
| [139] Rozbroj et al, 2015 | L | L | L | L | L |
| [144] Schneider et al, 2014 | L | L | L | L | L |
| [145] Schueller et al, 2015 | H | L | U | L | L |
| [146] Schuster et al, 2019 | L | L | L | L | L |
| [154] Shkel et al, 2023 | L | L | L | L | L |
| [150] Seshu et al, 2014 | L | L | L | L | L |
| [171] Walsh et al, 2018 | L | L | L | L | L |
| [180] Wilhelmsen et al, 2014 | L | L | L | L | L |

| **Table 7.** Quality appraisal grading for studies with other quantitative design | | | | | |
| --- | --- | --- | --- | --- | --- |
| **Author and year** | **Representative participants** | **Appropriate measures** | **Complete outcome data** | **Confounders accounted for** | **Intervention administered** |
| [1] Ahmedani et al, 2006 | H | L | L | L | L |
| [26] Collins et al, 2017 | L | L | H | U | L |
| [28] Danaher et al, 2013 | L | L | L | L | L |
| [33] Dehn et al, 2018 | U | L | L | L | U |
| [34] Dimidjan et al, 2014 | L | L | L | U | L |
| [46] Figueroa et al, 2021 | U | U | U | U | U |
| [47] Fogarty et al, 2017 | L | L | H | L | H |
| [57] Goldin et al, 2019 | H | L | L | L | L |
| [75] Kim et al, 2014 | L | L | H | L | L |
| [78] Kladnitski et al, 2018 | L | L | L | L | U |
| [100] McMurchie et al, 2013 | L | L | L | L | U |
| [103] Mohr et al, 2010 | L | L | H | L | U |
| [110] Nelson et al, 2014 | L | L | H | L | L |
| [167] Venkatesan et al, 2020 | L | L | H | L | L |
| [170] Wahle et al, 2016 | U | L | H | U | U |

| **Table 8.** Quality appraisal grading for studies with quantitative descriptive statistics | | | | | | | | | | | |
| --- | --- | --- | --- | --- | --- | --- | --- | --- | --- | --- | --- |
| **Author and year** | | **Relevant sampling strategy** | | **Representative sample** | | **Appropriate measurements** | | **Risk of nonresponse bias low** | | **Appropriate statistical analysis** | |
| [5] Amer et al, 2023 | | H | | H | | L | | U | | H | |
| [10] Araghi et al, 2023 | | L | | H | | L | | U | | H | |
| [13] Bantjes et al, 2021 | | L | | L | | L | | H | | L | |
| [25] Cluxton-Keller et al,  2019 | | L | | L | | L | | U | | L | |
| [30] Darnell et al, 2022 | | L | | L | | L | | U | | L | |
| [48] Foreman Hoffman et al, 2021 | | L | | L | | L | | U | | L | |
| [51] Fundoiano Herschcovitz et al, 2023 | | L | | L | | L | | H | | L | |
| [65] Hollinghurst et al, 2010 | | U | | L | | U | | H | | L | |
| [82] Krusche et al, 2013 | | U | | U | | L | | U | | L | |
| [88] Liu et al, 2024 | | L | | U | | L | | U | | L | |
| [98] Mahoney et al, 2021 (the uptake) | | L | | L | | L | | L | | L | |
| [97] Mahoney et al, 2021 (online cognitive) | | L | | L | | L | | L | | L | |
| [98] Marcelle et al, 2019 | | L | | U | | L | | U | | L | |
| [102] Moghimi et al, 2023 | | L | | H | | L | | H | | L | |
| [103] Mohr et al, 2010 | | L | | H | | L | | L | | L | |
| [110] Nelson et al, 2014 | | U | | U | | U | | L | | L | |
| [114] Openshaw et al, 2011 | | L | | U | | L | | H | | L | |
| [118] Pettitt et al, 2024 | | L | | L | | L | | L | | L | |
| [140] Sampson et al, 2023 | | L | | H | | L | | H | | U | |
| [153] Sharry et al, 2013 | | H | | H | | U | | H | | L | |
| [155] Silva-Almodovar et al, 2018 | | L | | H | | H | | H | | L | |
| [158] Stearns-Yoder et al,  2022 | L | | L | | L | | U | | L | |  |
| [177] Whiteside et al, 2014 | | L | | L | | L | | H | | L | |
| [183] Wu et al, 2021 | | L | | L | | L | | L | | L | |
| [184] Xiang et al, 2023 | | L | | H | | L | | L | | L | |

| **Table 9.** Quality appraisal grading for studies with mixed methods design | | | | | |
| --- | --- | --- | --- | --- | --- |
| **Author and year** | **Adequate rationale** | **Integration of components** | **Interpretation of integrated components** | **Adequate address of divergencies and inconsistencies** | **Adhere4nce to quality criteria** |
| [84] Lara et al, 2014 | H | L | H | H | H |
| [29] Danaher et al, 2013 | U | L | L | L | L |
| [38] Eichenberg et al, 2018 | L | L | H | U | U |
| [50] Fuller-Tyskiewicz et al, 2018 | L | L | L | U | L |
| [52] Gega et al, 2013 | L | L | L | U | L |
| [85] Lemma et al, 2013 | L | L | H | U | H |
| [105] Mol et al, 2018 | L | L | L | U | U |
| [106] Morthland et al, 2020 | L | L | L | U | U |
| [110] Nogami et al, 2022 | L | L | L | U | L |
| [133] Richards et al, 2012 | L | L | L | L | U |
| [142] Schlosser et al, 2017 | L | L | L | L | L |
| [145] Schueller et al, 2015 | L | L | U | U | U |
| [146] Schuster et al, 2019 | L | L | L | L | L |
| [162] Titzler et al, 2022 | L | L | L | L | L |
| [186] Zhang et al, 2019 | L | L | L | U | U |
